# Patient Time Spent With Professional Medical Interpreters and the Care Experiences of Patients With Limited English Proficiency

**DOI:** 10.1177/21501319241264168

**Published:** 2024-06-24

**Authors:** Pamela Torresdey, Jacob Chen, Hector P. Rodriguez

**Affiliations:** 1 University of California, Berkeley, CA, USA

**Keywords:** limited English proficiency, interpreter services, language access, immigrant health, clinician-patient communication, community health centers, Medicaid

## Abstract

**Introduction/Objectives:**

More time spent with interpreters may support clinician-patient communication for patients with limited English proficiency (LEP), especially when interpreter support before and after clinical encounters is considered. We assessed whether more time spent with interpreters is associated with better patient-reported experiences of clinician-patient communication and interpreter support among patients with LEP.

**Methods:**

Patients with LEP (n = 338) were surveyed about their experiences with both the clinician and interpreter. Duration of interpreter support during the encounter (in min) and auxiliary time spent before and after encounters supporting patients (in min) were documented by interpreters. Multivariable linear regression models were estimated to assess the association of the time duration of interpreter support and patient experiences of (1) clinician-patient communication, and (2) interpreter support, controlling for patient and encounter characteristics.

**Results:**

The average encounter duration was 47.7 min (standard deviation, SD = 25.1), the average auxiliary time was 43.8 min (SD = 16.4), and the average total interpreter time was 91.1 min (SD = 28.6). LEP patients reported better experiences of interpreter support with a mean score of 97.4 out of 100 (SD = 6.99) compared to clinician-patient communication, with a mean score of 93.7 out of 100 (SD = 14.1). In adjusted analyses, total patient time spent with an interpreter was associated with better patient experiences of clinician-patient communication (β = 7.23, *P* < .01) when auxiliary time spent by interpreters supporting patients before and after the encounter was considered, but not when only the encounter time was considered.

**Conclusions:**

Longer duration of time spent with an interpreter was associated with better clinician-patient communication for patients with LEP when time spent with an interpreter before and after the clinician encounter is considered. Policymakers should consider reimbursing health care organizations for time interpreters spend providing patient navigation and other support beyond clinical encounters.

## Introduction

High-quality clinician-patient communication is an essential part of healthcare delivery, especially for patients with Limited English-language Proficiency (LEP), who often face communication barriers.^1^ Research evidence indicates that better clinician-patient communication is associated with improved technical quality of care and patients’ overall care experiences.^2,3^ When language-concordant care is not possible, support from professional medical interpreters can improve patient comprehension, encourage appropriate healthcare utilization, improve quality of care, and reduce communication errors for patients with LEP.^4,5^ Moreover, ensuring access to professional interpreters can reduce the burden of medical care on family members.^6^

Professional medical interpreters provide support to healthcare organizations in a variety of ways. Some organizations offer interpreter support only for the encounter, while other organizations provide additional interpreter support to patients before and after the encounter to help with patient navigation activities such as orienting patients to their appointment, scheduling follow-up visits, and assisting with understanding medication instructions - all which can extend the duration of interpreter time spent with LEP patients.^7^ Despite the known benefits of professional medical interpretation, there are no policies or suggestions for interpreter time allocation.^4,5^ Limited empirical evidence exists about the relationship between time spent with interpreters and the care experiences of LEP patients receiving care from community health centers (CHCs), who disproportionately serve low-income LEP patients.^8^ The unique contribution of this study is that we examine whether more patient time spent with interpreters is associated with more positive patient care experiences.

To advance evidence, we empirically assessed the extent to which longer duration of patient time spent with interpreters is associated with better clinician-patient communication and patient experiences of interpreter support. Past evidence indicates when patients have positive experiences of care from primary care team members, they also experience high-quality communication with their primary care clinician because the support from care team members help patients understand,^9^ be more willing to communicate during clinical encounters,^10^ and adhere to treatment recommendations from the clinician and assist patients with self-management education outside of clinical encounters.^11^ Consequently, we hypothesized that more patient time spent with interpreters would be associated with better patient experiences of clinician-patient communication because interpreter support improves patient comprehension of information discussed during the encounter. We also hypothesized that patient time spent with an interpreter would be more strongly associated with clinician-patient communication when pre and post visit time is considered, in addition to the encounter time, because the navigation support provided by interpreters can help LEP patients better navigate services and adhere to treatment recommendations.^12,13^

## Methods

### Data Sources

Patient survey data and encounter assessment data from the Department of Health Care Services (DHCS) Medical Interpreter Pilot Program (MIPP) were analyzed. DHCS is responsible for the administration of California’s Medicaid program, called Medi-Cal. MIPP aimed to test the impact of flexible solutions for expanding interpreter services on quality of care for patients with LEP. MIPP eligibility was confirmed based on clinic administrative records of patient English proficiency, meaning only those with documented limited English proficiency were eligible for interpretation services through MIPP. Survey data was collected from patients (n = 338) of 3 California-based CHCs from October 3, 2022 to June 30, 2023. Three CHCs were recruited by DHCS for the MIPP to achieve geographic diversity across the state (Los Angeles, San Diego, and Contra Costa counties). Patients who received MIPP interpreter services for their medical appointment at 1 of the 3 clinics were recruited to complete a patient experience survey. After the clinician encounter, the medical interpreter asked the patient if they were interested in participating in a research survey related to their experiences of receiving professional medical interpreter services. The medical interpreter described the survey, provided a study information sheet, and emphasized that participation was voluntary and there were no consequences for non-participation. Patients were given the option to complete the survey on a private computer at the clinic, on their own device from an email invitation, or over the phone with an interviewer who spoke their language. Participants who opted for a survey over the phone were contacted 2 to 3 weeks after their MIPP-supported appointment by an external researcher in their own language. The survey was translated into 27 languages, including Arabic, Armenian, Burmese, Cambodian, Chinese (Simplified), Chinese (Traditional), Dari, Farsi, Haitian Creole, Hindi, Hmong, Japanese, Karen, Laotian, Oromo, Pashto, Portuguese, Punjabi, Russian, Somali, Spanish, Swahili, Tagalog, Thai, Tigrinya, Ukrainian, and Vietnamese. See the Supplemental Appendix for the content of all translated surveys.

All patients who used MIPP services were eligible for survey recruitment. Researchers primarily administered surveys over the phone (70%) with other patients completing a web-based survey in a private room at the CHC site or on their own device (30%). Patients were emailed or texted a $10 gift card within 48 h of survey completion. From October 2022 to June 2023, 2,422 MIPP survey-eligible patients were informed about the MIPP satisfaction and experience survey. Of 2,422 patients, 720 (29.7%) expressed interest in completing the survey. Of 720 patients who expressed interest, 338 completed surveys (recruitment rate = 46.9%, response rate = 14.0%). All MIPP eligible patients were recruited; however, reasons for non-participation included (1) “patient was not interested” (n = 1636, 55.7%), (2) “interpreter was unable to recruit due to time constraints” (n = 66, 2.2%), and (3) “patient previously participated” (n = 513, 17.5%).

Interpreters completed an encounter documentation form after each MIPP-supported medical interpretation encounter. This form consisted of 15 questions related to patient demographics (language, date of birth, and insurance eligibility) and interpreter service utilization (start and end time, auxiliary time spent with the patient before and after the encounter, modality, clinical services translated, and pilot site).

Data were de-identified to maintain patient privacy and confidentiality and a study-specific patient identifier was used. Encounter assessment data were then merged with patient survey responses for each patient to examine the association of duration of time patient spent with interpreters and patient-reported experiences of care. All study procedures were approved by the [redacted] institutional review board (#2022-05-15393).

## Measures

### Main Independent Variable

The duration of patient time spent with interpreters, measured in minutes, was captured through the encounter assessment data form filled out by interpreters after completing their time with the patient, detailing the start and end time of each encounter as well as auxiliary time spent supporting patients before and after the encounter. Per MIPP policy, encounter time was rounded to the nearest 5-min increment, measured from the time a clinician arrived at the appointment and the interpreter began medical interpretation to the time that the clinician left the appointment.

Auxiliary time accounted for the time an interpreter spent assisting patients with accessing health care services before and after the clinical encounter without a clinician present (e.g., orienting patients to the appointment, assisting with scheduling follow up appointments, assisting with understanding medication instructions, and orienting patients to complete surveys and other patient-reported information). A benefit of having on-site interpreters is that they are available to assist with pre-visit and post-visit tasks that may enhance the care experiences of patients with LEP. Auxiliary time spent with an interpreter was rounded to 15-min intervals for billing purposes. Pre-encounter time included pre-appointment preparation, which included orienting the patient to their healthcare visit. Pre-appointment preparation did not include time that patients spent waiting without some form of interpreter-patient support. Post-encounter time included interpreter assistance with understanding medication instructions, scheduling follow-up appointments, and informing the patient about the evaluation survey to be completed on their own after their appointment. Most of the auxiliary time (75%) was post-encounter time.

The duration of interpreter time outliers was winsorized using the time values associated with the top and bottom 5th percentiles of observations. This entailed re-coding outlier time values with values at the 5th and 95th percentile values, resulting in a time duration range of 15 to 104 min.

**Figure 1.**
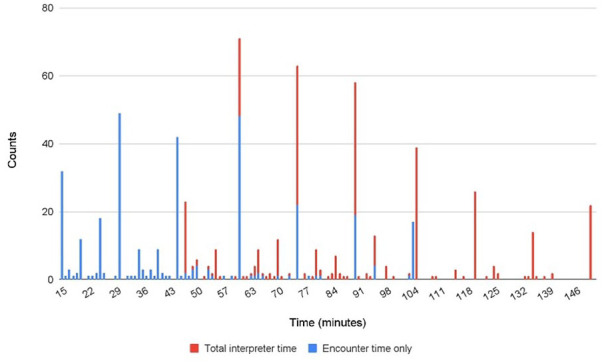
Distribution of time spent with an MIPP interpreter: encounter time only versus total interpreter time.

Time spent with an interpreter during the clinician visit had a non-normal distribution (Figure 1) and therefore needed to be log transformed. Our main independent variable is the log of patient time spent with interpreters to assess non-linear associations of patient time spent with patient care experiences.

### Outcome Measures

Patient-reported experiences of care were assessed with 2 measures: (1) clinician-patient communication and (2) interpreter support. These experiences were assessed with survey questions adapted from the visit version of the Clinician-Group version of the Consumer Assessment of Healthcare Providers and Systems (CG-CAHPS) survey.^14^ Adaptation entailed reducing language complexity so that questions were written at below a 6th grade reading-level based on Flesch–Kincaid readability tests.^15^

For clinician-patient communication, patients reported the extent to which the clinician: (1) listened carefully to them, (2) showed respect for what they had to say, (3) encouraged them to ask questions, and (4) spent enough time with them. Responses were either: “Yes definitely” (100 points), “Yes somewhat” (50 points), and “No” (0 points). Following CG-CAHPS guidance the questions were weighted equally and then averaged to create a score ranging from 0 to 100 for each patient for clinician-patient communication.^14^ The clinician-patient communication composite had adequate internal consistency reliability (α = .75).

To assess patients’ experiences of interpreter support, patients reported about 3 aspects of interpretation, which included the extent to which the interpreter: (1) helped explain how they were feeling to the clinician, (2) helped them understand the clinician's instructions, and (3) treated them with courtesy and respect. The fourth interpreter support question asked patients to report a global rating of the interpreter that ranged from 0 to 10, with 0 being the “worst interpreter possible” and 10 being the “best interpreter possible.” The global rating was rescaled by multiplying the 0 to 10 rating by 10 to enable comparable scoring ranges (0-100) with the 3 other interpreter support questions when calculating the composite measure (α = .60).

To calculate each composite measure, we followed CG-CAHPS guidance to employ the half-scale rule, which requires at least 50% of the items within the composite measure to be complete.^16^

### Control Variables

Regression models adjusted for patient age, sex, language spoken (Spanish, Haitian-Creole, “Other”), primary service provided (Primary Care, Health Education, Women’s Health, or “Other”), and visit modality (in-person vs virtual) to account for potential confounders of the relationship of duration of interpreter time and patient care experiences. For language, the “Other” language category included languages that each comprised less than 4% of the sample, which were Farsi (n = 1), Karen (n = 1), Pashto (n = 7), Russian (n = 1), and Swahili (n = 2). The regression models also controlled for the clinic service to partially account for differences in patients’ needs during the encounter. We also controlled for survey modality (phone interview vs online self-administered), self-rated physical health, and self-rated English language proficiency because past empirical studies of patient experience among LEP patients control for these covariates.^17-19^ All 27 translated surveys provided to patients are included in the Supplemental Appendix.

### Statistical Analysis

Descriptive statistics were assessed to examine the distribution of the duration of interpreter time (in min), to describe the distribution of types of visits, and to characterize the distribution of languages interpreted.

Multivariable generalized linear regression models were estimated to assess the association of the logged duration of time spent with professional medical interpreters and (1) clinician-patient communication and (2) interpreter support. We examined 4 sets of regression models: (1) Model 1 examined the encounter time that interpreters spent with patients without adjusting for control variables (2) Model 2 examined the encounter time only but adjusted for age, sex, language spoken (Spanish, Haitian-Creole, and “Other”), self-rated English proficiency, self-rated health status, reason for visit, mode of visit, and the mode of survey completion (phone vs online), (3) Model 3 examined the total time spent with interpreters, which included auxiliary time supporting patients before and after the encounter (orienting patients to the appointment, scheduling follow-up appointments, and assisting with understanding of medications) in addition to the encounter time without adjusting for covariates, and (4) Model 4 examined the total time spent with interpreters but adjusting for the same control variables as Model 2.

Finally, we conducted sensitivity analyses to examine the consistency of our results when survey respondents reporting their English language proficiency as “Very Well” or “Well.” Of the 338 respondents, 14% reported their English proficiency to be “Well” or “Very Well.” LEP patients sometimes overestimate their English proficiency or may misunderstand the survey question, leading to misclassification.

All regression models accounted for the clustering of patients within clinics using robust standard errors. We utilized a significance level of 0.05 for all statistical comparisons. CHC fixed effects were not used because the distribution of languages was highly correlated with CHC sites. All analyses were conducted using R Statistical Package: Version 2022.12.0 (2022.12.0+353).^20^

## Results

### Descriptive Analyses

The analytic sample was predominantly female (74%) and Spanish-speaking (72%; Table 1). The next most common language was Haitian Creole (24%). The other 4% of participants spoke another language, including Pashto, Farsi, Russian, Karen, and Swahili. The low volume of survey completions in languages other than Spanish reflects and is directly proportional to the low use of MIPP services in languages other than Spanish at the participating CHCs. The mean age was 38 years with a standard deviation (SD) of 18 years.

**Table 1.** Descriptive Statistics of Patient Survey Respondents (n = 338).

	Mean (standard deviation) or % of respondents
Age (mean, standard deviation)	38.3 (18.4)
Female sex	74
Patient’s preferred language	
Spanish	72
Haitian Creole	24
Other language^a^	4
Self-reported English proficiency	
Not at all	46
Not well	40
Well	11
Very well	3
Self-rated physical health	
Poor or fair	31
Good	36
Very good or excellent	33
Interpreter service mode	
In-person	57
Remote, audio-only	43
Clinical service provided	
Primary care	60
Health education	20
Women’s health	16
Other service	4
Survey administration mode	
Online, self-administered	30
By phone with a language-concordant interviewer	70
Pilot site	
CHC 1	49
CHC 2	18
CHC 3	33

a To protect human subjects, languages with less than 5% representation among respondents were combined as an “Other Language” category.

These languages included: Pashto, Farsi, Russian, Karen, and Swahili. See the Supplemental Appendix for the translated patient surveys.

On average, clinician-patient communication scores (average = 93.7/100 and SD = 14.1) were lower than interpreter support scores (average = 97.4/100 and SD = 7.0).

The average encounter duration was 47.7 min (SD = 25.1), the average auxiliary time was 43.8 min (SD = 16.4) and the average total interpreter time was 91.1 min (SD = 28.6). Although MIPP policy requires that interpreters document time spent to the minute, Figure 1 suggests that rounding occurred, as observed by the peaks at the 30-, 45-, and 60-min marks. Adding auxiliary time had a normalizing effect on the distribution of patient time spent with interpreters, with less drastic peaks.

### Adjusted Analysis

Unadjusted and adjusted results for the clinician-patient communication regression models are summarized in Table 2 and interpreter support regression models are summarized in Table 3. Clinician-patient communication (β = 2.55, 95% CI = −0.26, 5.37, *P* = .08; Table 2, Model 2) and patient experiences with interpreter support (β = −.31, 95% CI = −1.74, 1.11, *P* = .67; Table 3, Model 2) were not significantly associated with duration of patient time spent with an interpreter when examining encounter time only and adjusting for covariates.

**Table 2.** Multivariable Regression Analyses: Association of Time Spent with a Professional Medical Interpreter and Patient-Reported Experiences of Clinician-Patient Communication.

Model	Clinician-patient communication			
	Model 1: Encounter time (unadjusted)	Model 2: Encounter time (adjusted)	Model 3: Total time (unadjusted)	Model 4: Total time (adjusted)
Patients (n)	334	334	334	334
Time spent with interpreter (log of time in min)	1.21 [−1.50, 3.92]	2.55 [−0.26, 5.37]	5.88 [1.20, 10.56]*	7.23 [2.35, 12.12] **
Age		0.19 [0.11, 0.28]***		0.18 [0.10, 0.27]***
Female sex		0.54 [−3.09, 4.17]		0.54 [−3.06, 4.15]
Language				
Spanish (reference)				
Haitian Creole		4.10 [−2.56, 10.75]		4.42 [−2.17, 11.02]
Other language		1.11 [−9.25, 11.47]		0.63 [−9.62, 10.88]
English language proficiency (any vs “not at all”)		6.10 [3.14, 9.06]***		5.78 [2.84, 8.72] ***
Self-rated physical health (% excellent, very good, good)		−1.35 [−4.75, 2.04]		−1.64 [−5.01, 1.73]
In-person interpreter		−2.38 [−6.89, 2.13]		−2.94 [−7.41, 1.53]
Clinical service				
Primary care (reference)				
Health education		−1.05 [−7.41, 5.32]		−0.73 [−7.04, 5.59]
Women’s health		2.70 [−1.93, 7.33]		2.60 [−1.96, 7.16]
Other Service		1.66 [−6.34, 9.65]		1.51 [−6.42, 9.44]
Interviewer administered survey		−2.06 [−5.64, 1.52]		−3.06 [−6.65, 0.52]
Intercept	89.18 [78.99, 99.38]	75.55 [63.29, 87.81]	67.44 [46.49, 88.38]	54.50 [33.04, 75.96]
Akaike information criterion	2753.58	2698.38	2748.31	2692.99

Values are regression model point estimates, 95% confidence intervals, and *P*-values; Auxiliary time includes (1) orienting patients to the appointment, (2) assisting with scheduling follow up appointments, (3) assisting with understanding medication instructions, and (4) orienting patients to complete surveys and other patient-reported information.

* *P* < .05. **P < .01. ***P < .001.

**Table 3.** Multivariable Regression Analyses: Association of Time Spent with a Professional Medical Interpreter and Patient-Reported Experiences of Interpreter Support.

Model	Interpreter support			
	Model 1: Encounter time (unadjusted)	Model 2: Encounter time (adjusted)	Model 3: Total time (unadjusted)	Model 4: Total time (adjusted)
Patients (n)	334	334	334	334
Time spent with interpreter (log of time in min)	−0.17 [−1.51, 1.17]	−0.31 [−1.74, 1.11]	1.68 [−0.65, 4.01]	1.09 [−1.40, 3.58]
Age		0.0096 [−0.03, 0.05]		0.01 [−0.04, 0.05]
Female sex		−0.86 [−2.70, 0.98]		−0.86 [−2.70, 0.97]
Language				
Spanish (reference)				
Haitian Creole		−2.77, [−6.14, 0.60]		−2.81 [−6.17, 0.55]
Other language		−10.81 [−16.05, −5.56]***		−11.24 [−16.46, −6.01]***
English language proficiency (any vs “not at all”)		0.45 [−1.04, 1.95]		0.42 [−1.08, 1.92]
Self-rated physical health (% excellent, very good, good)		1.22 [−0.50, 2.94]		1.06 [−0.66, 2.78]
In-person interpreter		−0.69 [−2.97, 1.59]		−1.03 [−3.31, 1.24]
Clinical service				
Primary care (reference)				
Health education		−0.01 [−3.23, 3.21]		0.12 [−3.10, 3.34]
Women’s health		0.21 [−2.14, 2.55]		0.36 [−1.97, 2.68]
Other service		0.18 [−3.87, 4.22]		0.04 [−4.00, 4.08]
Interviewer administered survey		1.18 [−0.63, 2.99]		1.09 [−0.74, 2.92]
Intercept	98.03 [92.99, 103.08]	98.30 [92.10, 104.51]	89.93 [79.51, 100.35]	92.74 [81.81, 103.68]
Akaike information criterion	2278.23	2243.32	2276.29	2242.75

Values are regression model point estimates, 95% confidence intervals, and *P*-values. Auxiliary time includes (1) orienting patients to the appointment, (2) assisting with scheduling follow up appointments, (3) assisting with understanding medication instructions, and (4) orienting patients to complete surveys and other patient-reported information.

***P < .001.

**Figure 2.**
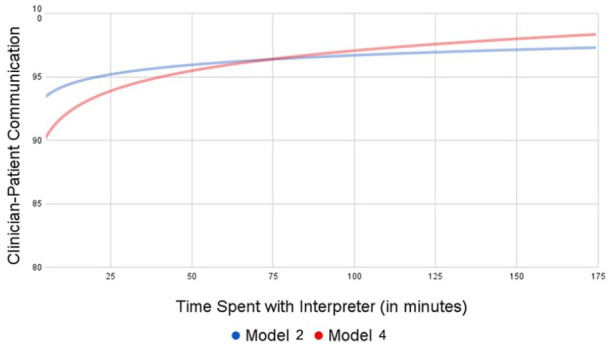
Adjusted relationship of interpreter duration and clinician-patient communication. Model 2 includes time spent with the interpreter during the clinical encounter. Model 4 includes time spent with interpreters before and after encounters in addition to time spent with the interpreter during the encounter.

However, once auxiliary time, which includes interpreter support with scheduling follow-up appointments, interpretating medication instructions, and orienting patients to patient-reported assessments was considered, more patient time spent with interpreters was significantly associated with better clinician-patient communication (β = 7.23, 95% CI = 2.35,12.12, *P* < .01; Table 2, Model 4). In other words, for every 10 additional minutes of time spent with an interpreter, clinician-patient communication scores increase by 7.23 points. The adjusted association of time spent with an interpreter and patient-reported experiences of clinician-patient communication are displayed in Figure 2. This adjusted relationship illustrates how clinician-patient communication scores change based on the duration of patient time spent with interpreters.

**Figure 3.**
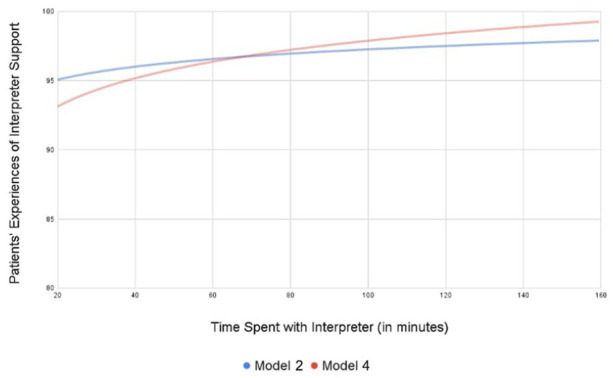
Adjusted relationship of interpreter duration and patients’ experiences of interpreter support. Model 2 includes time spent with the interpreter during the clinical encounter. Model 4 includes time spent with interpreters before and after encounters in addition to time spent with the interpreter during the encounter.

There was no association of patient time spent with interpreters and patient experiences of interpreter support (β = 1.09, 95% CI = −1.40, 3.58, *P* = 0.39; Table 3, Model 4) when patient time spent with interpreters included auxiliary time. Figure 3 displays the adjusted relationship of duration of time spent with an interpreter and patients’ experiences of interpreter support, which was not statistically significant.

There were several covariates associated with better patient care experiences (Model 4). Older age was significantly associated with better clinician-patient communication (β = .18, 95% CI = 0.10,0.27, *P* < .001). Patients reporting any level of self-rated English language proficiency were significantly associated with better clinician-patient communication and experiences of interpreter support (β = 5.78, 95% CI = 2.84, 8.72, *P* < .001). Speaking an “Other” language was associated with significantly worse experiences of interpreter support (β = −11.24 95% CI = −16.46, −6.01, *P* < .001).

As compared to Model 2 AIC scores, Model 4 has slightly lower AIC scores for both clinician and interpreter, indicating better model fit. Model 4 is considered our primary model specification because it produced more precise model estimates.

Our sensitivity analyses tested respecified both adjusted regression model using an analytic sample that excluded patients who reported “Well” or “Very Well” English-proficiency and the results were consistent with the main findings, but somewhat stronger; more patient time spent with an interpreter was associated with better clinician-patient communication in both Model 2 (β = 3.45, 95% CI = 1.04, 5.86, *P* < .01) and Model 4 (β = 7.60, 95% CI = 3.49, 11.71, *P* < .001; see Supplemental Appendix).

## Discussion

Our study, the first to assess the association of duration of time spent with medical interpreters and LEP patients’ experiences of care, found evidence of a statistically significant association between longer patient time with interpreters and better clinician-patient communication when time included interpreter support before and after clinical encounters, but not otherwise. In contrast to clinician-patient communication, longer duration of time spent with interpreters was not significantly associated with better patient experiences of interpreter support. One potential reason for this finding is that interpreter support scores were very high overall, so there was limited variation to examine in regression analyses.

Our results indicate that patients benefit from the support provided by interpreters before and after encounters to aid with resource navigation, help with understanding treatments, and to serve as cultural liaisons. These findings are consistent with past evidence of the benefits of patient time with interpreters,^4,5^ including establishing trust, increasing patient comfort, and contributing to better communication during clinical encounters. Professional medical interpretation enables patients to understand their treatment recommendations more comprehensively, which can also can improve patients’ experiences of communication with clinicians.

Although there is no large-scale empirical evidence indicating that using interpreters when language-concordant care is not possible causes harm, some are concerned that intepreters increase barriers to clinician-patient communication, especially when they are not sufficiently integrated into clinic operations.^21,22^ Our findings indicate that when language concordant care is not possible, interpreters can support high-quality clinician-patient relationships rather than impede them. Past evidence indicates that an agenda-setting intervention for physicians improved patient-reported clinician-patient communication by approximately 3 points (out of 100),^14^ highlighting practical significance of of a ~7 point effect size for 10 additional minutes with an interpreter. Ensuring adequate time with interpreters may be especially important for patients with “fair” or “poor” self-rated health, as past research indicates that they experience lower quality clinician-patient communication compared to patients with better self-rated health.^23^ More evidence is needed to understand how the duration of time spent with interpreters impacts clinician-patient communication for LEP patients with complex chronic conditions, as these subgroups may benefit most from additional time.

The study findings also highlight that CHC leadership should ensure that schedules and operational workflows allow for adequate patient time with interpreters before and after their clinical encounter. Optimizing the use of interpreters may provide CHCs the business case to expand staff interpreters for some languages rather than contract with external vendors. Policymakers should consider reimbursing for the time interpreters spend providing valuable patient navigation support beyond the clinician encounter rather than solely reimbursing for time time spent interpreting for a clinician.^24^

We also found important associations of LEP patient characteristics and patient care experiences. In adjusted analyses, LEP patients who reported speaking English “Not Well,” “Well,” or “Very Well,” reported significantly better clinician-patient communication compared to patients reporting speaking English “Not at all.” LEP patients reporting some level of English proficiency may partially understand what English-speaking clinicians are saying, which could promote better experiences of clinician-patient communication when supported by an interpreter.^25,26^ This pattern is consistent with past evidence that language concordance is associated with better patient care experiences,^27^ yet indicates lower quality communication for patients reporting no self-rated English proficiency. When the analyses excluded patients who self-reported “Well” or “Very Well” English proficiency (14% of sample population), the association of time spent with an interpreter with patient-clinician communication strengthened, suggesting that the greatest impact of interpreters on care experiences is among patients with little to no English proficiency. These results underscore the need for CHCs to ensure that LEP patients with the lowest English proficiency are prioritized as part of efforts to improve patient care experiences, including efforts to increase language concordant care and ensure responsive and culturally competent interpreter services.

Our study results should be considered with some limitations in mind. First, the 2 patient-reported study outcomes had high mean scores, which could impact the precision of estimates and are 1 reason confidence intervals for regression estimates are relatively large.^28^ Second, the survey’s response rate was modest (14%), but are consistent with other patient experience surveys conducted among underserved patient populations.^29^ Third, while most of the interpreter support questions were adapted from CAHPS, we included a question about interpreter assistance with understanding medication instructions to meet DHCS’ legislatively mandated evaluation requirements. These question modifications may contribute to the suboptimal internal consistency reliability (α = .60) for the measure, which increase measurement error and potentially bias the results toward the null hypothesis of finding no association. Fourth, patient time spent with interpreters is not measured precisely, as interpreters rounded up clinical encounter time duration to the nearest 5-min increment and pre-visit and post-visit services were documented in increments of 15 min of support. The time documented, however, reflects the time billed to the payer, which has practical relevance. Future studies should consider systematic participant observation as a method^30^ to better understand how patient time with interpreters is allocated to interpretation, patient navigation, and other support functions. Fifth, we were unable to include clinic fixed effects or the mode of survey administration in the regression analyses because these variables were collinear with the languages spoken by LEP patients, a key covariate in our analyses. Finally, the inclusion of acculturation, family support, and past experiences of discrimination could alter the regression results. Future studies should consider analyzing these factors when assessing the impact of time spent with interpreters on LEP patients’ experiences of primary care.

## Conclusion

LEP patients’ experiences of clinician-patient communication and interpreter support were overwhelmingly positive; very few problem experiences were reported. When patient time spent with the interpreter before and after the encounter was considered, our analyses revealed that more patient time spent with a professional medical interpreter was associated with significantly better clinician-patient communication. The results suggest that time spent with an interpreter beyond the encounter adds value to LEP patient experiences when language concordant care is not possible. Policymakers should consider reimbursing health care organizations for time that interpreters spend providing patient navigation and other support beyond clinical encounters.

## Supplemental Material

sj-pdf-1-jpc-10.1177_21501319241264168 – Supplemental material for Patient Time Spent With Professional Medical Interpreters and the Care Experiences of Patients With Limited English Proficiency Supplemental material, sj-pdf-1-jpc-10.1177_21501319241264168 for Patient Time Spent With Professional Medical Interpreters and the Care Experiences of Patients With Limited English Proficiency by Pamela Torresdey, Jacob Chen and Hector P. Rodriguez in Journal of Primary Care & Community Health
