# Supplementary material for: Patient Time Spent With Professional Medical Interpreters and the Care Experiences of Patients With Limited English Proficiency
Source: J Prim Care Community Health. 2024 Jun 24;15:21501319241264168. doi: 10.1177/21501319241264168 (PMC11265237; doi:10.1177/21501319241264168)
Supplement: sj-pdf-1-jpc-10.1177_21501319241264168 – Supplemental material for Patient Time Spent With Professional Medical Interpreters and the Care Experiences of Patients With Limited English Proficiency [file sj-pdf-1-jpc-10.1177_21501319241264168.pdf]

Appendix Sensitivity Analysis:
Exclusion of Participants Self-Reporting "Very Well" or "Well" English Proficiency
Multivariable Regression Analyses

	**Clinician-Patient Communication**		**Interpreter Support**	
Model	**Model 2: Encounter time (adjusted)**	**Model 4: Total time (adjusted)**	**Model 2: Encounter time (adjusted)**	**Model 4: Total time (adjusted)**
Patients (n)	**284**	**284**	**284**	**284**
Time Spent with Interpreter (log of time duration in minutes)	3.45 [1.04, 5.86]‡	7.60 [3.49, 11.71] §	-0.29 [-1.80, 1.21]	1.37 [-1.22, 3.96]
Age	-0.02 [-0.10, 0.06]	-0.03 [-0.11, 0.05]	0.00 [-0.05, 0.05]	0.00 [-0.05, 0.05]
Female Sex	-0.62 [-3.90, 2.66]	-0.79 [-4.04, 2.46]	-0.17 [-2.22, 1.88]	-0.25 [-2.29, 1.80]
**Language**				
Spanish (reference)				
Haitian Creole	-1.15 [-6.95, 4.64]	-0.90 [-6.63, 4.84]	-2.69 [-6.31, 0.94]	-2.74 [-6.35, 0.87]
Other Language	-3.63 [-12.4, 5.14]	-3.98 [-12.66, 4.70]	-11.55 [-17.03, -6.06] §	-12.08 [-17.54, -6.61] §
English Language Proficiency ("not well" vs. "not at all")	1.99 [-0.56, 4.55]	1.67 [-0.86, 4.20]	-0.26 [-1.85, 1.34]	-0.28 [-1.88, 1.32]
Self-rated Physical Health (% Excellent, Very Good, Good)	-2.29 [-5.16, 0.57]	-2.50 [-5.34, 0.34]	1.42 [-0.37, 3.21]	1.21 [-0.58, 2.99]
In Person Interpreter	-2.96 [-6.97, 1.04]	-3.46 [-7.44, 0.52]	-0.47 [-2.97, 2.03]	-0.93 [-3.43, 1.58]
**Clinical Service**				
Primary Care (reference)				
Health Education	-1.85 [-7.29, 3.59]	-1.47 [-6.87, 3.93]	-0.51 [-3.91, 2.89]	-0.35 [-3.76, 3.05]
Women's Health	1.51 [-2.42, 5.43]	1.42 [-2.44, 5.27]	-0.48 [-2.93, 1.97]	-0.24 [-2.67, 2.19]
Other Service	-3.53 [-10.48, 3.43]	-3.41 [-10.29, 3.46]	-0.27 [-4.61, 4.08]	-0.51 [-4.84, 3.82]
Interviewer Administered Survey	0.92 [-2.28, 4.12]	-0.19 [-3.39, 3.02]	0.69 [-1.31, 2.69]	0.55 [-1.47, 2.57]
Intercept	86.36 [75.82, 96.90]	67.10 [49.14, 85.06]	98.55 [91.96, 105.1]	92.03 [80.72, 103.34]
Akaike Information Criterion (AIC)	2165.83	2160.53	1898.85	1897.88

Notes" Values are regression model point estimates, 95% confidence intervals, and p-values; †p<0.05, ‡p<0.01, §p<0.001; Auxiliary time includes 1) scheduling follow-up appointments 2) interpreting medications and 3) orienting patients to complete patient-reported information in addition to time spent with patients during the encounter.

Table of Contents

English	2
Arabic	**7**
Armenian	12
Burmese	17
Chinese (Simplified)	23
Dari	28
Farsi	33
Haitian Creole	38
Hindi	43
Hmong	48
Japanese	53
Karen	59
Khmer (Cambodian)	64
Lao	70
Oromo	74
Pashto	79
Portuguese	84
Punjabi	89
Russian	93
Somali	98
Spanish	104
Swahili	109
Tagalog	114
Thai	119
Tigrinya	124
Chinese (Traditional)	129
Ukrainian	134
Vietnamese	139

English

Select the survey language:

Arabic
Armenian
Burmese
Cambodian (Central Khmer)
Chinese (Cantonese)
Chinese (Mandarin)
Dari
Farsi
Haitian Creole
Hindi
Hmong
Japanese
Karen
Korean
Laotian
Oromo
Pashto
Portuguese
Punjabi
Russian
Somali
Spanish
Swahili
Tagalog
Thai
Tigrinya
Ukrainian
Vietnamese
Other

1. Your date of birth:

2. Date of your most recent clinic appointment:

3. Did the provider **listen carefully** to you?
    a. Yes, definitely
    b. Yes, somewhat
    c. No

4. Did the provider **show respect** for what you had to say?
    a. Yes, definitely
    b. Yes, somewhat
    c. No

5. Did the provider **encourage you to ask questions**?
    a. Yes, definitely
    b. Yes, somewhat

    c. No

6. Did the provider **spend enough time** with you?
    a. Yes, definitely
    b. Yes, somewhat
    c. No

7. Did the interpreter **help you explain** how you were feeling to the provider?
    a. Yes, definitely
    b. Yes, somewhat
    c. No

8. Did the interpreter **help you understand instructions** from the provider?
    a. Yes, definitely
    b. Yes, somewhat
    c. No

9. Did the interpreter treat you with **courtesy and respect**?
    a. Yes, definitely
    b. Yes, somewhat
    c. No

10. Using any number from 0 to 10, where 0 is the worst interpreter possible and 10 is the best interpreter possible, **what number would you use to rate this interpreter**?
    a. 0 Worst interpreter possible
    b. 1
    c. 2
    d. 3
    e. 4
    f. 5
    g. 6
    h. 7
    i. 8
    j. 9
    k. 10 Best interpreter possible

11. Did the provider ask you to come back for a follow-up appointment?
    a. Yes
    b. No

12. Was an interpreter scheduled for your follow-up appointment?
    a. Yes
    b. No
    c. I don't know

13. Did the provider tell you to fill a prescription for medicine?
    a. Yes
    b. No

14. Did the interpreter **help you understand** how to take the medicine?
    a. Yes, definitely

    b. Yes, somewhat
    c. No

15. Did you know that an interpreter can translate instructions for taking prescription medicine?
    a. Yes
    b. No

16. During the past 12 months, did you have an appointment **without** an interpreter or a provider who spoke your language?
    a. Yes
    b. No

17. Who helped interpret for you when you did not have an interpreter or a provider who spoke your language? Check all that apply.
    a. Friend
    b. Family member
    c. Clinic staff

18. How did your recent appointment with the interpreter compare to your **past** appointments without an interpreter or a provider who spoke your language?
    a. Better
    b. About the same
    c. Worse

19. What number would you use to **rate your most recent experience** at the clinic? Use any number from 0 to 10, where 0 is the worst clinic experience possible and 10 is the best clinic experience possible.
    a. 0 Worst experience possible
    b. 1
    c. 2
    d. 3
    e. 4
    f. 5
    g. 6
    h. 7
    i. 8
    j. 9
    k. 10 Best experience possible

20. What is your sex?
    a. Male
    b. Female
    c. Other

21. How well do you speak English?
    a. Very well
    b. Well
    c. Not Well
    d. Not at all

22. How would you rate your overall health?

a. Excellent
b. Very Good
c. Good
d. Fair
e. Poor

Thank you for taking the time to participate in this study. We will send you a $10 electronic gift card to **your email address or to your cell phone by text** within the next 48 hours. Please indicate which method works best for you.

a) Email
b) Text

What is your email address to send the $10 electronic gift card?

What is your cell phone number to send the $10 electronic gift card?

Are you interested in discussing your recent health care experience in more detail with an interviewer? We will provide an additional **$25 electronic gift card** for your participation in a confidential 30-minute interview.

a) Yes, I am interested
b) No, I am not interested

To help plan for your upcoming interview:

What is the best **phone number** to reach you?

What is the best **email address** to reach you?

A researcher will call you within the next two weeks during a time that works for you. Please choose **all the time windows that work** with your schedule. Times are listed in Pacific Standard Time (California time).

a) Weekdays (Monday through Friday) 11AM-2PM
b) Weekdays (Monday through Friday) 3PM-6PM
c) Saturday 9AM-1PM
d) Other

If you chose “other”, **what days of the week and times** would work best for your schedule?

Thank you for your interest in participating in an interview. Please choose a date and time from **this calendar** to schedule your interview. A confirmation message will be sent to you before your interview.

Endings:

1) "The survey has ended because you did not consent to participate. If you still want to participate, please refresh your browser to restart the survey. A link to the study information sheet is included **here**."

2) "Thank you for completing the survey and for your interest in participating in an interview. Your feedback will help improve services for patients like you. Your exact interview date and time will be confirmed with you in the coming days. A link to the study information sheet is included **here**."

3) "Thank you for completing the survey. Your responses will help improve services for patients like you. A link to the study information sheet is included **here**."

Arabic

استبيان المريض

؟ما اللغة التي تتحدث بها في المنزل

العربية
األرمينية
البورمية
)الكمبودية)الخمير الوسطى
)الصينية)الكانتونية
)الصينية)الماندرين
الداري
الفارسية
كريول هاييتي
الهندية
الهمونجية
اليابانية
الكارينية
الكورية
الوية
األورومو
باشتو
البرتغالية
البنجابية
الروسية
الصومالية
اإلسبانية
السواحيلية
التاغالوغية
التايالندية
التيجرينية
األوكرانية
الفيتنامية
غير ذلك

:تاريخ ميالدك 1.

:تاريخ أحدث موعد لك في العيادة 2.

؟هل قام مقدم الخدمة باالستماع إليك بعناية 3.
نعم، بالتأكيد 1.
نعم، إلى حد ما 2.
ال 3.
؟هل أظهر مقدم الخدمة احترامه لما ذكرته 4.
نعم، بالتأكيد 1.
نعم، إلى حد ما 2.
ال 3.

؟هل شجعك مقدم الخدمة على طرح األسئلة 5.

نعم، بالتأكيد 1.
نعم، إلى حد ما 2.
ال 3.

؟هل أمضى مقدم الخدمة وقتًا كافيًا معك 6.
نعم، بالتأكيد 1.
نعم، إلى حد ما 2.
ال 3.

؟هل قام المترجم الفوري بمساعدتك في شرح كيف كنت تشعر لمقدم الخدمة 7.
نعم، بالتأكيد 1.
نعم، إلى حد ما 2.
ال 3.

؟هل قام المترجم الفوري بمساعدتك في فهم تعليمات مقدم الخدمة 8.
نعم، بالتأكيد 1.
نعم، إلى حد ما 2.
ال 3.

.هل عاملك المترجم الفوري بلطف واحترام 9.
نعم، بالتأكيد 1.
نعم، إلى حد ما 2.
ال 3.

باستخدام أي رقم من 0 إلى ،10 حيث 0 تعني أسوأ مترجم فوري ممكن و 10 تعني أفضل مترجم فوري 10.
ممكن، أي رقم ستستخدم لتقييم هذا المترجم
؟الفوري
أسوأ مترجم فوري ممكن - 0 1.
1 .2
2 .3
3 .4
4 .5
5 .6
6 .7
7 .8
8 .9
9 .10
أفضل مترجم فوري ممكن - 10 11.

؟هل طلب منك مقدم الخدمة العودة في موعد آخر للمتابعة 11.
نعم 1.
ال 2.

هل تم تحديد موعد للمترجم الفوري من أجل موعد المتابعة 12.
المحدد لك؟
.1 نعم
ال 2.
ال أعرف 3.

هل أخبرك مقدم الخدمة بأن تُكمل استمارة للحصول على وصفة طبية 13.
لدوائك؟ .1
نعم

ال 2.

؟هل قام المترجم الفوري بمساعدتك في فهم كيفية تناول الدواء14.
نعم، بالتأكيد 1.
نعم، إلى حد ما 2.
ال 3.

هل تعلم أن المترجم الفوري يستطيع ترجمة التعليمات الخاصة لتناول األدوية 15.
الموصوفة؟
.1 نعم
ال 2.

خالل الـ 12 شهًرا الماضية، هل حضرت موع ًدا بدون وجود مترجم 16.
فوري؟ .1
نعم
ال 2.

من ساعدك في َ الترجمة الفورية عندما لم يكن لديك مترجم فوري؟ ضع عالمة على كل ما 17..ينطبق

صديق 1.
فرد في 2. األسرة
طاقم العمل 3. في العيادة

ًبمواعيدك السابقة بدون مترجم فوري؟.18كيف كان موعدك األخير بحضور المترجم الفوري مقارنة
أفضل 1.
نفس الشيء تقريبًا 2.
أسوأ 3.

ما هو الرقم الذي ستستخدمه لتقييم أحدث تجربة لك في العيادة؟ استخدم أي رقم من 0 إلى ،10 حيث 0 تعني أسوأ 19.
.تجربة ممكنة في العيادات و 10 تعني أفضل تجربة ممكنة في العيادات
أسوأ تجربة ممكنة - 0 1.
1 .2
2 .3
3 .4
4 .5
5 .6
6 .7
7 .8
8 .9
9 .10
أفضل تجربة ممكنة - 10 11.

؟ما هو نوعك20.
ذكر 1.
أنثى 2.
غير ذلك 3.

؟ما مدى إتقانك للتحدث بالغة اإلنجليزية21.
جيد للغاية 1.
جيد 2.
ال أتقنها بشكل جيد 3.
ًا.4 ال أتقنها اطالق

؟كيف تقيّم صحتك بشكل عام22.
ممتازة 1.
جيدة جدًا 2.
جيدة 3.
مقبولة 4.
ضعيفة 5.

نشكرك على تخصيص بعض من وقتك للمشاركة في هذه الدراسة. سنرسل لك بطاقة هدية إلكترونية بقيمة 10 دوالرات على عنوان بريدك اإللكتروني أو إلى هاتفك النقال عبر رسالة نصية خالل الـ 48 ساعة القادمة. يرجى اإلشارة إلى أي الطرق تناسبك أكثر.
البريد اإللكتروني 1.
رسالة نصية 2.

.ما هو عنوان بريدك اإللكتروني حتى نرسل لك بطاقة الهدية اإللكترونية بقيمة 10 دوالر

.ما هو رقم هاتفك النقال حتى نرسل لك بطاقة الهدية اإللكترونية بقيمة 10 دوالر

هل أنت مهتم بمناقشة تجربتك األخيرة في تلقي الرعاية الصحية بمزيد من التفاصيل مع محاور؟ سنقدم بطاقة هدية إلكترونية إضافية بقيمة 25 دوالر لمشاركتك في مقابلة سرية مدتها 30 دقيقة.
نعم، أنا مهتم 1.
ال، لست مهتًما 2.

:للمساعدة في التخطيط لمقابلتك القادمة

؟ما هو أنسب رقم هاتف للتواصل معك
؟ما هو أنسب عنوان بريد إلكتروني للتواصل معك

سيقوم أحد الباحثين باالتصال بك في خالل األسبوعين القادمين في الوقت الذي يناسبك. يرجى اختيار جميع المواعيد الزمنية التي تتوافق مع جدولك. األوقات ُمدرجة بتوقيت المحيط الهادي القياسي)توقيت كاليفورنيا(.
أيام األسبوع)من االثنين إلى الجمعة(11 قبل الظهر - 2 عصًرا 1.
أيام األسبوع)من االثنين إلى الجمعة(3 عصًرا - 6 مساًء 2.
السبت 9 صباًحا - 1 بعد الظهر 3.
غير ذلك 4.

؟إذا اخترت "غير ذلك"، فما هي أيام األسبوع واألوقات التي تناسب جدولك الزمني بالشكل األفضل

نشكرك على اهتمامك في المشاركة في مقابلة. يرجى اختيار تاريخ ووقت من جدول المواعيد هذا لتحديد موعد مقابلتك.
.*سيتم إرسال رسالة تأكيد إليك قبل موعد المقابلة

:النهايات
انتهى االستبيان ألنك لم توافق على المشاركة. إذا كنت ال تزال ترغب في المشاركة، يرجى تحديث المتصفح الخاص بك ")1 إلعادة بدء االستبيان. تم إدراج رابط لورقة معلومات الدراسة هنا".

2) نشكرك على إكمال االستبيان وعلى اهتمامك في المشاركة في مقابلة. سيسهم رأيك في تحسين الخدمات المقدمة لمرضى في مثل حالتك. سيتم التأكيد معك في األيام القادمة على تاريخ ووقت مقابلتك بالضبط. تم إدراج رابط لورقة معلومات الدراسة "هنا".

3) نشكرك على إكمال االستبيان. ستسهم ردودك في تحسين الخدمات المقدمة لمرضى في مثل حالتك. تم إدراج رابط لورقة "معلومات الدراسة هنا".

Armenian

Հիվանդի հարցում

Ո՞ր **լեզվով** եք խոսում տանը:

Արաբերեն
Հայերեն
Բուրմերեն
Կամբոջերեն (Կենտրոնական խմեր)
Չինարեն (Կանտոներեն)
Չինարեն (Մանդարին)
Դարի
Ֆարսի
Հաիտիան կրեոլ
Հինդի
Հմոնգ
Ճապոներեն
Կարեն
Կորեերեն
Լաոսերեն
Օռոմո
Պասհտո
Պորտուգալերեն
Փունջաբի
Ռուսերեն
Սոմալի
Իսպաներեն
Սվահիլի
Տագալոգ
Թայերեն
Տիգրինյա
Ուկրաիներեն
Վիետնամերեն
Այլ

1. Ձեր ծննդյան ամսաթիվը՝

2. Կլինիկայում ձեր ամենավերջին ժամադրության օրը՝

3. Ձեր մատակարարը ձեզ **ուշադրությամբ լսե՞ց**:
    a. Այո, միանշանակ
    b. Այո, որոշ չափով
    c. Ոչ

4. Մատակարարը **հարգանք ցուցաբերե՞ց** ձեր ասելիքի նկատմամբ:
    a. Այո, միանշանակ
    b. Այո, որոշ չափով
    c. Ոչ

5. Մատակարարը **խրախուսե՞ց ձեզ հարցեր տալ**:
    a. Այո, միանշանակ
    b. Այո, որոշ չափով

c. Ոչ

6. Մատակարարը **բավական ժամանակ անցկացրե՞ց** ձեզ հետ:
   a. Այո, միանշանակ
   b. Այո, որոշ չափով
   c. Ոչ

7. Թարգմանիչն **օգնե՞ց ձեզ բացատրել** մատակարարին, թե ինչպես եք ձեզ զգում:
   a. Այո, միանշանակ
   b. Այո, որոշ չափով
   c. Ոչ

8. Թարգմանիչն **օգնե՞ց ձեզ հասկանալ մատակարարի հրահանգները:** a. Այո, միանշանակ
   b. Այո, որոշ չափով
   c. Ոչ

9. Թարգմանիչը ձեզ հետ **քաղաքավարությամբ և հարգանքով** վերաբերվե՞ց: a. Այո, միանշանակ
   b. Այո, որոշ չափով
   c. Ոչ

10. Օգտագործելով 0-ից 10 համարները, որտեղ 0-ն՝ հնարավոր ամենավատ թարգմանիչն է և 10-ը՝ հնարավոր ամենալավ թարգմանիչը, **ո՞ր թիվը կօգտագործեիք այս թարգմանչին գնահատելու համար**:
    a. 0 Հնարավոր ամենավատ թարգմանիչը
    b. 1
    c. 2
    d. 3
    e. 4
    f. 5
    g. 6
    h. 7
    i. 8
    j. 9
    k. 10 Հնարավոր ամենալավ թարգմանիչը

11. Մատակարարը խնդրե՞ց ձեզ վերադառնալ հեռբուժական այցելության:
    a. Այո
    b. Ոչ

12. Թարգմանչի ժամադրություն նշանակվե՞ց է ձեր հետբուժական այցելության համար: a. Այո
    b. Ոչ
    c. Չգիտեմ

13. Մատակարարը ձեզ ասե՞լ է դեղի դեղատոմս լրացնել:
    a. Այո
    b. Ոչ

14. Թարգմանիչն **օգնե՞ց ձեզ հասկանալ,** թե ինչպես ընդունել դեղը: a. Այո, իհարկե
    b. Այո, որոշ չափով
    c. Ոչ

15. Դուք գիտեի՞ք, որ թարգմանիչը կարող է թարգմանել դեղատոմսով դեղերի ընդունման հրահանգները:
    a. Այո
    b. Ոչ

16. Վերջին 12 ամսվա ընթացքում **առանց** թարգմանչի ժամադրություն ունեցե՞լ եք: a. Այո
    b. Ոչ

17. Ո՞վ օգնեց թարգմանել ձեզ համար, երբ թարգմանիչ չունեիք: Նշեք բոլոր կիրառելիները:
    a. Ընկեր
    b. Ընտանիքի անդամ
    c. Կլինիկայի անձնակազմ

18. Ինչպե՞ս կհամեմատեիք թարգմանչով ձեր վերջին ժամադրությունը առանց թարգմանչի ձեր **նախորդ** ժամադրությունների հետ:
    a. Ավելի լավ
    b. Գրեթե նույնը
    c. Ավելի վատ

19. Ի՞նչ համար կօգտագործեիք՝ **գնահատելու ձեր ամենավերջին փորձառությունը** կլինիկայում: Օգտագործեք 0-ից 10-ը ցանկացած համար, որտեղ 0-ն՝ կլինիկայում հնարավոր ամենավատ փորձառություն է և 10-ը՝ կլինիկայում հնարավոր ամենալավ փորձառություն է:
    a. 0 Հնարավոր ամենավատ փորձառություն
    b. 1
    c. 2
    d. 3
    e. 4
    f. 5
    g. 6
    h. 7
    i. 8
    j. 9
    k. 10 Հնարավոր ամենալավ փորձառություն

20. Ո՞րն է ձեր սեռը:
    a. Արական
    b. Իգական
    c. Այլ

21. Որքա՞ն լավ եք խոսում անգլերեն:
    a. Շատ լավ
    b. Լավ
    c. Ոչ այնքան լավ
    d. Չեմ խոսում

22. Ինչպե՞ս կգնահատեիք ձեր ընդհանուր առողջությունը:
    a. Հիանալի
    b. Շատ լավ
    c. Լավ
    d. Բավարար
    e. Վատ

Շնորհակալություն այս ուսումնասիրությանը մասնակցելու նպատակով ժամանակ հատկացնելու համար: Առաջիկա 48 ժամվա ընթացքում մենք ձեզ $10 էլեկտրոնային նվեր քարտ կուղարկենք **ձեր էլ. հասցեին կամ բջջային հեռախոսին**: Խնդրում ենք նշել, թե որ մեթոդն է լավագույնս աշխատում ձեզ համար:

a) Էլ.հասցե
b) Տեքստ

Ո՞րն է ձեր էլ. հասցեն $10 էլեկտրոնային նվեր-քարտ ուղարկելու համար: Ո՞րն է ձեր

բջջային հեռախոսահամարը $10 էլեկտրոնային նվեր քարտ ուղարկելու համար:

Հետաքրքրվա՞ծ եք հարցազրույց վարողի հետ ավելի մանրամասն քննարկել ձեր վերջին առողջապահական փորձառությունը: Մենք կտրամադրենք լրացուցիչ **$25 էլեկտրոնային նվեր քարտ**՝ գաղտնի 30 րոպեանոց հարցազրույցին ձեր մասնակցության համար: a) Այո, ես հետաքրքրված եմ

b) Ոչ, ես հետաքրքրված չեմ

Օգնելու համար պլանավորել ձեր առաջիկա հարցազրույցը.

Ո՞րն է ձեզ հետ կապվելու ձեր **հեռախոսահամարը**:

Ո՞րն է ձեզ հետ կապվելու ձեր **Էլ.հասցեն**:

Հետազոտողը կզանգահարի ձեզ մոտակա երկու շաբաթվա ընթացքում **ձեզ համար հարմար ժամանակահատվածում**: Խնդրում ենք ընտրել **բոլոր ժամանակահատվածները**, որոնք աշխատում են ձեր ժամանակացույցի հետ: Ժամանակները նշված են Խաղաղ օվկիանոսի ստանդարտ ժամանակով (Կալիֆորնիայի ժամանակով):

a) Աշխատանքային օրերին (Երկուշաբթիից ուրբաթ) 11AM-2PM
b) Աշխատանքային օրերին (Երկուշաբթիից ուրբաթ) 3PM-6PM
c) Շաբաթ օր 9AM-1PM
d) Այլ

Եթե ընտրեցիք «այլ», շաբաթվա ո՞ր օրերն ու ժամերն են ձեզ հարմար: Շնորհակալություն հարցազրույցին մասնակցելու հետաքրքրության համար: Խնդրում ենք **այս օրացույցից** ընտրել ամսաթիվ և ժամ՝ ձեր հարցազրույցը պլանավորելու համար: Նախքան հարցազրույցը ձեզ կուղարկվի հաստատման հաղորդագրություն:

Ավարտը.

1) «Հարցումն ավարտվել է, քանի որ դուք համաձայնություն չեք տվել մասնակցել: Եթե դեռ ցանկանում եք մասնակցել, խնդրում ենք թարմացնել ձեր դիտարկիչը՝ հարցումը վերսկսելու համար: Ուսումնասիրության տեղեկատվական թերթիկի հղումը ներառված է **այստեղ**:»

2) «Շնորհակալություն հարցումը լրացնելու և հարցազրույցին մասնակցելու ձեր հետաքրքրության համար: Ձեր կարծիքը կօգնի բարելավել ծառայությունները ձեզ նման հիվանդների համար: Ձեր հարցազրույցի կոնտկրետ ամսաթիվը և ժամը կհաստատվեն ձեզ հետ առաջիկա օրերին: Ուսումնասիրության տեղեկատվական թերթիկի հղումը ներառված է **այստեղ**:»

3) «Շնորհակալություն հարցումը լրացնելու համար: Ձեր պատասխանները կօգնեն բարելավել ծառայությունները ձեզ նման հիվանդների համար: Ուսումնասիրության տեղեկատվական թերթիկի հղումը ներառված է **այստեղ**:»

Burmese

လူနာက ို စစ်တမ်းကကာက်ယူမှု

သင်အေ့ ိမ်တွင်မည်သည့်**ဘာသာစကား**ကို ပ ပြောဆိုပါသလဲ။

အြောပေဗျ

အြောမန်းန ်းေန်း

မန်မြော

ကပမဘြောဒ ်းယြော်း (စင်ထရယ်ခမာ)

တရုတ် (ကန်တုန်း)

တရုတ် (မန်ဒရင်း)

ဒါေ

ဖြောဆ

ဟိုင်တန် ခေ ရို ်း

ဟိန္ဒူ

မုိန်

ဂျပန်

ကေင်

ကိုိေ ်းယြော်း

လြောအို

အိုရုမို

ပတ်ရ ်တို

ပပေါ်တူဂ

ပန်ချြောပ

ရုရ ြ ေ ာ ် း

ဆိုမြောလ

စပိန်

ဆွြ ေ ာဟ လ

တြောဂါပလျော်ွ

ထိုင်း

တိုင်ဂေင်ြေော

ယူကေိန်း

ဗ ယက်နမ်

အ ခြော်း

1. သင့်ပ်မွ်းသကကေြေောဇ်

2. သင်ပနြောက်ဆ ိုဝ်း ပဆ်းခန်ေက်ချိန် ရ ိသည့် ေက်စွ -

3. သင်ကဲ့ိိုပစြောင်ပ့်ရ ြေောက်ပပ်းသူသည်သင်စ့်ကြော်းကို**ကသခ ာစ ာ နာ်းက ာင်ပါ**သလြော်း။

a. ဟိုတ်၊ လ ိုဝ်းဝပသခြော

b. ဟိုတ်၊ အတိုင်းအတြော တစ်ခိုအထိ

c. မဟိုတ်

4. သင်ကဲ့ိိုပစြောင်ပ့်ရ ြေောက်ပပ်းသူသည်သင်ပ် ပြောဆိုသိ ည်မျြော်းကို**တိကလ်းတစာ်းရ က ကာင််**

ပပါသလြော်း။ a. ဟိုတ်၊ လ ိုဝ်းဝပသခြော

b. ဟိုတ်၊ အတိုင်းအတြော တစ်ခိုအထိ

c. မဟိုတ်

5. **သငက ် ိုကစာငက ်ရ ာကက**
်ပ်းသသူ ည်သငက ် ိုကမ််းခ န််မ ာ်းပမ်း မန််ေန် အြော်းပပ်းပါသလြော်း။

a. ဟိုတ်၊ လ ိုဝ်းဝပသခြော

b. ဟိုတ်၊ အတိုင်းအတြော တစ်ခိုအထိ

c. မဟိုတ်

6. သင်ကဲ့ိိုပစြောင်ပ့်ရ ြေောက်ပပ်းသူသည်**သငက ် ိုအခ န်အလ ိုအကလာကက**
်ပ်းပါသလာ်း။

a. ဟိုတ်၊ လ ိုဝ်းဝပသခြော

b. ဟိုတ်၊ အတိုင်းအတြော တစ်ခိုအထိ

c. မဟိုတ်

7. စကြော်း ပန်က **သင်ဘယ်လ ိုခ စ်ားကနရသလဲမ ာ်းက ို**သငက်ေ့်ိဝိုပစြောငပ့်ရ ြေောက်ပပ်းသူကိုရိ င်း်

ပေြေောတွင်ကူည ပါသလြော်း။ a. ဟိုတ်၊ လ ိုဝ်းဝပသချြော

b. ဟိုတ်၊ အတိုင််းအတြော တစ်ခိုအထိ

c. မဟိုတ်

8. စကြော်း ပန်က **သငက ် ိုကစာငက ်ရ ာကက**

်ပ်းသ၏ူ ညွှန် ကာ်းခ ကမ

် **ာ််းက ို**သင်ခြော်းလည်ပအြောင် ကူည ပါသလြော်း။

a. ဟိုတ်၊ လ ိုဝ်းဝပသချြော

b. ဟိုတ်၊ အတိုင််းအတြော တစ်ခိုအထိ

c. မဟိုတ်

9. စကြော်း ပန်က သင်ကို**တကလ်းတစ်း ဆက်ဆ ပါသလာ်း**။

a. ဟိုတ်၊ လ ိုဝ်းဝပသချြော

b. ဟိုတ်၊ အတိုင််းအတြော တစ်ခိုအထိ

c. မဟိုတ်

10. န ပါတ် 0 မှ10 ရှုပိပ ်း 0 သည်အဆိုး်ဆ ု်း စကာ်းပ န်ပြစ်ပ ်း 10 သည်အကကာင််းဆ ု်း စကာ်းပ န်ပြစ်လျှ င် ဤစကြော်း ပနက် ိုဝ်အဆငသ့်တ်မ တော်ြေောတွင်သင်မည်သည့် န ပါတ်ကိုသ ိုဝ်းမလဲ။

a. 0 သည်အဆိုး်ဆ ု်း စကာ်းပ န်ပြစ်သည်

b. 1

c. 2

d. 3

e. 4

f. 5

g. 6

h. 7

i. 8

j. 9

k. 10 သည်အကကာင််းဆ ု်း စကာ်းပ န်ပြစ်သည်

11. သငက်ေ့်ိဝိုဝနပ် ဆြောငမ် ှုပပ်းသူက ပနြောက်ထပေ်က်ချိန််း ပနလ် ြေော ပနေ်

န်ချိန််းဆိုိပါသလြော်း။ a. ဟိုတ်

b. မဟုတ်

12. စကြော်း ပန်က သင်၏ ပနြောက်ထပေ်က်ချိန်းကိုပြန်ချိန်းဆိုပ်ပ်းပါသလျော်း။

a. ဟုတ်

b. မဟုတ်

c. မသိပါ

13. သင်ကဲ့ိိုဝနပ် ဆောင်မ် ှုပပ်းသူက ပဆ်းဝါးအတွက်ပဆ်းညွှန်း်စြောတစပ် စြောင်က် ိို ဖည့််စွက်ကေ်နပ်

ပြောပါသလျော်း။ a. ဟုတ်

b. မဟုတ်

14. စကြော်း ပန်က ပဆ်းကိုဘယ်လိုပိသြောက်မေလဲ**သင်နာ်းလည်ကအောင်** ကူည ပပ်းပါသလျော်း။

a. ဟုတ်၊ လ ိုစ်းဝပသချြော

b. ဟုတ်၊ အတိုင််းအတြော တစ်ခိုအထိ

c. မဟုတ်

15. စကြော်း ပန်တစ်ပယြောက်သည် ညွှန်ကြော်းလိုက််သည့် ပဆ်းကိုဘယ်လိုပိသြောက်မေလဲ

လမ််းညွှန်ချက်ကို ဘြောသြော ပန်ပပ်းနိုိုင်တယ်ဆိုိဘြော သင်သိပါသလျော်း။

a. ဟုတ်

b. မဟုတ်

16. လွှနခ် ဲ်ပြော 12 လအတွင််းက သင်သည်စကာ်းပ န်တစ်ကယာက်**မပါဘဲ**

ပသခဲ်ဖူ်းပါသလျော်း။ a. ဟုတ်

b. မဟုတ်

17. စကြော်း ပန်မရ ိချိန်တွင်ဘယ်သူက သင်ကဲ့ိိုကူည စကြော်း ပနပ် ပ်းပါသလဲ။ သက်ဆိုင်သ်

ည်မြော်းအြော်းလ ိုစ်းကိုပေွ်းချယ်ပါ

a. သူငယ်ချင််း

b. မိသြော်းစိုဝင်

c. ပဆ်းခန််းဝန်ထမ််း

18. စကြော်း ပနရ် ိပပ ်ား ပဆ်းခန်း် ပေ ခင်း် နှ ်ံ့စကြော်း ပနမ် ရ ိဘဲပဆ်းခန်း် ပေ ခင်း် အပတွေ့အကက

ပြုံကိုနိုင်ု ်း်ယ ဉ်ကကည်ပ့် ါက **သင်**ဘယ်လိုထိင်သလဲ။

a. ပိုပိကြောင််ပါသည်

b. တူတူပါဘဲ

c. ပိုဆိုး်ားပါသည်

19. ပဆ်းခန်း်တွင်သင်ပ့်ခြောက်ဆိုင််း အပတွေ့အကက ပြုံကိုအဆင်သ့််တ်မ တော်ပြေောတွင်**သင်မည်သည ်** န

ပါတ်ကို်သ ိုဝ််းမလဲ။ န ပါတ် 0 မှ10 ရှုိပ ်း 0 သည်အဆိုးဆ ု််း ကဆ်းခန််းအကတွေ့အကက

ပြုံပြစ်ပ ်း 10 သည်အကကောင််းဆ ု််း ကဆ်းခန််းအကတွေ့အကက ပြုံ ပြစ်လျှင််ဤစကား်းပ

န်ကိုအဆင့်သတ်မ တော်ပြေောတွင် သင်မ ့ ည်သည့် န ပါတ်ကို်သ ိုဝ််းမလဲ။

a. 0 အဆိုးဆ ု််း ကဆ်းခန််းအကတွေ့အကက ပြုံ

b. 1

c. 2

d. 3

e. 4

f. 5

g. 6

h. 7

i. 8

j. 9

k. 10 အကကောင််းဆ ု််း ကဆ်းခန််းအကတွေ့အကက ပြုံ

20. သင်သည် အမျိုပြုံ််းသော်းလော်း အမျိုပြုံ််းသမ ်းလော်း

a. အမျိုပြုံ််းသော်း

b. အမျိုပြုံ််းသမ ််း

c. အ ခြော်း

21. သင် အဂဂလိပ်စြော ဘယ်ပလောက် ပကြောင််ပကြောင်် ပ ပြောတတ်ပါသလဲ။

a. အလွန်ပကြောင််စွပြေော

b. ပကြောင််ပကြောင််

c. သိပ်မပကြောင််

d. လ ိုဝ််းဝမပ ပြောတတ်

22. သင့်ကျန်း်မြောပေ်းအပ ခအပနကိုသိငဘ် ယ်လိုသိတ်မ တ်မလဲ။

a. အလွန်ပကြောင်း်

b. ပတြော်ပတြော်ပကြောင်း်

c. ပကြောင်း်

d. အသငအ့် ်တင့််

e. ည ်ု့

ဤပလ့်ပြောမှုတွင် ပါဝင်ပပ်းသည့်အတွက် ပကျ်းဇူ်းတင်ပါသည်။ ပနြောက်ထပ် 48 နာရ အတွင်း် သင့်**အ ်းကမ်းလ်လ ပ်စာ သမ**

ိုု ဟိုတ်သငဖ ် ိုုန််န ပါတ
်သ ဲ ိုုိ$်ု့ 10 အ လက်ထကရာနစ်လက်ကဆာင်ကဒ်ပ ာ််း တစ်ခု ုိုုက့ ််း ါမည်။

သငအ့် တွက် မည်သည့်နည််းလမ််းသည်အကကာင််းဆ ု််းလဲဆိုတာ ကြာ်ပ က ််း ါ။

a) အ ််းပမ််းလ်

b) မက်ပဆ့်ချ်

သင၏် မည်သည့််အ််း ပမ််းလ်လိပ်ြောသိုိ$်ု့ 10 အ လက်ထကရာနစ်လက်ကဆာင်ကဒ်ပ ာ််းကို ုိုုက့

််းရမည်လဲ။ သင၏် မည်သည့််ဆလဲ ်ဖိုန််း်နပ ါတ်သိုိ$်ု့ 10 အ လက်ထကရာနစ်လက်ကဆာင်ကဒ်ပ

ာ််းကို ုိုုက့ ််းရမည်လဲ။

သငသ် ည်သင၏် လတ်တပပြော ကျန််း်မြောပေ်း ပဈောင့််ရ ပြေ ောက်မှုဆိုိငေ် ပြေ ော အပတွ့ေ့အကက ပြေုံ့မျြော်းကိုပိမ််း မန််းသူတစပ် ယြောက်နှ ််ု့ ပဆွ်းပနွ််းေ် စိတ်ဝင်ဈော်းပါသလြော်း။ လျှိြုဝုံ့ွေ့ ဝ က် 30-မိုနစ်အင်တာဗျ ််းတစ်ခုတွင် ါဝင် ုါက ပနြောက်ထပ် **$25 အီလက်ထရ ောနစ်လက်ရ ောင်ကဒ်ပ ောြော်း** တစ်ခိုကိုထပ်ပပ်းပါမည်။

a) ဟိုတ်၊ ကျွန်ုိုပ်စိတ်ဝင်ဈော်းပါသည်

b) မဟိုတ်၊ ကျွန်ုိုပ်စိတ်မဝင်ဈော်းပါ

သငန္ ် င ််ႇပနေ်ာငတ် ႏွင်အငတ် ပြေ ောဇဗျူ ်းေန်စ စဥ္ေန်အတြက်

သငကဲ့ ိိုဆက်သြယ်ႏိုုိင် ည္ဲ့အဆငအ် ပ ပဆို် ်း **ဖုန္း်န ပါတ်**က ေဘြာလဲ။

သငကဲ့ ိိုဆက်သြယ်ႏိုုိင် ည္ဲ့အဆငအ် ပ ပဆို် ်း **အ ် ်းကမ်းလ်လ ပ်စာ** က ေဘြာလဲ။

နွ ် ပတ်အတြင်း် သငအ္ဲ့ ်တြက်အဆငပ် ပသည္ဲ့အခ်ိနတ် ႏွင္သိုပတသနပဘြာရ ငတ် စပ် ေယြာက်က သငထ္
ဖုန္း်ဆက်ပါလိမ္ဲ့မည္။ သငအ္ဲ့ ်ခ်ိန္ဇ ေယြာ်းႏွ ကဲ့ ိိုက်ည မည္ဲ့ **အခ န်က က်မ ာ ် ်းအာ်းလ ိုံ ်းက** ိုပေ ြ ်းခ်ယ်ပပ်းပါ။
အခ်ိန္မ်ြာ်းသည် ပစိဖိတ် စ ပေတြာ်ခ်ိန် (ကယ်လ ႏြ ို ်းန ််းယာ်းအခ်ိန်) ပြင္ဲ့သတ်မွတ်ထာ်း ါသည္။

a) ရို ််းဖွငေ်ဲ့ ် က်(တနလလာကန ့မွကသာကကာကနထ့) ႏိုကနလ့ ည်11-ညကန2နာရ
b) ရို ််းဖွငေ်ဲ့ ် က်(တနလလာကန ့မွကသာကကာကနထ့) ႏိုညကန3-ညကန6နာရ
c) စပနပန မနက်9 ် ့ -ကနလ့ ည်1နာရ
d) အ ခြော်း

 သင်သည် "အပဓာ်း"ကိုုကရွ်း ါက **မည်သည ်ရက် မည်သည ်အခ န်သည်**သငအ္ဲ့ ်တြက်အဆငအ် ပ ပဆို် ်း ဖစ်ပါသနည််း။

အငတ် ပြေ ောဇဗျူ ်းတြင်ပါဝငေ် န်စတိ ်ဝငစ် ပြေ ော ််းသည္ဲ့အတြက်ပကျ်းဇူ ််းတငပ် ါသည္။ သငအ္ဲ့ ်ငတ်
ပြေ ောဇဗျူ ်းအတြက်ေက်ချိန္း်ယူေန် **ဤပပကခဒ န် ဲမ** ေက်နွ ််ႇအခ်ိနတ် စခ် ိုကိုပေ ြ ်းခ်ယ်ပပ်းပါ။ အငတ်
ပြေ ောဇဗျူ ်းမလိုပ်မ သငထဲ့ ်သ ိုပိုဲ့
အတည် ပြိုမက်ပဆခ္ဲ့ ႏွတ် စပ် ေဘြာင်ပိုဝှ်ပ်းပါမည္။

အဆ ို ်းသတ် ခင်းမြော်း
1)"သငသ် ည်ပါဝငေ် န်သပဘြာမတူသ ဖင္ဲ့စ်စတ် မ်းကိုအိဆို် ်း သတ်လိုက်ပါသည္။ သငပ် ါဝငလ် ိုပသ်းပါကစစတ် မ်းကို
ပနစ် တငေ် န်သဘင္ဲ့ ်ပေပြေ ောက်ဖြောကိုေ ဖေက်ရ ်လိုပ်ပါ။ ပလလ္ဲ့ ပြေ ောမှုအချက်အလက်စြောေ ြက်ထ လင်ခ္ဲ့ ်တစခ်
ို**ဤ**တြင် ပါပါသည္။
2)"စစ်တမ်းတြင် ါဝင်က ််းပ ််း အင်တာဗျ ််းတြင် ါဝင်ရန်စိတ်ဝင်စာ်းသည့်အတွက် ပကျ်းဇူ ််းတင်ပါသည်။ သငတ်ဲ့ ို ပ့် နခ်
ပျက်မြော်းသည်သငက် ဲသ့် ိုပိုဲ့သေဘြာ လူနေ်ာမြော်းကိုဝိနပ် ဆြောငမ် ႏိုပိုကြောင်း်ပအြောင်ကူည ႏိုုပ် ါသည္။
မကကြောမ သငနေ္ ် င ််ႇ အငတ် ပြေ ောဇဗျူ ်းမည္ဲ့ေ်က်နွ ််ႇအခ်ိနက် ိုအတည် ပြိုပပ်းပါလိမ္ဲ့မည္။ ပလလ္ဲ့
ပြေ ောမှုအချက်အလက်စြောေ ြက်ထ လင်ခ္ဲ့ ်တစခ် ို**ဤ**တြင် ပါပါသည္။

3)"စစ်တမ်းကကာက်ယူမှုကိုဖြည့်စွ် ွက်ပပ်းသည်အ့် တွက်ပကျ်းဇူ်းတငပ် ါသည်။ သင့်အပ ဖမြေျ်ာ်းသည်သငက် ဲသ့်

ိုပိ်ုဘြော လူနြေ်ာမြေျ်ာ်းကိုဝိနပ် ဆြော်ငမ် ှုပိုပိကြောင်း်ပအြောင်ကူည နိုင်ပ် ါသည်။ ပလလ့်

ပြြေောမှုအချက်အလက်စြေ်ာေွက်ထ လငခ့်််တစခ် ို**ဤ**တွင် ပါပါသည်။

Chinese (Simplified)

患者调查

您在家里讲什么语言？

阿拉伯语

亚美尼亚语

缅甸语

柬埔寨语（中高棉）

中文（粤语）

中文（普通话）

达里语

波斯语

海地克里奥尔语

印地语

苗语

日语

凯伦语

韩语

老挝语

奥罗莫语

普什图语

葡萄牙语

旁遮普语

俄语

索马里语

西班牙语

斯瓦希里语

他加禄语

泰语

提格里尼亚语

乌克兰语

越南语

其他

1. 您的出生日期：

2. 您最近一次门诊看诊日期：

3. 医务人员是否仔细倾听了您的意见？ a.
   是，当然
   b. 是，有点
   c. 否

4. 医务人员是否尊重您说的话？
   a. 是，当然
   b. 是，有点
   c. 否

5. 医务人员是否鼓励您提问？
   a. 是，当然
   b. 是，有点
   c. 否

6. 医务人员是否为您花了足够时间？
   a. 是，当然
   b. 是，有点
   c. 否

7. 口译员是否帮您解释了您的感受给医务人员听？
   a. 是，当然
   b. 是，有点
   c. 否

8. 口译员是否帮您理解了医务人员的说明？
   a. 是，当然
   b. 是，有点

c. 否

9. 口译员对待您是否有礼貌和尊重？

a. 是，当然

b. 是，有点

c. 否

10.用 0 到 10 之间的任何数字，0 表示最差，10 表示最佳，您会用什么数字来评价 这个口译员？

a. 0 表示最差口译员

b. 1

c. 2

d. 3

e. 4

f. 5

g. 6

h. 7

i. 8

j. 9

k. 10 表示最佳口译员

11.医务人员是否要求您返回复诊？

a. 是

b. 否

12.是否为您的复诊安排了口译员？

a. 是

b. 否

c. 不知道

13.医务人员是否告诉您去拿处方药？

a. 是

b. 否

14.口译员是否帮您明白了如何服药？

a. 是，当然

b. 是，有点

c. 否

15.您是否知道口译员可以翻译服用处方药说明？ a.

是

b. 否

16.在过去的 12 个月中，您是否有过没有口译员的看诊？ a.

是

b. 否

17.没有口译员时，谁帮您翻译？勾选所有适用项。 a.

朋友

b. 家人

c. 诊所工作人员

18.您最近有口译员的看诊与过去没有口译员的看诊相比如何？ a.

更好

b. 差不多

c. 更差

19.您会用什么数字来评价您最近的诊所体验？用 0 到 10 之间的任何数字，0 表示最 差 ，10 表示最佳。

a. 0 表示最差体验

b. 1
c. 2
d. 3
e. 4
f. 5
g. 6
h. 7
i. 8
j. 9

k. 10 表示最佳体验

20.您的性别？

a. 男

b. 女

c. 其他

21.您的英语说得怎么样？

a. 很好

b. 好

c. 不好

d. 一点也不会

22.您如何评价您的整体健康状况？

a. 极佳

b. 很好

c. 好

d. 不错

e. 很差

感谢您抽出宝贵时间参与这项研究。在接下来的 48 小时内，会将一张 10 美元电子礼品 卡发送到您的电子邮件地址或通过短信发送到您的手机。请说明您喜欢使用哪种方法。 a) 电子邮件

b) 短信

您用于接收 10 美元电子礼品卡的电子邮件地址？

您用于接收 10 美元电子礼品卡的手机号码？

您是否有兴趣与访谈员详谈您最近的医护服务体验？会另外给一张 **25** 美元的电子礼品 卡，感谢您花 30 分钟参加保密访谈。

a) 是，我有兴趣

b) 否，我没有兴趣

为了帮您计划即将到来的访谈：

哪个电话号码最容易联系上您？

哪个电子邮件地址最容易联系上您？

在接下来的两周内，研究人员会在您方便时给您打电话。请选择适合您日程安排的所有 时间段。所列时间使用太平洋标准时间（加州时间）。

a) 平日（周一至周五）上午 11 点至下午 2 点

b) 平日（周一至周五）下午 3 点至下午 6 点

c) 周六上午 9 点至下午 1 点

d) 其他

如果选择了“其他”，一周中哪些日子和时间对您最方便？

感谢您有兴趣参加访谈。请从此日历中选择一个日期和时间安排您的访谈。访谈前会给 您发送确认信息。

结束语：

1）“由于您不同意参与，调查已结束。如果您仍想参与，请刷新浏览器重新开始调查。 此处包含研究信息表的链接。”

2) “感谢您完成调查并有兴趣参加访谈。您的反馈将有助于为像您这样的患者改善服 务。在未来几天内会与您确认您的确切访谈日期和时间。此处包含研究信息表的链 接。”

3) “感谢您完成调查。您的回复将有助于改善为像您这样的患者提供的服务。此处包含 研究信息表的链接。”

Dari

سروی مریض

در خانه به کدام لسان صحبت می‌کنید؟

عربی
ارمنی
برمه‌ای
کامبوجیایی (خمر مرکزی)
چینایی (کانتونی)
چینایی (ماندارین)
دری
فارسی
کریول هائیتی
هندی
همونگ
جاپانی
کارین
کوریایی
لائوسی
اورومو
پشتو
پرتغالی
پنجابی
روسی
سومالی
اسپانیایی
سواحیلی
تاگالوگ
تایلندی
تیگرینیا
اوکراینی
ویتنامی
غیره

1. تاریخ تولد شما:

2. آخرین تاریخ قرارملاقات شما در کلینیک:

3. آیا ارائه‌دهنده به گپ‌های شما به دقت گوش داد؟
   a. بلی، کاملا
   b. بلی، تاحدودی
   c. خیر

4. آیا ارائه‌دهنده به چیزهایی که می‌گفتیداحترام می‌گذاشت؟
   a. بلی، کاملا
   b. بلی، تاحدودی
   c. خیر

5. آیا ارائه‌دهنده شما را تشویق می‌کرد که سوال بپرسید؟
   a. بلی، کاملا

b. بلی، تاحدودی
c. خیر

6. آیا ارائه‌دهنده برای شما وقت کافی صرف کرد؟
a. بلی، کاملا
b. بلی، تاحدودی
c. خیر

7. آیا ترجمان به شما کمک کرد احساس خود را برای ارائه دهنده توضیح بدهید؟
a. بلی، کاملا
b. بلی، تاحدودی
c. خیر

8. آیا ترجمان به شما کمک کرد تا دستورات ارائه‌دهنده را متوجه شوید؟
a. بلی، کاملا
b. بلی، تاحدودی
c. خیر

9. آیا ترجمان با ادب و احترام با شما رفتار کرد؟
a. بلی، کاملا
b. بلی، تاحدودی
c. خیر

10. از بین عدد 0 الی 10، 0 یعنی بدترین ترجمان ممکن و 10 یعنی بهترین ترجمان ممکن، برای امتیاز دادن به این ترجمان از کدام عدد استفاده می‌کنید؟
a. 0 بدترین ترجمان ممکن
b. 1
c. 2
d. 3
e. 4
f. 5
g. 6
h. 7
i. 8
j. 9
k. 10 بهترین ترجمان ممکن

11. آیا ارائه‌دهنده از شما خواست برای یک قرار ملاقات تعقیبیه دوباره به او مراجعه نمایید؟
a. بلی
b. خیر

12. آیا برای قرار ملاقات تعقیبیهٔ شما حضور ترجمان پلان‌گذاری شد؟
a. بلی
b. خیر
c. نمی‌دانم

13. آیا ارائه‌دهنده به شما گفت برای دوا نسخه نوشته است؟
a. بلی
b. خیر

14. آیا ترجمان به شما کمک کرد که متوجه شوید چی‌قسم باید دوا را مصرف کنید؟

a. بلی، کاملا
b. بلی، تاحدودی
c. خیر

15. آیا شما می‌دانستید که یک ترجمان می‌تواند دستورهای مصرف دوای تجویزی را ترجمه کند؟
a. بلی
b. خیر

16. در مدت 12 ماه گذشته، آیا بدون حضور ترجمان کدام قرار ملاقاتی داشته‌اید؟
a. بلی
b. خیر

17. وقتی که ترجمان نداشتید، کِی برای شما ترجمه می‌کرد؟ تمام مواردی که صحیح هستند را نشانی کنید.
a. دوست
b. اعضای فامیل
c. کارمند کلینیک

18. قرار ملاقات آخرتان با حضور یک ترجمان چه تفاوتی با قرار ملاقات‌های قبلیتان بدون حضور ترجمان داشت؟
a. بهتر
b. تقریبا هیچ فرقی نداشت
c. بدتر

19. برای امتیاز دادن به آخرین تجربه خود در کلینیک از کدام عدد استفاده می‌کنید؟ از بین عدد 0 الی 10 استفاده کنید، 0 یعنی بدترین تجربه ممکن در کلینیک و 10 بهترین تجربه ممکن در کلینیک.
a. 0 بدترین تجربه ممکن
b. 1
c. 2
d. 3
e. 4
f. 5
g. 6
h. 7
i. 8
j. 9
k. 10 بهترین تجربه ممکن

20. جنسیت شما چیست؟
a. مرد
b. زن
c. غیره

21. توانایی شما در صحبت کردن به لسان انگلیسی چقدر است؟
a. بسیار خوب
b. خوب
c. خوب نیست
d. هیچ

22. به حفظ‌الصحه عمومی خود چند امتیاز می‌دهید؟
a. عالی
b. بسیار خوب

c. خوب
d. متوسط
e. ضعیف

به خاطر وقتی که برای اشتراک در این مطالعه صرف نمودید، از شما تشکر می‌کنیم. ما تا 48 ساعت آینده به ایمیل آدرس شما یا به تلیفون همراه شما بصورت پیام یک کارت هدیه الکترونیک 10 دالری ارسال خواهیم کرد. لطفا بهترین روش مورد نظر خود را انتخاب نمایید.

a) ایمیل
b) پیام

ایمیل آدرس شما برای ارسال کارت هدیه الکترونیک 10 دالری چیست؟

نمبر تلیفون همراه شما برای ارسال کارت هدیه الکترونیک 10 دالری چیست؟

آیا می‌خواهید برای یک مصاحبه کننده تجربه آخر خود را بیشتر توضیح دهید؟ ما برای اشتراک شما در یک مصاحبه محرمانه 30 دقیقه‌ای یک کارت هدیه 25 دالری دیگر خواهیم داد.

a) بلی، علاقه دارم
b) خیر، علاقه ندارم

برای کمک به پلان‌گذاری مصاحبهٔ پیش رو:

بهترین نمبر تلیفون برای تماس با شما چیست؟

بهترین ایمیل آدرس برای تماس با شما چیست؟

تا دو هفته آینده در زمانی که صحت تان مناسب باشد یک محقق با شما تماس خواهد گرفت. لطفا تمام فاصله‌های زمانی که مناسب پلان‌گذاری شما هستند را انتخاب نمایید. زمان‌ها به وقت استندرد اقیانوس آرام (به وقت کالیفرنیا) اعلان شدند.

a) روزهای هفته (دوشنبه الی جمعه) 11 قبل‌ازچاشت – 2 بعدازچاشت
b) روزهای هفته (دوشنبه الی جمعه) 3 بعدازچاشت – 6 بعدازچاشت
c) شنبه 9 قبل‌ازچاشت – 1 بعدازچاشت
d) غیره

اگر «دیگر» را انتخاب می‌کنید، کدام روزهای هفته و کدام اوقات مناسب پلان‌گذاری شما می‌باشند؟

از علاقه شما به اشتراک درین مصاحبه تشکر می‌کنیم. لطفا برای پلان‌گذاری مصاحبه خود از این تقویم یک تاریخ و زمان انتخاب نمایید. قبل از مصاحبه شما یک پیام تایید برایتان ارسال خواهد شد.

پایان‌ها:

1) «سروی خاتمه یافت؛ چون شما برای اشتراک نمودن رضایت ندادید. اگر تا فعلاً می‌خواهید اشتراک نمایید، لطفا بروزر خود را رفرش کنید تا سروی از اول شروع شود. یک لینک به ورق معلومات مطالعه در اینجا قرار داده شده است.»

2) « از اشتراک شما در این سروی و ازعلاقه شما برای اشتراک درین مصاحبه تشکر می‌کنیم. نظرات شما به بهتر شدن خدمات برای مریضانی مثل شما کمک خواهد کرد. تاریخ و زمان دقیق مصاحبه شما در روزهای آینده با شما تایید خواهند شد. یک لینک به ورق معلومات مطالعه در اینجا قرار داده شده است.»

3) « به خاطر پر نمودن سروی از شما تشکر می‌کنیم. جواب‌های شما به بهتر شدن خدمات برای مریضانی مثل شما کمک خواهند کرد. یک لینک به ورق معلومات مطالعه در اینجا قرار داده شده اس

Farsi

نظرسنجی بیمار

در خانه به چه زبانی صحبت می کنید؟

عربی
ارمنی
برمه ای
کامبوجی (خمر مرکزی)
چینی (کانتونی)
چینی (ماندرین)
دری
فارسی
کریول هائیتی
هندی
همونگ
ژاپنی
کارن
کره ای
لائوس
اورومو
پشتو
پرتغالی
پنجابی
روسی
سومالیایی
اسپانیایی
سواحیلی
تاگالوگ
تایلندی
تیگرینیا
اوکراینی
ویتنامی
سایر:

1. تاریخ تولد شما:

2. تاریخ آخرین قرار مالقات با کلینیک:

3. آیا ارائه دهنده با دقت به شما گوش داد؟
a. بله، قطعا

b. بله، تاحدودی
c. خیر

4. آیا ارائه دهنده به آنچه شما می گفتید احترام گذاشت؟
a. بله، قطعا
b. بله، تاحدودی
c. خیر

5. آیا ارائه دهنده شما را تشویق به پرسیدن سوال کرد؟
a. بله، قطعا
b. بله، تاحدودی
c. خیر

6. آیا ارائه دهنده زمان کافی را با شما صرف کرد؟
a. بله، قطعا
b. بله، تاحدودی
c. خیر

7. آیا مترجم به شما کمک کرد توضیح دهید که چه احساسی نسبت به ارائه دهنده داشتید؟
a. بله، قطعا
b. بله، تاحدودی
c. خیر

8. آیا مترجم به شما در درک دستورالعمل های ارائه دهنده کمک کرد؟
a. بله، قطعا
b. بله، تاحدودی
c. خیر

9. آیا مترجم با شما با ادب و احترام برخورد کرد؟
a. بله، قطعا
b. بله، تاحدودی
c. خیر

10. با استفاده از اعداد 0 تا 10، که در آن 0 بدترین مترجم ممکن و 10 بهترین مترجم ممکن است، از چه عددی برای امتیاز دادن به این مترجم استفاده می کنید؟
a. 0 بدترین مترجم ممکن
b. 1
c. 2
d. 3
e. 4
f. 5
g. 6
h. 7
i. 8
j. 9
k. 10 بهترین مترجم ممکن

11. آیا ارائه دهنده از شما خواسته است که برای قرار مالقات بعدی مراجعه کنید؟
a. بله
b. خیر

12. آیا مترجمی برای قرار مالقات بعدی شما در نظر گرفته شده بود؟
a. بله

b. خیر
c. اطالعی ندارم

13. آیا ارائه دهنده به شما گفته است که برای دارو نسخه را پر کنید؟
a. بله
b. خیر

14. آیا مترجم به شما در درک نحوه مصرف دارو کمک کرد؟
a. بله، قطعا
b. بله، تاحدودی
c. خیر

15. آیا می دانستید که یک مترجم می تواند دستورالعمل های مصرف داروی نسخه ای را ترجمه کند؟ a. بله
b. خیر

16. در طول 12 ماه گذشته، آیا قرار مالقات بدون مترجم داشتید؟
a. بله
b. خیر

17. وقتی مترجم نداشتید چه کسی برای ترجمه به شما کمک کرد؟ همه موارد مرتبط را تیک بزنید.
a. دوست
b. عضو خانواده
c. کارکنان کلینیک

18. قرار مالقات اخیر شما با مترجم در مقایسه با قرارهای قبلی بدون مترجم تان چگونه بود؟ a. بهتر
b. تقریبا یکسان
c. بدتر

19. از چه عددی برای امتیاز دادن به آخرین تجربه خود در کلینیک استفاده می کنید؟ از اعداد 0 تا 10 استفاده کنید، که در آن 0 بدترین تجربه کلینیکی ممکن و 10 بهترین تجربه کلینیکی ممکن است.
a. 0 بدترین تجربه ممکن
b. 1
c. 2
d. 3
e. 4
f. 5
g. 6
h. 7
i. 8
j. 9
k. 10 بهترین تجربه ممکن

20. جنسیت شما چیست؟
a. مذکر
b. مونث
c. سایر

21. میزان تسلط شما به انگلیسی چقدر است؟
a. خیلی خوب است
b. خوب است

c. خوب نیست
d. اصال خوب نیست

22. سالمت کلی خود را چگونه ارزیابی می کنید؟
a. عالی
b. خیلی خوب
c. خوب
d. معمولی
e. بد

از شما متشکریم که برای شرکت در این مطالعه وقت گذاشتید. ظرف 48 ساعت آینده یک کارت هدیه الکترونیکی 10 دالری را به آدرس ایمیل یا تلفن همراهتان از طریق پیامک برای شما ارسال خواهیم کرد. لطفاً مشخص کنید که کدام روش برای شما بهتر است.
a) ایمیل
b) پیامک

آدرس ایمیل شما برای ارسال کارت هدیه الکترونیکی 10 دالری چیست؟

شماره تلفن همراه شما برای ارسال کارت هدیه الکترونیکی 10 دالری چیست؟

آیا عالقه مند هستید که درباره تجربه اخیر مراقبت های بهداشتی خود با جزئیات بیشتری با یک مصاحبه کننده صحبت کنید؟ ما یک کارت هدیه الکترونیکی 25 دالری اضافی برای شرکت شما در یک مصاحبه 30 دقیقه ای محرمانه ارائه می دهیم.
a) بله، من عالقه مندم
b) خیر، عالقه ای ندارم

برای کمک به برنامه ریزی برای مصاحبه آینده:

بهترین شماره تلفن برای دسترسی به شما چیست؟

بهترین آدرس ایمیل برای دسترسی به شما چیست؟

یک محقق طی دو هفته آینده در مدت زمانی که برای تان مناسب است، با شما تماس خواهد گرفت. لطفاً تمام پنجره های زمانی را انتخاب کنید که با برنامه شما همخوانی دارد. زمان ها به زمان استاندارد اقیانوس آرام (به وقت کالیفرنیا) فهرست شده اند.
a) روزهای هفته (دوشنبه تا جمعه) 11 صبح تا 2 بعد از ظهر
b) روزهای هفته (دوشنبه تا جمعه) از ساعت 3 بعد از ظهر تا 6 بعد از ظهر
c) شنبه 9 صبح تا 1 بعد از ظهر
d) سایر

اگر «سایر» را انتخاب کردید، چه روزهایی از هفته و چه زمانهایی برای برنامه شما مناسبتر است؟

از عالقه شما به شرکت در مصاحبه متشکریم. تاریخ و ساعتی را از <u>این تقویم</u> برای برنامه ریزی مصاحبه خود لطفاً انتخاب کنید. قبل از مصاحبه یک پیام تایید برای شما ارسال خواهد شد.

بخش های پایانی:

1) " نظرسنجی به دلیل عدم رضایت شما برای شرکت به پایان رسیده است. اگر همچنان می خواهید شرکت کنید،

کنید. لینکی به برگه اطالعات مطالعه در (Refresh(مرورگر خود را برای راه اندازی مجدد نظرسنجی بازخوانی ًلطفا
".گنجانده شده است اینجا

از شما برای تکمیل نظرسنجی و عالقه تان به شرکت در مصاحبه متشک ریم. نظرات شما به بهبود خدمات برای 2(
بیمارانی مانند شما کمک می کند. تاریخ و زمان دقیق مصاحبه تان در روزهای آینده با شما تایید خواهد شد. لینکی به
".برگه اطالعات مطالعه در اینجا گنجانده شده است

از شما برای تکمیل نظرسنجی متشکریم. پاسخ های شما به بهبود خدمات برای بیمارانی مانند شما کمک می کند. 3(
لینکی به برگه اطالعات مطالعه در اینجا
".گنجانده شده است

Haitian Creole

Sondaj pou Pasyan

Ki **lang** ou pale lakay ou?

Arab
Amenyen
Biman
Kanbodyen (Kemè Santral)
Chinwa (Kantonè)
(Chinwa (Mandaren)
Dari
Fasi
Kreyòl Ayisyen
Hendi
Hmong
Japonè
Karen
Koreyen
Lawotyen
owomo
Pasto
Pòtigè
Panjabi
Ris
Somalyen
Panyòl
Swahili
Tagalòg
Tayi
Tigrinya
Ikrenyen
Vyetnamyen
Lòt lang

1. Dat nesans ou:

2. Dat dènye randevou ou nan klinik la:

3. Èske pwofesyonèl swen sante a **te koute ou avèk anpil atansyon**? a.
   Wi, kare-bare
   b. Wi, enpe
   c. Non

4. Èske pwofesyonèl swen sante a **te montre respè** pou sa ou te dwe di a?
   a. Wi, kare-bare
   b. Wi, enpe
   c. Non

5. Èske pwofesyonèl swen sante a **te ankouraje ou pou poze kesyon**? a.
   Wi, kare-bare
   b. Wi, enpe
   c. Non

6. Èske pwofesyonèl swen sante a **te pase ase tan** avèk ou?
   a. Wi, kare-bare
   b. Wi, enpe
   c. Non

7. Èske entèprèt la te **ede ou eksplike** pwofesyonèl swen sante a fason ou te santi ou?
   a. Wi, kare-bare
   b. Wi, enpe
   c. Non

8. Èske entèprèt la **te ede ou konprann enstwiksyon** pwofesyonèl swen sante a te ba ou yo?
   a. Wi, kare-bare
   b. Wi, enpe
   c. Non

9. Èske entèprèt la te trete ou avèk **koutwazi ak respè**?
   a. Wi, kare-bare
   b. Wi, enpe
   c. Non

10. Avèk nenpòt chif ant 0 ak 10 , kote 0 reprezante entèprèt ki te pi enkonpetan posib la epi 10 reprezante entèprèt ki te pi bon posib la, **ki chif ou ta itilize pou klase entèprèt sa a**?
    a. 0Entèprèt ki te pi enkonpetan posib la
    b. 1
    c. 2
    d. 3
    e. 4
    f. 5
    g. 6
    h. 7
    i. 8
    j. 9
    k. 10 Entèprèt ki te pi bon posib la
11. Èske pwofesyonèl swen sante a te mande ou pou retounen pou yon lòt randevou?
    a. Wi
    b. Non

12. Èske entèprèt la te pwograme ou pou lòt randevou ou?
    a. Wi
    b. Non
    c. Mwen pa konnen

13. Èske pwofesyonèl swen sante a te mande ou pou egzekite yon preskripsyon pou

medikaman?

a. Wi
b. Non

14. Èske entèprèt la te **ede ou konprann** fason pou pran medikaman an? a. Wi, kare-bare
b. Wi, enpe
c. Non

15. Èske ou te konnen yon entèprèt kapab tradui enstwiksyon yo pou ou pou pran medikaman sou preskripsyon an?
a. Wi
b. Non

16. Pandan dènye 12 mwa yo, èske ou te gen yon randevou **san prezans** yon entèprèt?
a. Wi
b. Non

17. Kimoun ki te ede entèprete pou ou lè ou pa t gen yon entèprèt? Fè yon ti kwa nan tout sa ki konsène ou.
a. Zanmi
b. Manm Fanmi
c. Anplwaye klinik la

18. Kijan dènye randevou ou avèk entèprèt te konpare randevou **pase** ou yo san prezans yon entèprèt?
a. Pi bon
b. Apeprè menm jan
c. Pi mal

19. Ki chif pou ta itilize pou **klase dènye eksperyans ou** nan klinik la? Itilize nenpòt chif ant 0 ak 10, kote 0 reprezante pi move eksperyans posib nan klinik la epi 10 reprezante pi bon eksperyans posib nan klinik la.
a. 0 Pi move eksperyans posib
b. 1
c. 2
d. 3
e. 4
f. 5
g. 6
h. 7
i. 8
j. 9
k. 10 Pi bon eksperyans posib

20. Ki sèks ou?
a. Gason
b. Fanm
c. Lòt

21. Kijan ou pale lang Anglè?

a. Trè byen
b. Byen
c. Pa byen
d. Pa ditou

22.Kijan ou ta klase sante jeneral ou?
a. Ekselan
b. Bon Anpil
c. Bon
d. Pasab
e. Pa Bon

Mèsi pou tan ou pran pou patisipe nan etid sa a. Nou pral; voye ba ou yon chèk-kado elektwonik nan **adrès imèl ou oswa nan telefòn selilè ou sou fòm tèks** nan pwochen 48 èdtan yo. Tanpri endike ki metòd k ap travay pi byen pou ou.
a) Imèl
b) tèks

Kisa adrès imèl ou ye pou nou voye chèk-kado elektwonik la pou ou? Kisa nimewo

telefòn selilè ou ye pou nou voye chèk-kado elektwonik la pou ou?

Èske ou enterese diskite sou dènye eksperyans swen sante ou avèk plis detay avèk yon moun ki alatèt entèvyou a? Nou pral ba ou yon lòt **chèk-kado elektwonik $25** pou patisipasyon ou nan yon entèvyou konfidansyèl k ap dire 30 minit.
a) Wi, mwen enterese
b) Non, mwen pa enterese

Pou ede planifye pou pwochen entèvyou ou:

Nan ki **pi bon nimewo telefòn** pou nou kontakte ou?

Nan ki **pi bon adrès imèl** pou nou kontakte ou?

Yon chèchè pral rele ou nan de semèn pandan yon lè k ap bon pou ou. Tanpri chwazi **tout entèval lè k ap travay** avèk orè ou. Lè yo endike nan lè Zòn Estanda Pasifik(lè California)
a) Jou lasemèn (lendi jiska vandredi) 11AM-2PM
b) Jou lasemèn (lendi jiska vandredi) 3PM-6PM
c) Samdi 9AM-1PM
d) Lòt lè

Si ou chwazi lòt lè, **ki jou ak lè nan semèn nan** ki ta travay pou orè ou?

Mèsi paske ou enterese patisipe nan yon entèvyou. Tanpri chwazi yon dat ak lè nan almannak sa a pou pwograme pou entèvyou ou. Nou pral voye yon mesaj konfimasyon ba ou anvan entèvyou a.

Fen:
1) "Sondaj la fini paske ou pa t bay konsantman ou pou patisipe ladan. Si ou toujou vle patisipe, tanpri re-aktyalize navigatè ou pou rekòmanse sondaj la. Gen yon lyen k ap

mennen ou nan fich enfòmasyon etid sa a **la a**."

2) "Mèsi paske ou patisipe nan sondaj la epi pou enterè ou nan patisipe nan yon entèvyou. Kòmantè ou bay yo pral ede nou fè sèvis yo vin pi bon pou pasyan yo tankou ou. Nou pral konfime dat ak lè egzat entèvyou a avèk ou nan jou k ap vini yo. Gen yon lyen k ap mennen ou nan fich enfòmasyon etid sa a **la a**."

3) "Mèsi pou patisipasyon ou nan sondaj la. Repons ou bay yo pral ede nou fè sèvis yo vin pi bon pou pasyan yo tankou ou. Gen yon lyen k ap mennen ou nan fich enfòmasyon etid sa a **la a**."

Hindi

मरीज़ सर्वेक्षण

आप घर पर कौन-सी **भाषा** बोलतेहैं?

अरबी
आर्मेननयाई
बर्मी
कम्बोनियाई (र्मध्य खर्मेर)
चीनी (कें टोनीस)
चीनी (मैंिाररन)
दरी
फारसी
हैनतयन निओल
नहिंदी
हर्मोिंग
जापानी
करेन
कोररयाई
लाओनियन
ओरोर्मो
पश्तो
पुततगाली
पिंजाबी
रूसी
सोर्माली
स्पैननि
स्वानहली
टैगालॉग
थाई
नटग्रीन्या
यूिे ननयन
नियतनार्मी

अन्य

1. आपके जन्म की तारीख:

2. आपकी सबसेहानलया क्लिननक अपॉइंटमेंट की तारीख:

3. क्या प्रदाता (provider) नेआपकी बात **सार्वधानीपूर्वक सुनी**? a. हााँ,

ननननत रूप से

b. हााँ, कु छ हद तक

c. नही िं

4. क्या प्रदाता नेआपकी बातोिंके प्रनत **सम्मान दर्ावया**?

a. हााँ, ननननत रूप से

b. हााँ, कु छ हद तक

c. नही िं

5. क्या प्रदाता नेआपको **प्रश्न पूछनेके लिए प्रोत्सालित लकया**?

a. हााँ, ननननत रूप से

b. हााँ, कु छ हद तक

c. नही िं

6. क्या प्रदाता नेआपके साथ **पयावप्त समय लिताया**?

a. हााँ, ननननत रूप से

b. हााँ, कु छ हद तक

c. नही िं

7. क्या दुभानिए नेप्रदाता को यह स्पष्ट करनेमें**आपकी सिायता की** नक आप कै सा महसूस कर रहेहैं?

a. हााँ, ननननत रूप से

b. हााँ, कु छ हद तक

c. नही िं

8. क्या दुभानिए नेआपको प्रदाता **के लनदेर् समझनेमेंसिायता** की?

a. हााँ, ननननत रूप से

b. हााँ, कु छ हद तक

c. नही िं

9. क्या दुभानिए नेआपके साथ **लर्ष्टाचारपूणवऔर सम्मानजनक** व्यिहार नकया? a.

हाँ, ननननित रूप से

b. हाँ, कु छ हद तक

c. नही िं

10.0 से10 तक नकसी भी सिंख्या का उपयोग करतेहुए, जहाँिं 0 का मतलब हैसबसेखराब सिंभि दुभानिया, और 10 का मतलब हैबेहतरीन सिंभि दुभानिया, **आप इस दुभालषए को दजावदेनेके लिए लकस संख्या का उपयोग करेंगे**?

a. 0 सबसेखराब सिंभि दुभानिया

b. 1

c. 2

d. 3

e. 4

f. 5

g. 6

h. 7

i. 8

j. 9

k. बेहतरीन सिंभि दुभानिया

11.क्या प्रदाता नेआपसेअनुिती (फॉलो अप) अपॉइिंटमेंट के नलए नफर सेआनेके नलए कहा? a.

हाँ

b. नही िं

12.क्या आपकी अनुिती अपॉइिंटमेंट के नलए नकसी दुभानिए को ननर्ातररत नकया गया था? a. हाँ

b. नही िं

c. मुझेनही िंपता

13.क्या प्रदाता नेआपको नलखी गई दिाइयाँप्राप्त करनेके नलए कहा?

a. हाँ

b. नही िं

14.क्या दुभानिए नेआपको यह **समझनेमेंसिायता** की नक आपको दिाइयाँकैसे लेनी हैं? a. हाँ, ननननित रूप से

b. हाँ, कु छ हद तक

c. नही ि ं

15.क्या आप जानतेथेनक दुभानिया पची मेंनलखी दिाइयााँलेनेके ननदेिोिं का अनुिाद कर सकता है?

a. हााँ

b. नही ि ं

16.नपछले12 र्माह के दौरान, क्या आपकी नकसी दुभानिए के **लिना** अपॉइंटर्मेंट थी? a. हााँ

b. नही ि ं

17.जब आपके पास कोई दुभानिया नही ि ंथा. तब बातचीत को सर्मझनेर्मेंनकसनेआपकी सहायता की? लागूहोनेिालेसभी पर सही का ननिान लगाएिं।

a. नर्मत्र

b. पररिार का सदस्य

c. क्लिननक स्टाफ

18.नकसी दुभानिए के नबना **पूर्वव**र्मेंआपकी अपॉइंटर्मेंट की तुलना र्मेंदुभानिए के साथ आपकी हाल ही की अपॉइंटर्मेंट कै सी थी?

a. बेहतर

b. लगभग सर्मान

c. बदतर

19.क्लिननक र्में**आपके सिसेिाि के अनुभर्व को दजावदेनेके लिए** आप नकस सिंख्या का उपयोग करेंगे? 0 से10 के बीच नकसी भी सिंख्या का उपयोग करें, जहााँ0 सबसेखराब सिंभि क्लिननक अनुभि हैऔर 10 सितश्रेष्ठ सिंभि क्लिननक अनुभि है।

a. 0 सबसेखराब सिंभि अनुभि

b. 1

c. 2

d. 3

e. 4

f. 5

g. 6

h. 7

i. 8

j. 9

k. 10 सितश्रेष्ठ सिंभि अनुभि

20.आप स्त्री हैंया पुरुि?

a. पुरुि
b. स्त्री
c. अन्य

21.आप नकतनी अच्छी अिंग्रेजी बोलतेहैं?

a. बहुत अच्छी
b. अच्छी
c. अच्छी नही िं
d. नबलकु ल नही िं

22.आप कु ल नर्मलाकर अपनेस्वास्थ्य को क्या दजातदेंगे?

a. उत्कृ ष्ट
b. बहुत अच्छा
c. अच्छा
d. ठीक-ठाक
e. खराब

इस अध्ययन र्मेंभाग लेनेके नलए सर्मय ननकालनेके नलए न्यिाद। हर्म अगले48 घिंटोिंके भीतर **आपके ईर्मेि पतेपर या आपके सेि फोन पर टेक्स्ट द्वारा** $10 का इलेक्ट्र ॉननक नगफ्ट काितभेजेंगे। कृ पया दिातएँनक कौन-सा तरीका आपके नलए सबसेअच्छा है।

a) ईर्मेल
b) टेक्स्ट

$10 का इलेक्ट्र ॉननक नगफ्ट काितभेजनेके नलए आपका ईर्मेल पता क्या है?

$10 का इलेक्ट्र ॉननक नगफ्ट काितभेजनेके नलए आपका सेल फोन निंबर क्या है?

क्या आप नकसी साक्षात्कारकतातके साथ अपनेहाल ही के स्वास्थ्य देखभाल अनुभि पर अनर्क निस्तार के साथ चचातकरनेके इच्छु क हैं? हर्म 30-नर्मनट के गोपनीय साक्षात्कार र्मेंआपकी भागीदारी के नलए **$25 का अलतररक्त इिक्ट्र ॉलनक लगफ्ट कार्व**प्रदान करेंगे।

a) हााँ, र्मैंइच्छु क हाँ
b) नही िं, र्मैंइच्छु क नही िंहाँ

आपके आगार्मी साक्षात्कार की योजना बनानेर्मेंसहायता के नलए:

आपसेसिंपकत करनेके नलए सबसेउनचत **फोन नंिर** कौन-सा है?

आपसेसिंपकत करनेके नलए सबसेउनचत **ईमेि पता** क्या है?

एक अनुसिंर्ानकतातआपको अगलेदो सप्ताह के भीतर आपके नलए सुनिर्ाजनक सर्मय पर आपको कॉल करेगा। कृ पया िे**सभी समय-अर्वलधयााँचुनेंजो आपकी समय-सारणी के अनुकू ि** होिं। सर्मय पैनसनफक स्टैंिितटाइर्म (कै नलफ़ोननतया टाइर्म) मेंनदया गया है।

a) कायतनदिस (सोर्मिार सेिुििार) सुबह 11 बजेसेदोपहर 2 बजेतक

b) कायतनदिस (सोर्मिार सेिुििार) दोपहर 3 बजेसेिार्म 6 बजेतक

c) िनिनिार सुबह 9 बजेसेदोपहर 1 बजेतक

d) अन्य

यनद आप “अन्य” का निकल्प चुनतेहैं, तो **सप्ताि के कौन सेलदन और समय** आपकी सर्मय-सारणी के अनुसार सबसेसही होिंगे?

साक्षात्कार मेंभाग होनेकी इच्छा दितनेके नलए न्यिाद। कृ पया अपनेसाक्षात्कार का सर्मय ननर्ातररत करनेके नलए **इस कै िेंर** सेकोई तारीख और सर्मय चुनें। आपके साक्षात्कार सेपहलेआपको पुनष्ट सिंदेि भेजा जाएगा।

सर्माक्लप्त:

1) “सिक्षण सर्माप्त हो गया हैक्योिंनक आपनेभाग लेनेकी सहर्मनत नही िंदी। यनद आप अभी भी भाग लेना चाहतेहैं, तो कृ पया सिक्षण को नफर सेिुरू करनेके नलए अपनेब्राउजर को ररफ्रे ि करें । अध्ययन जानकारी िीट का नलिंक **यिााँ**नदया गया है।"

2) “सिक्षण पूरा करनेऔर साक्षात्कार मेंभाग लेनेकी इच्छा दितनेके नलए न्यिाद। आपकी राय से आपके जैसेरोनगयोिंके नलए सेिाओंमेंसुर्ार करनेमेंसहायता नर्मलेगी। आपके साक्षात्कार की सही-सही तारीख और सर्मय की पुनष्ट आनेिालेनदनोिंमेंकी जाएगी। अध्ययन जानकारी िीट का नलिंक **यिााँ**नदया गया है।"

3) “सिक्षण पूरा करनेके नलए न्यिाद! आपके उत्तरोिंसेआपके जैसेरोनगयोिंके नलए सेिाओंमेंसुर्ार करनेमेंसहायता नर्मलेगी। अध्ययन जानकारी िीट का नलिंक **यिााँ**नदया गया है।"

Hmong

Qhov Kev Nug Neeg Mob

Yam **lus** koj siv hauv tsev yog lus dab tsi?

Lus Arabic
Lus Armenian
Neeg Phab Mab
Lus Qab Meem (Hais Nyob Ntu Nruab Nrab)
Lus Suav (Cantonese)
Lus Suav (Mandarin)
Lus Dari
Lus Farsi
Lus Haitian Creole
Lus Khej
Lus Hmoob
Lus Nyij Poom
Lus Karen
Lus Kaus Lim
Lus Nplog
Lus Oromo
Lus Pashto
Lus Portuguese
Lus Punjabi
Lus Lav Xia
Lus Somali
Lus Mev (Spanish)
Lus Swahili
Lus Fis Lis Peej
Lus Thaib
Lus Tigrinya
Lus Ukrainian
Lus Nyab Laj
Lwm Yam Lus

1. Koj hnub yug:

2. Hnub uas koj nyuam qhuav mus ntsib kws kho mob los sis mus kho mob tag los sai tshaj ntawm no:

3. Tus kws kho mob puas **ua tib zoo mloog** thaum koj hais lus?
   a. Yog, nws yeej ua tib zoo mloog
   b. Yog, nws yeej mloog me ntsis
   c. Tsis yog, nws yeej tsis mloog li
4. Tus kws kho mob puas **hwm** koj rau cov lus uas koj hais?
   a. Yog, nws yeej ua tib zoo mloog
   b. Yog, nws yeej mloog me ntsis
   c. Tsis yog, nws yeej tsis mloog li

5. Tus kws kho mob puas **hais kom koj nug** (yog koj tsis nkag siab ib yam dab tsi)?
   a. Yog, nws yeej hais kom nug
   b. Yog, nws yeej hais me ntsis
   c. Tsis yog, nws yeej tsis hais li

6. Tus kws kho mob puas **siv sij hawm ntev txaus** coj los nyob nrog koj tham? a. Yog, nws yeej siv sij hawm txaus
   b. Yog, nws yeej siv me ntsis
   c. Tsis yog, nws yeej tsis siv li

7. Tus kws txhais lus puas **pab koj hais** rau tus kws kho mob hais saib koj xav li cas?
   a. Yog, nws yeej pab hais
   b. Yog, nws yeej pab hais me ntsis
   c. Tsis yog, nws yeej tsis pab hais li

8. Tus kws txhais lus puas **pab qhia kom koj nkag siab cov lus qhia** uas koj tus kws kho mob hais kom koj ua?
   a. Yog, nws yeej pab qhia
   b. Yog, nws yeej pab qhia thiab
   c. Tsis yog, nws yeej tsis pab qhia li

9. Tus kws txhais lus puas **ua lub siab ntev thiab hwm koj**?
   a. Yog, nws yeej ua kawg
   b. Yog, nws yeej ua me ntsis
   c. Tsis yog, nws yeej tsis ua li

10. Siv ib tus lej ntawm 0 mus txog 10, uas 0 yog ib tus kws txhais lus tsis zoo kiag li thiab 10 yog ib tus kws txhais lus uas txhais tauzoo tshaj plaws li, **tus lej twg yog tus koj yuav muab coj los siv rau tus kws txhais lus ntawm no?** a. 0 Tus kws txhais lus tsis zoo kiag li
    b. 1
    c. 2
    d. 3
    e. 4
    f. 5
    g. 6
    h. 7
    i. 8
    j. 9
    k. 10 Tus kws txhais lus zoo tshaj plaws li

11. Tus kws kho mob puas teem dua ib lub sij hawm rau koj rov qab mus kuaj ntxiv? a. Yog, nws yeej hais kawg
    b. Tsis yog, nws yeej tsis hais li

12. Lawv puas teem kom muaj ib tug kws txhais lus tuaj pab txhais lus rau thaum lub sij hawm uas lawv teem kom koj rov qab mus ntsib tus kws kho mob? a. Yog, lawv yeej teem
    b. Tsis yog, lawv yeej tsis teem li
    c. Kuv tsis paub

13. Tus kws kho mob puas hais kom koj mus nqa ib yam tshuaj uas nws sau ntawv rau koj mus nqa coj los siv?
    a. Yog, nws yeej hais
    b. Tsis yog, nws yeej tsis hais li

14. Tus kws txhais lus puas **pab qhia kom koj nkag siab cov lus qhia** koj siv cov tshuaj?
    a. Yog, nws yeej pab qhia kawg
    b. Yog, nws yeej pab qhia me ntsis
    c. Tsis yog, nws yeej tsis pab qhia li

15. Koj puas paub hais tias koj yeej thov tau kom lawv nrhiav ib tug kws txhais lus los pab muab cov lus qhia koj siv tshuaj txhais rau koj?
    a. Kuv paub
    b. Kuv tsis paub

16. Li ntawm 12 lub hlis los rau tam sim no, koj puas tau mus ntsib kws kho mob ib zaug uas lawv **tsis muaj** ib tus kws txhais lus rau koj?
    a. Yog, kuv yeej tau mus
    b. Tsis yog, kuv yeej tsis tau mus li

17. Leej twg yog tus pab txhais lus rau koj thaum koj tsis muaj ib tus kws txhais lus? Khij tag nrho txhua qhov uas yog.
    a. Ib tus phooj ywg
    b. Ib tus neeg hauv tsev neeg
    c. Ib tus neeg ua hauj lwm ntawm lub chaw kho mob

18. Zaum koj nyuam qhuav mus ntsib kws kho mob tau los tsis ntev no uas muaj ib tus kws txhais lus muaj qhov txawv zoo li cas thaum muab piv rau cov sij hawm koj ntsib kws kho mob **yav tag los** uas tsis muaj ib tus kws txhais lus? a. Muaj qhov zoo dua
    b. Zoo ib yam xwb
    c. Muaj qhov phem dua

19. Tus lej twg yog tus koj yuav muab **siv rau zaum koj nyuam qhuav mus siv lub chaw kho mob tag los sai tshaj** no? Siv ib tus lej ntawm 0 mus txog 10, uas 0 yog ib zaug uas tsis zoo kiag li thiab 10 yog ib zaug uas zoo tshaj plaws li. a. 0 Ib zaug uas tsis zoo kiag li
    b. 1
    c. 2
    d. 3
    e. 4
    f. 5
    g. 6
    h. 7
    i. 8
    j. 9
    k. 10 Ib zaug uas zoo tshaj li

20. Koj yog poj niam los yog txiv neej (yog tub los yog ntxhais)?
    a. Txiv neej/tub
    b. Poj niam/ntxhais
    c. Lwm Yam

21. Koj hais tau lus Askiv zoo npaum li cas?
    a. Hais tau zoo heev
    b. Hais tau zoo
    c. Hais tsis tau zoo pes tsawg
    d. Hais tsis tau li

22. Koj xav hais tias koj txoj kev noj qab nyob zoo yog muaj zoo li cas? a. Zoo heev li
    b. Zoov heev
    c. Zoo
    d. Zoo txog ib nrab
    e. Tsis zoo

Ua koj tsaug rau lub sij hawm uas koj tau siv coj los koom qhov kev tshawb fawb ntawm no. Peb yuav muab ib daim npav khoom plig $10 xa tuaj rau **koj tus email los sis tuaj rau koj tus xov tooj ntawm tes uas yog muab xa ua ntaub ntawv** tsis pub dhau 48 teev tom ntej no. Thov qhia saib qhov twg yog qhov yuav zoo tshaj rau koj. a) Xa ua ntawv email
b) Xa ua ntawv hauv xov tooj

Tus email uas koj yuav kom peb xa daim npav khoom plig $10 mus rau yog tus twg?

Tus xov tooj ntawm tes uas koj yuav kom peb xa daim npav khoom plig $10 mus rau yog tus twg?
Koj puas kam nrog ib tus kws tshawb fawb sib tham ntau ntxiv hais txog zaum uas koj nyuam qhuav mus kho mob tag los tsis ntev no? Peb yuav muab ib **daim npav khoom plig $25** ntxiv rau koj yog koj mus koom ib qhov kev sib tham ntev li 30 feeb nrog ib tus kws tshawb fawb.
a) Yogkuv kam mus
b) kuv tsis kam mus

Los mus pab koj npaj rau koj qhov kev sib tham uas yuav muaj tom ntej no:

Tus xov tooj uas peb yuav hu tau rau koj yog tus twg?

Tus email uas peb yuav sau tau email tuaj rau koj yog tus twg?

Ib tus kws tshawb fawb yuav hu xov tooj tuaj rau koj nyob hauv ob lim piam tom ntej no rau thaum ib lub sij hawm uas koj yuav nrog nws tham tau. Thov xaiv **tag nrho cov sij hawm uas koj yuav khoom** los nrog tus kws tshawb fawb tham. Cov sij hawm yog muab teev rau Pacific Standard Time (sij hawm nyob hauv California).
a) Cov Hnub Qhib Hauj Lwm Hauv Lub Lim Piam (Hnub Monday mus txog Hnub Friday) 11AM-2PM
b) Cov Hnub Qhib Hauj Lwm Hauv Lub Lim Piam (Hnub Monday mus txog Hnub Friday) 3PM-6PM
c) Hnub Saturday 9AM-1PM
d) Lwm lub sij hawm

Yog koj xaiv qhov hais tias "lwm lub sij hawm", **cov hnub hauv lub lim piam thiab cov sij hawm twg** yuav yog cov zoo tshaj rau koj?

Ua tsaug rau qhov uas koj txaus siab yuav mus koom ib qhov kev sib tham. Thov xaiv ib

hnub thiab ib lub sij hawm nyob ntawm **daim ntawv teev cov hnub ntawm no** coj los teem koj qhov kev sib tham. Peb yuav xa ib tsab xov tuaj rau koj ua ntej koj qhov kev sib tham.

Cov kev xaus:

1) "Qhov kev nug xaus lawm vim koj tsis pom zoo mus teb. Yog koj tseem xav mus teb, thov muab koj qhov browser nias kom rov qab rub phab ntawv tawm los dua coj los pib qhov kev nug dua. Peb yeej muab ib kab ntawv txuas mus (link) rau phab ntawv teev cov lus qhia txog qhov kev tshawb fawb coj los rau **ntawm no**."

2) "Ua tsaug rau qhov uas koj xav mus teb qhov kev nug thiab qhov uas koj txaus siab xav mus koom ib qhov kev sib tham. Cov lus uas koj qhia rau peb yuav pab peb kho cov kev pab uas peb muaj rau cov neeg mob zoo li koj. Peb mam li qhia meej rau koj paub meej li ob peb hnub tom ntej no hais tias hnub twg thiab lub sij hawm twg yog hnub thiab yog lub sij hawm uas peb yuav muaj koj qhov kev sib tham. Peb yeej muab ib kab ntawv txuas mus (link) rau phab ntawv teev cov lus qhia txog qhov kev tshawb fawb coj los rau **ntawm no**."

3) "Ua tsaug rau qhov uas koj tau mus teb qhov kev nug tag lawm. Cov lus teb yuav pab peb kho cov kev pab uas peb muaj rau cov neeg mob zoo li koj. Peb yeej muab ib kab ntawv txuas mus (link) rau phab ntawv teev cov lus qhia txog qhov kev tshawb fawb coj los rau **ntawm no**."

Japanese

患者（かんじゃ）さまアンケート

あなたが自宅で話される言語はなんですか？

アラビア語

アルメニア語

ビルマ語

カンボジア語（中央クメール語）

中国語（広東語）

中国語（北京語）

ダリ語

ペルシャ語

ハイチ・クレオール語

ヒンディー語

ミャンマー語

日本語

カレン語

韓国語

ラオス語

オロモ語

パシュトー語

ポルトガル語

パンジャーブ語

ロシア語

ソマリ語

スペイン語

スワヒリ語

タガログ語

タイ語

ティグリニャ語

ウクライナ語

ベトナム語

その他

1. あなたの生年月日をご記入下さい。

2. 一番最近の診療日（しんりょうび）はいつでしたか。

3. あなたを診察（しんさつ）した医療従事者（いりょうじゅうじしゃ）(プロバイダー)はあなたの話を注意深く聞いてくれましたか？

    a. はい、確実に

    b. はい、ある程度

    c. いいえ

4. プロバイダーはあなたが言いたかったことを尊重（そんちょう）してくれましたか？

    a. はい、確実に

    b. はい、ある程度

    c. いいえ

5. プロバイダーはあなたに質問をするように勧めて（すすめて）くれましたか？

    a. はい、確実に

    b. はい、ある程度

    c. いいえ

6. プロバイダーはあなたと十分な時間を過ごしましたか？

    a. はい、確実に

b. はい、ある程度

c. いいえ

7. 通訳者（つうやくしゃ）はあなたの気持ちをプロバイダーに説明するのに役に 立ちましたか？

a. はい、確実に

b. はい、ある程度

c. いいえ

8. 通訳者（つうやくしゃ）はプロバイダーからの指示を理解するのに役に立ちま したか？

a. はい、確実に

b. はい、ある程度

c. いいえ

9. 通訳者（つうやくしゃ）は礼儀（れいぎ）正しく（ただしく）、敬意（けいい） をもってあなたに接しましたか？

a. はい、確実に

b. はい、ある程度

c. いいえ

10.0 から 10 までの数字で、0 が最悪の通訳者（つうやくしゃ）、10 が最良の通訳 者（つうやくしゃ）とすると、どのように評価しますか？

a. 0 最悪の通訳者

b. 1

c. 2

d. 3

e. 4

f. 5

g. 6

h. 7

i. 8

j. 9

k. 10 最良の通訳者

11.プロバイダーから、つぎの予約をするように言われましたか？ a.

はい

b. いいえ

12.つぎの予約の際に通訳者（つうやくしゃ）の参加も同時に予定しましたか？ a.

はい

b. いいえ

c. わかりません

13.プロバイダーは指定された薬を補充し、続けて服用するように指示しましたか? a.

はい

b. いいえ

14.通訳者（つうやくしゃ）は薬の服用方法を理解**するのに役に立ちましたか**？ a.

はい、確実に

b. はい、ある程度

c. いいえ

15.通訳者（つうやくしゃ）が薬を服用の仕方を翻訳（ほんやく）・ 通訳（つうやく） できることを知っていましたか？

a. はい

b. いいえ

16.過去 12 ヶ月間に通訳（つうやく）**なし**で予約に行きましたか? a.

はい

b. いいえ

17.専門（せんもん）の通訳者（つうやくしゃ）がいないときに、通訳（つうやく） を手伝ってくれたのはだれですか？あてはまるものをすべて選んでください。 a. 友達

b. 家族のメンバー

c. クリニックのスタッフ

18.直近の通訳（つうやく）付きの予約は、過去の通訳（つうやく）なしの予約と 比べてどうでしたか？

a. よかった

b. ほとんど変わらない

c. わるかった

19.あなたの直近**のクリニック（医**院・病院**）での経験**を 0 から 10 までの数字を 使って評価**するなら**、何点をつけますか？ 0 が最悪のクリニック経験とし、10 は最高のクリニック経験としてお答えください。

a. 0 最悪の経験

b. 1

c. 2

d. 3

e. 4

f. 5

g. 6

h. 7

i. 8

j. 9

k. 10 最高の経験

20.あなたの性別をお答えください?

a. 男性

b. 女性

c. その他

21.あなたは英語をどの程度話せますか？

a. とても上手に(ペラペラ)

b. 良い

c. あまりよくない

d. 全く話せない

22.全体的にみて、あなたの健康状態はどうですか?

a. 優れて（すぐれて）いる

b. かなり良い

c. 良い

d. そこそこ

e. 良くない

本アンケートにご協力いただき、ありがとうございました。48 時間以内に、10 ドル分 の電子ギフトカードを**電子メールアドレスまたは携帯電話（けいたいでんわ）にテキスト ト(ショート)メッセージ**にてお送りします。受け取る方法を選んでください。 a) 電子メール

b) テキスト(ショート)メッセージ

10 ドル分の電子ギフトカードを送信をするために、あなたの電子メールアドレスを教えてください。

10 ドルの電子ギフトカードを送るために、あなたの携帯番号を教えてください。

最近の医療経験（いりょうけいけん）について、担当者とより詳しい質問に回答するア ンケートに興味がありますか？30 分間の機密が守られるインタビューに参加いただけ ると、さらに **25 ドル**の電子ギフトカードを提供します。

a) はい、興味があります。

b) いいえ、興味ありません。

次のインタビューを受けるために、

一番連絡の取りやすい**電話番号**はなんですか？

一番連絡の取りやすい**メールアドレス**はなんですか？

2 週間以内に、ご都合のよい時間帯に担当者がお電話します。**ご都合の良い時間帯をすべ て**お選びください。時間帯は太平洋標準時（カリフォルニア時間）で表示されています。 a) 平日（月〜金）午前 11 時〜午後 2 時

b) 平日（月〜金）午後 3 時〜午後 6 時

c) 土曜日 午前 9 時〜午後 1 時

d) その他

「その他」を選択された場合、ご都合のよい曜日と時間帯をお聞かせください。

次のインタビューへの参加に興味をいただき、誠にありがとうございます。**このカレンダー**から日時を選んで、予約をお願いします。予約日の前に、確認（かくにん）メッ セージをお送り致します。

終了メッセージ:

1) 「参加に同意されなかったため、アンケートは終了しました。参加されたい場合は、 ブラウザを更新してアンケートを再開してください。研究情報シートへの**リンクは** ココです。」

2) 「アンケートにご協力いただきありがとうございました。また、インタビューにご 興味いただき、ありがとうございます。あなたのご意見は、患者（かんじゃ）さま へのサービス向上に役立たせていただきます。正式なインタビュー日時は、近日中 にご連絡いたします。 研究情報シートへの**リンクは**ココです。」

3) 「アンケートにご協力いただき、ありがとうございました。あなたの回答は、患者（かんじゃ）さまへのサービス向上に役立ちます。研究情報シートへの**リンクは** ココです。」

Karen

ပှၤဆါလံာ်တၢ်ကွၢ်ထံဆိမိၢ်

လၥ်နဟံဉ္ၥၢ်နသုမနၥ**ကျိၥ**လဲၥ။

ဗမဘောငခ အၤရၤဘုးကျိၥ
ဗမာနညငညေ အရမ့နံၤယၤကျိၥ
ကမာန္ဂျန ပယီၤကျိၥ
ဃဓာဘာသိငညေ (ဃနညအမဇ္၂ၥနမ) ခဘီဒ်ယၤကျိၥ ဗ(ခမဲရ)
ဃ့ငညန္ဂျန (ဃညေအသညန္ဂျန) တရှးကျိၥဗ (ခဲးထန်း)
ဃ့ငညန္ဂျန (ညေိမေညေ) တရှးကျိၥဗ(မဲတရှး)
မေင ဒါရံၥကျိၥ
မျေင ဖါဆံးကျိၥ
ငေအငညေ ဃမနသုန ဟ့ဂ့ဲခရံအိၥကျိၥ
ညိင ဟ့ၥဒ်ၥကျိၥ
ၥသညါ မီကျိၥ
စေညေန္ဂျန က္ခးပၥကျိၥ
မေနည ကညီကျိၥ
သမနညေခိၥရံၥယါကျိၥ
သေအငညေ လ့အိဂုၤကျိၥ
ၥမသၥသ အိၥရိမိၥကျိၥ
ရျအသ ဖစထိၥ
သမအကါကန္ဂျန ဖိၥတကံၢကျိၥ
ကညဖြဘင ဖက္ခါဘံး
ၤကျျငညေ ရဋ္ဌဂ့ါကျိၥ
သၥရူင စမီလံာ်ယၤကျိၥ
စညေဌျ စပဲနံး
တဇ္ငူင စ္ဂၤဟံးလံာ်
ဤါဒါရှသါ ထါကလီ
ဤါငေ ယိၤကျိၥ
ဤါငါမညေပဓ တကရှနယၤကျိၥ
မငညေငညေ ယူၤခရှကျိၥ
ဋ္ဌငနအညဓာန္ဂျန ဘံးယဲနၥကျိၥ
အဂုၤအဂၤ

၁ဤါဗဗဗနနံၤအိၥဖျဲၥမုၢနံၤါ

၂ဤါဗဗဗမုၢနံၤမုၢသီလၥ်နတၢထံၥလိၥတယံဒံးဘၥါ

၃ဤါဗဗဗမုၢနကသံၥသရၥဒိကနၢန**ၤလီၤတံၢလီၤဆဲး**စ့ၤဒါ။
၁ဤါဗဗဗမု္ၥမုၢခဲလၥ်ၥ
၂ဤါဗဗဗမု္ၥတနီၤ
၃ဤါဗဗဗတမုၢဘၥ

၄ဤါဗဗဗမုၢကသံသရၥပၥဖျါအ**တၢဟ့ကဲ**တၢလၥ်နတဲၤသ့ၥတဖၥဒါ။
ဗဗဗဗဗဗဗၥ၁ဤါဗဗဗမု္ၥ မုၢခဲလၥ်ၥ
ဗဗဗဗဗဗဗ ၂ဤါဗဗဗမု္ၥတနီၤ
ဗဗဗဗဗဗဗၥ၃ဤါဗဗဗတမုၢဘၥ

၅၍ဗဗဗမ့ၢကသံသရၣ**ဟ့ၣဂံၢဟ့ၣဘါနၤလဌနကသံကွၢ**ဒါ။
ဗဗဗဗဗဗဗဗဗ၁၍ဗဗဗမ့ၫ မ့ၢခဲလဌၢ
ဗဗဗဗဗဗဗဗဗ၂၍ဗဗဗမ့ၫဒိကနၣ
ဗဗဗဗဗဗဗဗဗ၃၍ဗဗဗတမ့ၢဘၣ

၆၍ဗဗဗမ့ၢကသံသရၣ**ဟ့ၣတၢဆဌကတိၢဒိးနၤလဌပှဲၤ**ဒါ။
ဗဗဗဗဗဗဗဗဗ၁၍ဗဗဗမ့ၫ မ့ၢခဲလဌၢ
ဗဗဗဗဗဗဗဗဗ၂၍ဗဗဗမ့ၫ တနီၤ
ဗဗဗဗဗဗဗဗဗ၃၍ဗဗဗတမ့ၢဘၣ

၇၍ဗဗဗမ့ၢပှၤကျိၥထံတၢ**မၤစဌၤနၤလဌကတဲပှၢ်**ကသံသရၣတၢလဌနတူၢဘၣတဖၣလဒါ။
ဗဗဗဗဗဗဗဗဗ၁၍ဗဗဗမ့ၫ မ့ၢခဲလဌၢ
ဗဗဗဗဗဗဗဗဗ၂၍ဗဗဗမ့ၫတနီၤ
ဗဗဗဗဗဗဗဗဗ၃၍ဗဗဗတမ့ၢဘၣ

၈၍ဗဗဗမ့ၢပှၤကျိၥထံတၢ**မၤစဌၤနၤဒိးဂဲ့ပှၢ်နၤတၢလဌကသံၣသရၣတဲဝဲ**ဒါ။
ဗဗဗဗဗဗဗဗဗ၁၍ဗဗဗမ့ၫ မ့ၢခဲလဌၢ
ဗဗဗဗဗဗဗဗဗ၂၍ဗဗဗမ့ၫ တနီၤ
ဗဗဗဗဗဗဗဗဗ၃၍ဗဗဗတမ့ၢဘၣ

၉၍ဗဗဗမ့ၢပှၤကျိၥထံတၢအိၣဒိးတၢ**သကဲးပဝဲဂ့ၤဒိးတၢဟကဲ**လဌနဖိခိၣဒါ။
ဗဗဗဗဗဗဗဗဗ၁၍ဗဗဗမ့ၫ မ့ၢခဲလဌၢ
ဗဗဗဗဗဗဗဗဗ၂၍ဗဗဗမ့ၫ တနီၤ
ဗဗဗဗဗဗဗဗဗ၃၍ဗဗဗတမ့ၢဘၣ

၁၀၍ဗဗဗသ့ဘၣနီၢဂံၢ(၀)တုၤနီၢဂံၢ(၁၀)ယနီၢဂံၢ(၀)မ့ၢအတဂ့ၤနီတစဲးဘၣဒိးနီၢ(၁၀)မ့ၢအဂ့ၤကတဌၢ**မ့ၢတကဟ့ၣလံၥလဌတၢကျိၥထံ
တၢအံၤနီၢဂံၢမ
နၥလဲၣ**။
ဗဗဗဗဗဗဗဗဗဗဗဗဗဗဗ(က)ဗ၀ ဗတဂ့ၤနီတဆဲးဘၣ
ဗဗဗဗဗဗဗဗဗဗဗဗဗဗဗ(ခ)ဗဗ၁
ဗဗဗဗဗဗဗဗဗဗဗဗဗဗဗ(ဂ)ဗဗ၂
ဗဗဗဗဗဗဗဗဗဗဗဗဗဗဗ(ဃ)ဗ၃
ဗဗဗဗဗဗဗဗဗဗဗဗဗဗဗ(င)ဗဗ၄
ဗဗဗဗဗဗဗဗဗဗဗဗဗဗဗ(စ)ဗဗ၅
(ဆ)ဗ၆
(ၦ)ဗဗ၇
(ည)ဗ၈
(တ)ဗ၉
(ထ)ဗ၁၀ ဗအဂ့ၤကတဌၢ

၁၁၍ဗဗဗမ့ၢကသံၣသရၣမဌနဟဲကဒါက့ၤထံၣလိၥသး ဒိးအီၤလဌသိဒ်ဒါ။
၁၍ဗမ့ၢ
၂၍ဗတမ့ၢ

၁၂၍ဗဗဗမ့ၢပှၤကိးဆဲးကျိၥပှၤကျိၥထံတၢလဌကဟ့ၣလဌနတၢဟဲထံၣလိၥသးကဒါက့ၤဒိးကသံၣသရၣဒါ။
ဗဗဗဗဗဗဗဗဗ၁၍ဗဗဗမ့ၫ မ့ၢခဲလဌၢ
ဗဗဗဗဗဗဗဗဗ၂၍ဗဗဗမ့ၫ တနီၤ
ဗဗဗဗဗဗဗဗဗ၃၍ဗဗဗတမ့ၢဘၣ

၁၃၍မ့ၢနကသံၣသရၣမၣနမၤပှဲၤနကသံၣကသီဒါ။
ဗဗဗဗဗဗဗဗဗ၁၍ဗမ့ၢ
ဗဗဗဗဗဗဗဗဗ ၂၍ဗတမ့
၁၄၍မ့ၢပှၤကျိၢထံတၢ**မၤစၢၤနၤလၢနၤ**ကဘၣအိကသံၣဒ်လဲၣ ဒ်လဲၣဒါ။
ဗဗဗဗဗဗဗဗဗ၁၍ဗဗဗမ့ၢ မ့ၢခဲလၢာ
ဗဗဗဗဗဗဗဗဗ ၂၍ဗဗဗမ့ၢ တနီၤ
ဗဗဗဗဗဗဗဗဗ၃၍ဗဗဗတမ့ၢဘၣ

၁၅၍ဗဗဗမ့ၢနသ့ၣညါစ့ၢဒါလၢပှၤကျိၢထံတၢတဖၣကျိၢထံသံၣကသီလၢနကဘၣအိအိၤဒ်လဲၣ်ၣ။
ဗဗဗဗဗဗဗဗဗ၁၍ဗမ့ၢ
ဗဗဗဗဗဗဗဗဗ ၂၍ဗတမ့ၢ

၁၆၍ဗဗဗပှၤကွံာ(၁၂)လါတီၢပူၤ်မ့ၢနတၢထံၣလိာ်သးလၢပှၤကျိၢထံတၢ**တအိၣ**ဘၣအိၣစ့ၢကီးဒါ။
ဗဗဗဗဗဗဗဗဗ၁၍ဗမ့ၢ
ဗဗဗဗဗဗဗဗဗ ၂၍ဗတမ့

၁၇၍ဗဗဗမတၤဂၤကျိၢထွံၢနၤတၢဖဲပှၤကျိၢထံတၢတအိၣအခါလဲၣ။ ဃုထၢတၢလၢဘၣထွဲတဖၣခဲလၢာ
ဗဗဗ၁၍ဗဗသကိး
ဗဗဗ၂၍ဗဗဟံၣဖိဃီဖိ
ဗဗဗ၃၍ဗဗတၢဆါဟံၣပှၤမၤနၢဖိ

၁၈၍ဗဗဗနတၢထံၣလိာ်သးလၢအဟဃၤဒီးပှၤကျိၢထံတၢဒီးနတၢထံၣလိာ်သးလၢအ**ပှၤကွံာ**လၢတဟဃၤဒီးပှၤကျိၢထံတ့ၢနမ့ၢထိၣသတြိ
ၤမၤသး ဒ်လဲၣ။
ဗဗဗ၁၍ဗဂ့ၤထိၣ
ဗဗဗ၂၍ဗဒ်လီၢလီၢ
ဗဗဗ၃၍ဗနးထိၣ

၁၉၍ဗဗဗနကဟ့ၣနီၢဂံၢထဲလဲၣဘၣဃး**နတၢဟဲလၢတၢဆါဟံၣ**တဘျီအံၤ။ သ့ဘၣနီၢဂံၢ (၀) တုၤနီၢဂံၢ(၁၀)ဃ
နီၢဂံၢ(၀)မ့ၢအတဂ့ၤနီတဆံးဘၣလၢနတၢဟဲတဘျီမံၤဒီးနီၢ(၁၀)မ့ၢအဂ့ၤကတၢၢလၢနတၢဟဲတဘျီအံၤၣ
ဗဗဗဗဗဗဗဗဗဗဗဗဗဗ(က)ဗ၀ ဗတၢ့ဘၣတမ့ာနီတဆံးဘၣ
ဗဗဗဗဗဗဗဗဗဗဗဗဗဗ(ခ)ဗဗ၁
ဗဗဗဗဗဗဗဗဗဗဗဗဗဗ(ဂ)ဗဗ၂
ဗဗဗဗဗဗဗဗဗဗဗဗဗဗ(ဃ)ဗ၃
ဗဗဗဗဗဗဗဗဗဗဗဗဗဗ(င)ဗဗ၄
ဗဗဗဗဗဗဗဗဗဗဗဗဗဗ(စ)ဗဗ၅
ဗဗဗဗဗဗဗဗဗဗဗဗဗဗ(ဆ)ဗ၆
ဗဗဗဗဗဗဗဗဗဗဗဗဗဗ(ၡ)ဗဗ၇
ဗဗဗဗဗဗဗဗဗဗဗဗဗဗ(ည)ဗ၈
ဗဗဗဗဗဗဗဗဗဗဗဗဗဗ(တ)ဗ၉
ဗဗဗဗဗဗဗဗဗဗဗဗဗဗ(ထ)ဗ၁၀ ဗတၢ့ဘၣလၢအဂ့ၤကတၢၢ

၂၀၍ဗဗဗနမ့ၢမနုၣလဲၣ။
၁၍ဗပှိာမှာ
၂၍ဗပှိာခွါ
၃၍ဗအဂ့အရၤ

၂၁၍ဗဗဗနတဲကိၤလၤဝါကျိၢဘၣစံးအါလဲၣ။
၁၍ဗဘၣဂ့ၤဂံၤ
၂၍ဗဘၣ
၃၍ဗတဘၣခဲလၢာ

၄၍ဗတဘၣနီတစံး

၂၂၍ဗဗဗနတၢအိၣ်ဆူၣ်အိၣ်ချ့အပတီၢ်အိၣ်ထဲလဲၣ်.
၁၍ဂ့ၤလီၤတံၢ်ခဲလၥ်
၂၍ဂ့ၤဖးဒိၣ်
၃၍ဂ့ၤ
၄၍ဖဲအကြၢၤ
၅၍တဂ့ၤ

တၢဘျုးလၢနဟ့ၣ်တၢဆၢကတီၢ်ဒီးမၤပှဲၤလံာ်တၢကွၢ်ထံဆိကမိၣ်အၢ်လီၤ၍ပကဆှၢနၤကံးခး(၁၀)ဒီလၢ**လၢနအံမ့ပှၤမ့တမ့ၢ်လၢနလီၤတဲစိပှၤခီဖျိ**
(ထံးမဲးစုး)ၢလီၤဖဲဗ(၄၈)နၢရံၢတီၢ်ပှၤလီၤ၍ဗဝံသးစူၤဃၢထၢဘၣ်ကျဲလၢအဂ့ၤလၢနဂီၢ်တက့ၢ်၍
၁၍ဗအံမ့
၂၍ဗထံးမဲးစုး

နအံမ့အဲးတြဲးလၢတၢကဆှၢ ကံးခး(၁၀)ဒီလၢအဂီၢ်မ့ၢ်မနၣ်လဲၣ်.

နလီတစိနီၢ်ဂံၢ လၢတၢကဆှၢ ကံးခး(၁၀)ဒီလၢအဂီၢ်မ့ၢ်မနၣ်လဲၣ်.

မ့ၢ်နအဲၣ်ဒီးသးအိၣ်တဲအါထီၣ်နဘၣ်ဃးနတၢအိၣ်ဆူၣ်အိၣ်ချ့ဒီးတၢမၤစၢၤနၤလၢကျဲမနၤလဲၣ်လၢနီၢ်ကစၢ်ဒၣ်ဝဲဓါ.
ပကဟ့ၣ်အါထီၣ်**(၂၅) ဒီလၢကံးခး**လၢပှၤလၢအနလီၤဝဲတၢတဲသကိးတၢ(အ့းထၢဘၣ်)အမံးနံး(၃၀)ၢလီၤ၍
၁၍ဗမ့ၢ်ယသးအိၣ်
၂၍ဗယသးတအိၣ်ဘၣ်

လၢတၢကကတီၤသးလၢတၢထံၣ်လိာ်သးဆူညါအဂီၢ်.

လီတစိနီၢ်ဂံၢ လၢတၢဆဲးကျိာ်နၤကွၢ်အဂ့ၤကတၢ်မ့ၢ်မနၣ်လဲၣ်.

အံမ့အဲးတြဲးလၢတၢကဆဲးကျိာ်နၤကွၢ်အဂ့ၤကတၢ်မ့ၢ်မနၣ်လဲၣ်.

ပှၤဃုသ့ၣ်ညါမၤလိအါထီၣ်တဖၣ်ကကိးနၤဖဲ(၂)နွံအတီၢ်ပှၤဖဲတၢဆၢကတီၢ်လၢအဂံၤကတၢ်လၢနဂီၢ်၍ဝံသးစူၤဃုထၢဘၣ်**တၢဆၢကတီၢ်လၢဂ့ၤကတၢ်**လၢန
ဂီၢ်တက့ၢ်၍တၢဆၢကတီၢ်တဖၣ်ဘၣ်တၢရဲၣ်လီၤအီၤ ဃနူၣ်သမညၤ‌ေ တၢဆၢကတီၢ်ၢလီၤ၍
၁၍ဗနွံတီၢ်ပှၤဗ(မ့ၢ်တနံၤတ့ၤမ့ၢ်ဃဲၢ်နံၤ)ဗဂီၤဗ၁၁ ဗ–ဗဟါဗ၂ဗနၢရံၣ်
၂၍ဗနွံတီၢ်ပှၤဗ(မ့ၢ်တနံၤတ့ၤမ့ၢ်ဃဲၢ်နံၤ)ဗဟါဗ၃ ဗ–ဗဟါဗ၆ဗနၢရံၣ်
၃၍ဗမ့ၢ်ဃုနံၤဗဂီၤဗ၉ ဗ–ဗဟါဗ၁ဗနၢရံၣ်
၄၍ဗအဂ့ၤအဂၤ

နမ့ၢ်ဃုထၢ ဓအဂ့ၤအဂၤဓမ့ၢ်နံၤတၢဆၢကတီၢ်လၢနတီၢ်အစ့ၤမ့ၢ်ဖဲလဲၣ်.

စံးဘျုးနၤလၢနသးအိၣ်မၤသကိးတၢဒီးဟ့ၣ်လၢတၢသံကွၢ်စံးဆၢတၢထံၣ်လိာ်သးၢလီၤ၍ဗဝံသးစူၤဃုထၢဘၣ်မ့ၢ်နံၤမ့ၢ်သ္ဓိတၢဆၢကတီၢ်
<u>တၢမၤနီၢ်</u>လၢတၢကမၤလီၤတံၢ်မ့ၢ်နံၤတၢထံၣ်လိာ်သးၢတက့ၢ်၍ တၢမၤဂၢ်မၤကျၢၤအမံးစုးကဘၣ်တၢဆှၢနၤတချုးတၢထံၣ်လိာ်သးၢလီၤ

အကတၢၢ်
၁)
ဓလံာ်တၢကွၢ်ထံဆိမိၢ်အံၤကတၢ်ခီဖျိနတအၥ်ၣ်လီၤလၢနကဟဃုာ်အဃိဘၣ်လီၤ၍နမ့ၢ်သးအိၣ်ဟဃုာ်ဝံးသးစူၤမၤသီထီၣ်က့ၤကဘျံးပာအံၤ
ဒ်သိးနကစးထီၣ်ကဒါက့ၤ လံာ်တၢကွၢ်ထံဆိမိၢ်အံၤတက့ၢ်၍ဗတၢမၤလိအစ့ၢ်အကျိၤတဖၣ်ဘၣ်တၢထၢနစ့ၢ်ကီးအီၤ**<u>ဖဲအံၤဓ</u>**

၂)
တၢ်ဘျုးလဌနမၤပှဲၤလံာ်တၢ်ကွၢ်ထံဆိမိၣ်အံၤဒီးလဌနသးအိၣ်ထံၣ်လိာ်သးလဌတၢ်သံကွၢ်စံးဆဌ့ၢလီၤ၍တၢ်လဌနဟ့ၣ်ထီၣ်တဖၣ်ကမ့ၢ်တၢ်မၤ
စဌၤလဌပှၤ
ဆါ ဒ်သိးနၤတဖၣ်လီၤ၍နတၢ်ထံၣ်
လိာ်သးလဌတၢ်သံကွၢ်စံးဆဌတၢ်ဆဌကတီ႗မ့ၢ်နံၤမ့ၢ်သီကဘၣ်တၢ်မၤဂဌၢ်ကျဌၤအီၤဒီးနၤနံၤလဌကဟ့ဌၢ်လီၤ၍
၍ဗတၢ်မၤလိအစ့ၢ်အကျိၤတဖၣ်ဘၣ်တၢ်ထဌနစ့ၢ်ကီးအီၤ**<u>ဖဲအံၤ</u>**

၃)တၢ်ဘျုးလဌနမၤပှဲၤလံာ်တၢ်ကွၢ်ထံဆိမိၣ်အံၤ၍တၢ်လဌနဟ့ၣ်ထီၣ်တဖၣ်ကမ့ၢ်တၢ်မၤစဌၤလဌပှၤဆါဒ်သိးနၤတဖၣ်လီၤ၍ဗတၢ်မၤလိအစ့ၢ်
အကျိၤတဖၣ်ဘၣ်
တၢ်ထဌနစ့ၢ်ကီးအီၤ**<u>ဖဲအံၤ</u>**

Khmer (Cambodian)

ការស្ទ ង់មតិអ្នកជមងឺ

ត ើអ្នកនិយាយ**ភាសា**ណាតៅកន ុងរបស់អ្នក ?

អារ៉ា ប់
អាម ិនតនៀន
ភូមា
ខ្មែរ (ខ្មែរកណា ា ល)
ចិន (កាតាំង)
ចិន (ក កងឺ)
ដារ ី ី
ហ្វា ស្ស ី ី
តេជីត្រីយ ូ ល
េ ិនឌី
េម ុង
ជប ន
ការ ិន
កូតរ៉ា
លាវ
អ្ ូរ៉ា ូម ូ
ផាសត ូ
ព័រទ យហ្គា ល់
ប នចាប ី ី
រ ស្ស ី ី
សូ មា លី
តអ្សា ញ
ស្វា េ ី ីលី
តកាឡស ក
ថៃ
ទីគ្រីញ
អ្ ិយតគ្រន
តវៀ ណាម
ភាសាតស្វៗ

1. កាលបរ ិតចេទ ឬ ថៃេ ខ្ ឆ្ន ា ំ កាំត ើ របស់អ្នក
2. កាលបរ ិតចេទ ឬ ថៃេ ខ្ ឆ្ន ា ំ ចនការណា ់ជួបតៅរលើនិកែេ ី ីតមែ ងបាំស របស់អ្នក ៖

3. ត ើអ្នកសាល់តសវា**សា ា ប់**អ្នក**ដោយយកចិតាទុកោក់**ឬតទ ?
   a. បាទ/ចាស់ ពិ ត្បាកដណាស់
   b. បាទ/ចាស់ មលះៗខ្លរ
   c. តទ

4. តើអ្នកសាល់គសវា**បង្ហាញការគោរព**ចំពោះអ្នកទាំងអ្វីខ្ពលអ្នក
បាននិយាយតរៀបរប់បឬតទ ? a. បាទ/ចាស់ ពិ ត្បាកដណាស់
b. បាទ/ចាស់ មលះៗខ្ពរ
c. តទ

5. តើអ្នកសាល់គសវា**ណែនាំប្រាប់អ្នកឲ្យស្រួ រស្រាំ ស្រូ វ**ឬតទ ?
a. បាទ/ចាស់ ពិ ត្បាកដណាស់
b. បាទ/ចាស់ មលះៗខ្ពរ
c. តទ

6. តើអ្នកសាល់គសវា**ចាំណាយដពលប្រប់ប្រោន់**ជាមួយអ្នកឬតទ ?
a. បាទ/ចាស់ ពិ ត្បាកដណាស់
b. បាទ/ចាស់ មលះៗខ្ពរ
c. តទ

7. តើអ្នកបកខ្សួបាន**ជួយអ្នកឲ្យពានយល់**អុំពីអារម្មណ៍របស់អ្នក
តៅដល់អ្នកសាល់គសវាឬតទ ? a. បាទ/ចាស់ ពិ ត្បាកដណាស់
b. បាទ/ចាស់ មលះៗខ្ពរ
c. តទ

8. តើអ្នកបកខ្សួបាន**ជួយអ្នកឲ្យយល់ពីការណែនាំ**ពីអ្នកសាល់គសវាខ្ពរឬតទ ?
a. បាទ/ចាស់ ពិ ត្បាកដណាស់
b. បាទ/ចាស់ មលះៗខ្ពរ
c. តទ

9. តើអ្នកបកខ្សួត្បក្ដីទាំងចាំតពោះអ្នក **ប្រប្រកបដោយភាពរួរស្ម និងការគោរព**ឬតទ?
a. បាទ/ចាស់ ពិ ត្បាកដណាស់
b. បាទ/ចាស់ មលះៗខ្ពរ
c. តទ

10. តដាយតគ្បីតលមណាមួយពី 0 ដល់ 10 ខ្ពល 0 រឺជាអ្នកបកខ្សួមិនលអឬអាត្កក់បាំស
ខ្ពលអ្នកបានជួប តហើយ 10 រឺជាអ្នកបកខ្សួលអបាំស ខ្ពលអាចដាក់តៅបាន **ដតើអ្នកនឹង
ដប្រើដលខអ្វីដើមបីវាយតម្លៃអ្នកបកណប្របដនេះ** ?
a. 0 អ្នកបកខ្សួអាត្កក់បាំស ឬមិនលអ ខ្ពលអ្នកបានជួប
b. 1
c. 2
d. 3
e. 4
f. 5
g. 6
h. 7
i. 8
j. 9
k. 10 អ្នកបកខ្សួលអបាំស ខ្ពលអ្នកបានជួប

11. ត ើើអ្នកសាល់តសវាអ្ន ើើញអ្នកឲ្យត្ ឡប់តៅវញិ សគ្នាប់ការណា ់ជួបតមដានបនាតទៀ ឬតទ ?
   a. បាទ/ចាស់
   b. តទ

12. ត ើើអ្នកបកខ្ល បានណា ់តពលទ កសគ្នាប់ការណា ់ជួបតមដានតអាយអ្នកឬតទ ? a. បាទ/ចាស់
   b. តទ
   c. មុ ុ៣ំមិនដឹងតទ

13. ត ើើអ្នកសាល់តសវាគ្សាប់អ្នកឲ្យបាំតពញ តវជជបញ្ជជសគ្នាប់មន ៣ំរបស់អ្នកឬតទ ? a. បាទ/ចាស់
   b. តទ

14. ត ើើអ្នកបកខ្លបាន**ជួយអ្នកឲ្យយល់ពី**អុ៣ំពីរតបៀបតគ្បីឱសែរបស់អ្នក ឬតទ ? a. បាទ/ចាស់ ពិ គ្សាកដតោើយ
   b. បាទ/ចាស់ មលះៗខ្លះ
   c. តទ

15. ត ើើអ្នកដឹងតទម អ្នកបកខ្លអាចបកខ្លការខុ នាំសគ្នាប់ការតគ្បីមន ៣ំតម តវជជបញ្ជជឬតទ ?
   a. បាទ/ចាស់
   b. តទ

16. កន ុងអុ៣ំឡុស ងតពល 12 ខ្នកនលងមកតនះ ត ើើអ្នកបានណា ់ជួប**ដោយគ្មា ន**អ្នកបកខ្លតទ ? a. បាទ/ចាស់
   b. តទ

17. ត ើើអ្នកណាមលះខ្លលបានជួយបកខ្លឲ្យអ្នកតៅតពលមិនមានអ្នកជាំនញបកខ្លជួយបកខ្ល ? សូ មរូសយក កយណាខ្លលជាប់ទាក់ទង ។
   a. មិ ៣ភកា៊ិ
   b. សមាជិកគ្រួសារ
   c. ប រាលិករលើនិក

18. ត ើការណា ់ជួបែែ៊ែៗរបស់អ្នកជាមួយអ្នកបកខ្លគ្សៀបតធៀបតៅនឹងការណា ់ជួប**ពីមុន** របស់អ្នកតដាយគ្មែ នអ្នកបកខ្ល ម សគ្នូនដូចតមាច ?
   a. លអ គ្បតសើរ
   b. គ្បខុៃលជាដូចគ្នន
   c. កាន់ ខុ មិនលអ

19. តលមមួយណាខ្លលអ្នកនឹងតគ្បីតដើមបីវ**ាយតម្លៃបទពិសោធន៍ថុ៣ៗបាំផុតរបស់់អ្នក**តៅ រលើនិក ? តគ្បីតលមណាមួយពី 0 ដល់ 10 ខ្លលតលម 0 វីជាបទពិតសាធន៍រលើនិកដ៏អាគ្កក់ បាំស តោើយតលម 10

រឺជាបទពិតសូធន៍រលើនិកដ៍លអបាំស ខ្ពលអាចតកើ មានតៅរលើនិក។ a. 0 បទពិតសូធន៍មិនលអបាំស ខ្ពលអាចតកើ មានតៅរលើនិក

b. 1
c. 2
d. 3
e. 4
f. 5
g. 6
h. 7
i. 8
j. 9
k. 10 បទពិតសូធន៍លអបាំស ខ្ពលអាចតកើ មានតៅរលើនិក

20. ត េើតភទរបស់អ្នករឺជាអ្ ៣ ី?
a. ក្បុ សស
b. ក្សី
c. ភាសូតសូៗ

21. ត េើអ្នកនិយាយភាសូអ៉ូតរលសលអប ណា ៣ខ្ពរ ?
a. លអ ណាស់
b. លអ
c. មិនលអ
d. មិនលអទាល់ ខ្ តសូះ

22. ត េើអ្នកវាយ ថមលចាំត ះស មភាពទូតៅរបស់អ្នកដូចតមាច ?
a. លអ បាំស
b. លអណាស់
c. លអ
d. មធយម
e. មិនលអ

សូ មអ្ររ ថាំត ះការចាំណាយតពលចូលរ ួមកន ុងការសិកាត្សវត្ជាវតនះ ។ តយើងនឹងតសញ្ញើជូនអ្នកនូវ កា អ្ ៣ ំតណាយតអ្ិចត្ ូនិច $10 តៅកាន់**អាសយដា ៣ នអ្ ុ ីណមលរបស់ ំអ្នក ឬដៅកាន់ទូរស ័ពទម្ របស់ ំអ្នក**តមរយៈអ្ ថបទកន ុងរយៈតពល 48 តមា ងបនា ប់ ។ សូ មបញ្ជាក់អ្ ៣ ំពីវធិ ីវីសូតសាមួយណា ខ្ពលលអបាំស សត្មាប់អ្នក។

a) អ្ ីវីខ្មល
b) ៃ ិច ឬ តសញ្ញើសូរ

សូ មសរតសរអាសយដា ៣ នអ្ ីវីខ្មលរបស់អ្នកតដើមបីតសញ្ញើកា អ្ ៣ ំតណាយតអ្ិចត្

ូនិក $10 ? សូ មសរតសរតលមទូរស័ពាចល័ របស់អ្នកតដើមបីនឹងតសញ្ញើរកា

អ្ ៣ ំតណាយតអ្ិចត្ ូនិច $10 ?

ត ើអ្នកចាប់អារម្មណ៍កនុងការពិភាក្សាអំពីបទពិសោធន៍ ខ្ញុំទោះទាំងអស់ មកពីខ្ញុំ ខ្ញុំ ៗរបស់អ្នកឱ្យបាន លំអិត ជាមួយអ្នកសម្ភាសន៍តទ ?
តយើងនឹងសាល់**កាតអំណោយឌីជីថលប្រតូនិក $25** បន្ថែមសម្រាប់ ការចូលរួមរបស់អ្នកកនុងការសម្ភាសន៍ រយៈតពល 30 នាទីតដាយសមាវ័ ។

a) បាទ/ចាស់ ខ្ញុំចាប់អារម្មណ៍

b) តទ ខ្ញុំមិនចាប់អារម្មណ៍តទ ។

តដើម្បីជួយតរៀបចំ ខ្សែនការសម្រាប់ការសម្ភាសន៍ នតពលអនាគត

របស់អ្នក៖ ត ើ**ដលខទូរស័ព្ទ**ណា ខ្លលអ បាំស តដើម្បីទាក់ទងអ្នក ?

ត ើអុីជាអាសយដ្ឋាន នអុីណាមល**ដ៏លអ បាំស តដើម្បីទាក់ទងអ្នក ?

អ្នកស្រាវជ្រាវនឹងទូរស័ព្ទមកអ្នក កនុងរយៈតពលពីរសប្តាហ៍បន្ទាប់ កនុងអំឡុងតពលខ្លលដាំត ើរការសមស្រប សម្រាប់អ្នក ។ សូមតជ្រើសតរ ើស **ប្រប់ដពលដេលាណ ល អំពីដែើរការ** សម្រាប់កាលវភាគរបស់អ្នក ។ តពលតវលាត្រូវបានរយក្សាប់កនុង ំបន់សា ង់ដារបា ស ិទីេតា ិក (តពលតវលារដាកាលីេតា ័រនីញ) ។

a) ថ្ងៃតធាើការ (ច័នាដល់ស ក្រ) តមា ង 11 ក្ដឹកដល់ 2 រតសៀល

b) ថ្ងៃតធាើការ (ច័នាដល់ស ក្រ) ពីតមា ង 3 រតសៀលដល់ 6 លាៃ ច

c) ថ្ងៃតៅរ៍ 9 ក្ដឹក-1 ថ្ងៃរតសៀល

d) តពលតស្វៗតទៀត

ក្បសិនតបើអ្នកតជ្រើសតរ ើស "តស្វតទៀត" **ដតើម្ងៃណាខៃេះជាដពលដេលា** ខ្លលអបាំស សម្រាប់ កាលវភាគរបស់អ្នក ?

សូ មអរ ចាំត ះការចាប់អារម្មណ៍របស់អ្នកកនុងការចូលរួមកនុងការសម្ភាសន៍តនះ ។ សូ ម តជ្រើសតរ ើសកាលបរ ិតចេទ និងតពលតវលាពី**ប្រប្រតិទិនដនេះ** តដើម្បីតរៀបចំកាលវភាគរសម្ភាសន៍របស់ អ្នក ។ ស្វរបញ្ជាក់នឹងត្រូវបានតសញ្ញើតៅអ្នកម នតពលសម្ភាសន៍របស់អ្នក ។

ការបួ ប់៖

1) "ការសា ង់ម ិបានបួ ប់ តដាយស្វរអ្នកមិនយល់ក្លមចូលរួម ។ ក្បសិនតបើអ្នកតៅខ្ញុំ ចង់ចូលរួម សូ មសទ ុកកម្មវធិ ីវ ករកតមអុ ីនធឺ ិ របស់អ្នកតឡើងវញិ តដើម្បីចាប់តសាើមការសា ង់ម ិ តឡើងវញិ ។ ំ ភាជ ប់តៅកាន់សនលឹកព័ ៌មានការសិក្សាត្រូវបានរួមបួ ូលតៅ**ទីដនេះ**។"

2) "សូ មអរ សម្រាប់ការបាំតពញការសា ង់ម ិ និងសម្រាប់ការចាប់អារម្មណ៍របស់អ្នកកនុងការចូលរួមកនុងការសម្ភាសន៍តនះ ។ ម ិខ្លលមអរបស់អ្នកនឹងជួយខ្លលមអតសវាកម្មសម្រាប់ អ្នកជាំងដូចឹ ជាអ្នក ។ កាលបរ ិតចេទ និងតពលតវលាសម្ភាសន៍ពិ ក្សាកដ នឹងត្រូវបានបញ្ជាក់ ជាមួយអ្នកបន្ថែមតទៀត តៅថ្ងៃខាងមុខ ។ ំ ភាជ ប់តៅកាន់សនលឹកព័ ៌មានការសិក្សាត្រូវបាន រួមបួ ូលតៅ**ទីដនេះ**។"

3) "សូ មអរ្រ សគ្មាប់ការបោំតពញការសា ង់ម ិអ្នុិនខ្ទរខុ តនះ ។ ការត្លើយ បរបស់អ្នកនឹង ជួយខ្ពុលមអតសវាកមែសគ្មាប់អ្នកជាំងដូចឺ ជាអ្នក ។ ៣ំ ភាជ ប់អ្នុិនតទរខុ តៅកាន់សនលឹក ព័ ័មានការសិកាត្សរគ្លារគុ ូវបានរ ួមបួ ូលតៅ**<u>ទីងនេះ</u>**។”

Lao

ແບບສຳຫຼວດຄົນເຈັບ

ທ່ານເວົ້າ**ພາສາ**ຫຍັງຢູ່ເຮືອນ?

ພາສາອາຫຼັບ
ພາສາອາເມເນຍ
ພາສາມຽນມາ
ພາສາກຳປູເຈຍ (ຂະເໝນກາງ)
ພາສາຈີນ (ຈີນກວາງຕຸ້ງ)
ພາສາຈີນ (ຈີນກາງ)
ພາສາດາຣີ
ພາສາຟາຊີ
ພາສາເຮຕິ ຄຣີໂອ
ພາສາຮິນດີ
ພາສາມົ້ງ
ພາສາຍີ່ປຸ່ນ
ພາສາກະຫຼ່ຽງ
ພາສາເກົາຫຼີ
ພາສາລາວ
ພາສາໂອໂຣໂມ
ພາສພາສໂຕ
ພາສາໂປຕຸເກດ
ພາສາປັນຈາບ
ພາສາລັດເຊຍ
ພາສາໂຊມາລີ
ພາສາສະເປນ
ພາສາສະວາຮີລີ
ພາສາຕາກາລັອກ
ພາສາໄທ
ພາສາທິກຣິນຍາ
ພາສາຢູເຄຣນ
ພາສາຫວຽດ
ພາສາອື່ນ

1. ວັນເດືອນປີເກີດຂອງທ່ານ:

2. ວັນທີ່ທ່ານມີນັດໝາຍກັບຄລີນິກຄັ້ງຫຼ້າສຸດ:

3. ຜູ້ໃຫ້ບໍລິການໄດ້**ຟັງທ່ານຢ່າງຕັ້ງໃຈ**ບໍ?
   a. ແມ່ນແລ້ວ, ແນ່ນອນ
   b. ແມ່ນແລ້ວ, ຄ່ອນຂ້າງຈະ
   c. ບໍ່

4. ຜູ້ໃຫ້ບໍລິການໄດ້**ສະແດງຄວາມເຄົາລົບ**ຕໍ່ສິ່ງທີ່ທ່ານເວົ້າບໍ?
   a. ແມ່ນແລ້ວ, ແນ່ນອນ
   b. ແມ່ນແລ້ວ, ຄ່ອນຂ້າງຈະ
   c. ບໍ່

5. ຜູ້ໃຫ້ບໍລິການໄດ້**ຊຸກຍູ້ໃຫ້ທ່ານຖາມຄຳຖາມ**ບໍ?
   a. ແມ່ນແລ້ວ, ແນ່ນອນ
   b. ແມ່ນແລ້ວ, ຄ່ອນຂ້າງຈະ
   c. ບໍ່

6. ຜູ້ໃຫ້ບໍລິການໄດ້**ໃຊ້ເວລາກັບທ່ານຢ່າງພຽງພໍ**ບໍ?

a. ແມ່ນແລ້ວ, ແນ່ນອນ
b. ແມ່ນແລ້ວ, ຄ່ອນຂ້າງຈະ
c. ບໍ່

7. ນາຍພາສາໄດ້**ຊ່ວຍທ່ານອະທິບາຍ**ຄວາມຮູ້ສຶກຂອງທ່ານໃຫ້ຜູ້ໃຫ້ບໍລິການຟັງບໍ?
    a. ແມ່ນແລ້ວ, ແນ່ນອນ
    b. ແມ່ນແລ້ວ, ຄ່ອນຂ້າງຈະ
    c. ບໍ່

8. ນາຍພາສາໄດ້**ຊ່ວຍໃຫ້ທ່ານເຂົ້າໃຈຄໍາແນະນໍາ**ຈາກຜູ້ໃຫ້ບໍລິການບໍ?
    a. ແມ່ນແລ້ວ, ແນ່ນອນ
    b. ແມ່ນແລ້ວ, ຄ່ອນຂ້າງຈະ
    c. ບໍ່

9. ນາຍພາສາໄດ້ປະຕິບັດຕໍ່ທ່ານດ້ວຍ**ຄວາມສຸພາບແລະຄວາມເຄົາລົບ**ບໍ?
    a. ແມ່ນແລ້ວ, ແນ່ນອນ
    b. ແມ່ນແລ້ວ, ຄ່ອນຂ້າງຈະ
    c. ບໍ່

10. ໃຊ້ຕົວເລກໃດກໍ່ໄດ້ຈາກ 0 ຫາ 10 ເຊິ່ງເລກ 0 ໝາຍເຖິງນາຍພາສາທີ່ບໍ່ເກັ່ງທີ່ສຸດເທົ່າທີ່ເປັນໄປໄດ້ ແລະເລກ 10 ໝາຍເຖິງນາຍພາສາທີ່ເກັ່ງທີ່ສຸດເທົ່າທີ່ເປັນໄປໄດ້, **ທ່ານຈະໃຊ້ຕົວເລກໃດໃນການໃຫ້ຄະແນນນາຍພາສາຜູ້ນີ້**?
    a. 0 ນາຍພາສາທີ່ບໍ່ເກັ່ງທີ່ສຸດເທົ່າທີ່ເປັນໄປໄດ້
    b. 1
    c. 2
    d. 3
    e. 4
    f. 5
    g. 6
    h. 7
    i. 8
    j. 9
    k. 10 ນາຍພາສາທີ່ເກັ່ງທີ່ສຸດເທົ່າທີ່ເປັນໄປໄດ້

11. ຜູ້ໃຫ້ບໍລິການໄດ້ຂໍໃຫ້ທ່ານກັບຄືນມາເພື່ອກວດກາຕິດຕາມຜົນບໍ?
    a. ແມ່ນ
    b. ບໍ່ແມ່ນ

12. ມີການສະໜອງນາຍພາສາໃຫ້ໃນການນັດໝາຍເພື່ອກວດກາຕິດຕາມຜົນບໍ?
    a. ແມ່ນ
    b. ບໍ່ແມ່ນ
    c. ຂ້າພະເຈົ້າບໍ່ຮູ້

13. ຜູ້ໃຫ້ບໍລິການໄດ້ແຈ້ງໃຫ້ທ່ານໄປຮັບຢາຕື່ມຕາມໃບສັ່ງຂອງແພດບໍ?
    a. ແມ່ນ
    b. ບໍ່ແມ່ນ

14. ນາຍພາສາໄດ້**ຊ່ວຍໃຫ້ທ່ານເຂົ້າໃຈ**ວິທີການໃຊ້ຢາບໍ?
    a. ແມ່ນແລ້ວ, ແນ່ນອນ
    b. ແມ່ນແລ້ວ, ຄ່ອນຂ້າງຈະ
    c. ບໍ່ແມ່ນ

15. ທ່ານຮູ້ບໍວ່ານາຍພາສາສາມາດແປຄໍາແນະນໍາໃນການໃຊ້ຢາຕາມໃບສັ່ງຂອງແພດໄດ້?
    a. ແມ່ນ
    b. ບໍ່ແມ່ນ

16. ໃນລະຫວ່າງ 12 ທີ່ຜ່ານມາ ທ່ານໄດ້ໄປຕາມນັດໝາຍ**ໂດຍບໍ່ມີ**ນາຍພາສາບໍ?
    a. ແມ່ນ
    b. ບໍ່ແມ່ນ

17. ບຸກຄົນໃດຊ່ອຍແປພາສາໃຫ້ທ່ານເວລາທີ່ທ່ານບໍ່ມີນາຍພາສາ? ໝາຍທຸກຂໍ້ທີ່ແມ່ນ.
    a. ໝູ່ເພື່ອນ
    b. ສະມາຊິກໃນຄອບຄົວ
    c. ພະນັກງານຂອງຄລີນິກ

18. ການນັດໝາຍຄັ້ງຫຼ້າສຸດທີ່ມີນາຍພາສາເປັນແນວໃດເມື່ອປຽບທຽບກັບການນັດໝາຍຂອງທ່ານ**ທີ່ຜ່ານມາ** ທີ່ບໍ່ມີນາຍພາສາ?
    a. ດີຂຶ້ນ
    b. ພໍປານກັນຄືເກົ່າ
    c. ຮ້າຍກວ່າເກົ່າ

19. ທ່ານຈະໃຊ້ຕົວເລກໃດໃນການ**ປະເມີນປະສົບການຄັ້ງຫຼ້າສຸດຂອງທ່ານ**ທີ່ຄລີນິກ? ໃຊ້ຕົວເລກໃດກໍ່ໄດ້ຈາກ 0 ຫາ 10 ເຊິ່ງເລກ 0 ໝາຍເຖິງປະສົບການທີ່ບໍ່ດີທີ່ສຸດເທົ່າທີ່ຈະເປັນໄປໄດ້ ແລະເລກ 10 ໝາຍເຖິງປະສົບການທີ່ດີທີ່ສຸດເທົ່າທີ່ຈະເປັນໄປໄດ້.
    a. 0 ປະສົບການທີ່ບໍ່ດີທີ່ສຸດເທົ່າທີ່ຈະເປັນໄປໄດ້
    b. 1
    c. 2
    d. 3
    e. 4
    f. 5
    g. 6
    h. 7
    i. 8
    j. 9
    k. 10 ປະສົບການທີ່ດີທີ່ສຸດເທົ່າທີ່ຈະເປັນໄປໄດ້

20. ທ່ານມີເພດຫຍັງ?
    a. ເພດຊາຍ
    b. ເພດຍິງ
    c. ອື່ນໆ

21. ທ່ານເວົ້າພາສາອັງກິດໄດ້ດີປານໃດ?
    a. ດີຫຼາຍ
    b. ດີ
    c. ບໍ່ດີປານໃດ
    d. ບໍ່ໄດ້ເລີຍ

22. ທ່ານຈະໃຫ້ຄະແນະສຸຂະພາບໂດຍລວມຂອງທ່ານວ່າດີປານໃດ?
    a. ດີເລີດ
    b. ດີຫຼາຍ
    c. ດີ
    d. ປານກາງ
    e. ບໍ່ດີ

ຂໍຂອບໃຈທີ່ທ່ານໃຊ້ເວລາໃນການເຂົ້າຮ່ວມໃນການວິໄຈສຶກສານີ້. ພວກເຮົາຈະສົ່ງບັດຂອງຂວັນເອເລັກໂຕຣນິກມູນຄ່າ 10 ໂດລາໄປທີ່**ອີເມວຂອງທ່ານ ຫຼື ໄປທີ່ໂທລະສັບມືຖືຂອງທ່ານທາງຂໍ້ຄວາມສັ້ນ** ພາຍໃນ 48 ຊົ່ວໂມງຂ້າງໜ້າ. ກະລຸນາລະບຸວິທີທີ່ທ່ານສະດວກທີ່ສຸດ.
a) ອີເມວ
b) ຂໍ້ຄວາມ

ທີ່ຢູ່ອີເມວສຳລັບສົ່ງບັດຂອງຂວັນເອເລັກໂຕຣນິກມູນຄ່າ 10 ໂດລາໄປໃຫ້ທ່ານແມ່ນຫຍັງ?

ເບີໂທລະສັບມືຖືຂອງທ່ານສຳລັບສົ່ງບັດຂອງຂວັນເອເລັກໂຕຣນິກມູນຄ່າ 10 ໂດລາໄປໃຫ້ທ່ານແມ່ນຫຍັງ?

ທ່ານມີຄວາມສົນໃຈໃນການສົນທະນາໃນລາຍລະອຽດກັບຜູ້ສໍາພາດກ່ຽວກັບປະສົບການໃນການດູແລຮັກສາສຸຂະພາບຂອງທ່ານຄັ້ງຫຼ້າສຸດບໍ່?
ພວກເຮົາຈະມອບບັດຂອງຂວັນເອເລັກໂຕຣນິກເພີ່ມເຕີມມູນຄ່າ 25 ໂດລາສໍາລັບການເຂົ້າຮ່ວມຂອງທ່ານໃນການສໍາພາດທີ່ເປັນຄວາມລັບ.

a) ແມ່ນແລ້ວ, ຂ້າພະເຈົ້າສົນໃຈ
b) ບໍ່, ຂ້າພະເຈົ້າບໍ່ສົນໃຈ

ເພື່ອຊ່ວຍໃນການວາງແຜນສໍາລັບການສໍາພາດທີ່ຈະມາເຖິງຂອງທ່ານ:

ເບີໂທລະສັບທີ່ສາມາດຕິດຕໍ່ທ່ານໄດ້ສະດວກທີ່ສຸດແມ່ນເບີຫຍັງ?

ທີ່ຢູ່ອີເມວທີ່ສາມາດຕິດຕໍ່ທ່ານໄດ້ສະດວກທີ່ສຸດແມ່ນຫຍັງ?

ນັກວິໄຈຈະໂທຫາທ່ານພາຍໃນສອງອາທິດຂ້າງໜ້າໃນເວລາທີ່ທ່ານສະດວກ. ກະລຸນາເລືອກ**ຊ່ວງເວລາທີ່ສະດວກ**ສໍາລັບທ່ານ.
ເວລາທີ່ລະບຸໄວ້ແມ່ນເວລາມາດຕະຖານເຂດປາຊີຟິກ (ເວລາຄາລິຟໍເນຍ).

a) ວັນທໍາການ (ວັນຈັນເຖິງວັນສຸກ) 11 ໂມງເຊົ້າ - 2 ໂມງບ່າຍ
b) ວັນທໍາການ (ວັນຈັນເຖິງວັນສຸກ) 3 ໂມງບ່າຍ - 6 ໂມງແລງ
c) ວັນເສົາ 9 ໂມງເຊົ້າ -1 ໂມງບ່າຍ
d) ວັນແລະເວລາອື່ນ

ຖ້າທ່ານເລືອກ "ວັນແລະເວລາອື່ນ", **ວັນແລະເວລາໃດ**ທີ່ທ່ານສະດວກທີ່ສຸດ?

ຂໍຂອບໃຈທີ່ທ່ານສົນໃຈໃນການເຂົ້າຮ່ວມໃນການສໍາພາດນີ້.
ກະລຸນາເລືອກວັນທີແລະເວລາຈາກ**ປະຕິທິນນີ້**ເພື່ອນັດໝາຍການສໍາພາດຂອງທ່ານ.
ຂໍ້ຄວາມຢັ້ງຢືນຈະຖືກສົ່ງໄປຫາທ່ານກ່ອນຈະມີການສໍາພາດກັບທ່ານ.

ຂໍ້ຄວາມຕອນຈົບ:
1) "ການສໍາຫຼວດໄດ້ສິ້ນສຸດລົງເນື່ອງຈາກທ່ານບໍ່ຍິນຍອມທີ່ຈະເຂົ້າຮ່ວມ. ຖ້າທ່ານຍັງຕ້ອງການເຂົ້າຮ່ວມ
ກະລຸນາໂຫຼດໜ້າຈໍຕົວທ່ອງເວັບຄືນໃໝ່ (refresh) ເພື່ອເລີ່ມຕົ້ນການສໍາຫຼວດໃໝ່.
ລິ້ງເຊື່ອມຕໍ່ກັບເອກະສານຂໍ້ມູນການວິໄຈສຶກສານີ້ແມ່ນລວມຢູ່**ທີ່ນີ້**."

2) "ຂໍຂອບໃຈທີ່ທ່ານໃຊ້ເວລາໃນການເຮັດແບບສໍາຫຼວດນີ້ ແລະທີ່ທ່ານສົນໃຈໃນການເຂົ້າຮ່ວມໃນການສໍາພາດ.
ຄໍາຕິຊົມຂອງທ່ານຈະຊ່ວຍປັບປຸງບໍລິການສໍາລັບຄົນເຈັບເຊັ່ນທ່ານ. ຈະມີການຢັ້ງຢືນວັນທີແລະເວລາໃນການສໍາພາດກັບທ່ານໃນມື້ຕໍ່ໄປ.
ລິ້ງເຊື່ອມຕໍ່ກັບເອກະສານຂໍ້ມູນການວິໄຈສຶກສານີ້ແມ່ນລວມຢູ່**ທີ່ນີ້**."

3) "ຂໍຂອບໃຈທີ່ທ່ານໄດ້ເຮັດແບບສໍາຫຼວດນີ້. ຄໍາຕອບຈະຊ່ວຍປັບປຸງບໍລິການສໍາລັບຄົນເຈັບເຊັ່ນທ່ານ.
ລິ້ງເຊື່ອມຕໍ່ກັບເອກະສານຂໍ້ມູນການວິໄຈສຶກສານີ້ແມ່ນລວມຢູ່**ທີ່ນີ້**."

Oromo

Qorannoo Dhukkubsataa

Manatti **afaan** kam dubbatta?

Arabiffa
Afaan Armeeniyaa
Burmeese
Kamboodiyaa (Kehmer Giddugalaa)
Afaan Chaayinaa (Kantonesee)
Afaan Chaayinaa (Mandariinii)
Daarii
Farsii
Haayitaan Kiri'oollee
Hindii
Hamong
Afaan Jaappaan
Kaaren
Afaan Kooriyaa
Laa'oshiyaanii
Afaan Oromoo
Pashtoo
Afaan Porchugiiz
Punjaabii
Afaan Raashiyaa
Afaan Somaalii
Afaan Ispaanish
Iswaahilii
Taagaloog
Taayii
Afaan Tigriffaa
Afaan Yukireen
Afaan Viyeetnaam
Kan biro

1. Guyyaa dhaloota kee:

2. Guyyaa beellama kilinikaa kee kan dhiyeenyaa:

3. Ogeessi fayyaa **hubannoodhaan si dhaggeeffateeraa**?
    a. Eeyyeen, guutummaatti
    b. Eeyyeen, hamma tokko
    c. Lakki

4. Ogeessi fayyaa waan ati jechaa turteef **kabaja kennaa** turee?
    a. Eeyyeen, guutummaatti
    b. Eeyyeen, hamma tokko
    c. Lakki

5. Ogeessi fayyaa **akka gaaffii gaafattu si jajjabeesseeraa**?
    a. Eeyyeen, guutummaatti

b. Eeyyeen, hamma tokko
c. Lakki

6. Ogeessi fayyaa **yeroo gahaa tahe si wajjin** dabarseeraa?
    a. Eeyyeen, guutummaatti
    b. Eeyyeen, hamma tokko
    c. Lakki

7. Namni afaan hiiku waan sitti dhagahamaa ture **ogeessa fayyaatif sirriitti**ibseeraa?
    a. Eeyyeen, guutummaatti
    b. Eeyyeen, hamma tokko
    c. Lakki

8. Namni afaan hiiku **gorsa ogeessa fayyaa akka ati hubbattu** si gargaareraa?
    a. Eeyyeen, guutummaatti
    b. Eeyyeen, hamma tokko
    c. Lakki

9. Namni afaan hiiku **afaan ho'aa fi kabajaan**si tajaajileeraa?
    a. Eeyyeen, guutummaatti
    b. Eeyyeen, hamma tokko
    c. Lakki

10. Lakkoofsa 0 irraa haga 10 jiru fayyadamuudhan, 0 namni afaan hiiku baay'ee dadhabaa fi 10 ammoo baay'ee cimaa ta'uu isaa, **nama afaan hiiku kana ibsuudhaf lakkoofsa kam fayyadamta**?
    a. 0 nama afaan hiiku baay'ee dadhabaa ta'e
    b. 1
    c. 2
    d. 3
    e. 4
    f. 5
    g. 6
    h. 7
    i. 8
    j. 9
    k. 10 nama afaan hiiku baay'ee cimaa ta'e

11. Ogeessi fayyaa beellama hordoffiif deebitee akka dhuftu si gaafateeraa?
    a. Eeyyee
    b. Lakki

12. Beellama hordoffii keef turjumaanni/namni afaan hiiku qophaa'eeraa?
    a. Eeyyee
    b. Lakki
    c. Ani hin beeku

13. Ogeessi fayyaa qorichaa sii ajajee akka guuttattu sitti himeeraa?
    a. Eeyyee
    b. Lakki

14. Turjumaanni akkamittiin akka qorichi fudhatamu **akka hubattuuf si gargaareeraa**?

a. Eeyyeen, guutummaatti
b. Eeyyeen, hamma tokko
c. Lakki

15. Turjumaanni qajeelfamoota akkaataa ittiin qoricha ajajame fudhatan akka afaan keetti hiikuu danda'u ni beekta turee?
    a. Eeyyee
    b. Lakki

16. Ji'oota 12'n darban keessa turjumaana **malee** beellama qabattee beektaa?
    a. Eeyyee
    b. Lakki

17. Yeroo ati turjumaana hin qabaanne afaan sii hiike? Kan ilaallatu hundatti mallattoo godhi.
    a. Hiriyaa
    b. Miseensa maatii
    c. Hojjetaa kilinikaa

18. Beellamni kee kan dhiyeenyaa turjumaanni si wajjin ture kan isa **duraanii** turjumaana malee ture wajjin yoo wal bira qabdu maal fakkaata?
    a. Fooyya'aa
    b. Wal fakkaataa
    c. Badaa

19. **Turtii kee dhiyeenyaa kilinikatti taasifte ibsuuf** lakkoofsa kam fayyadamta? Lakkoofsa 0 haga 10 tti dhimma ba'ii, kan 0'n turtii kilinikaa baayyee badaa tahe fi 10 turtii kilinikaa baayyee gaarii tahe.
    a. 0 Turtii baayyee badaa tahe
    b. 1
    c. 2
    d. 3
    e. 4
    f. 5
    g. 6
    h. 7
    i. 8
    j. 9
    k. 10 Turtii baayyee gaari tahe

20. Saalli kee maal?
    a. Dhiira
    b. Dhalaa
    c. Kan biro

21. Ingiliffa hagam bareechitee dubbatta?
    a. Baayyee gaarii
    b. Gaarii
    c. Baayyee gaarii miti
    d. Tasuma

22. Fayyaa kee waliigalaa akkamiin madaalta?
    a. Baayyee baayyee gaarii

b. Baayyee gaarii
c. Gaarii
d. Fooyya'aa
e. Gadaanaa

Yeroo fudhattee qorannoo kana irratti waan hirmaattef galatoomi. Sa'atii 48 itti aanan keessatti kaardii kennaa elektiroonikaa $10 karaa **teessoo iimelii keetii yookaan barreeffamaan bilbila keetti** siif ergina. Karaa kam akka sii wayyu filadhu.

a) Iimelii (ergaa elektiroonikaa)
b) Ergaa barreeffama gabaabaa bilbilaa

Kaardii kennaa elektiroonawaa $10 sii erguuf teessoon iimelii kee maalidha?

Kaardii kennaa elektiroonawaa $10 sii erguuf lakkoofsi bilbilaakee maalidha?

Turtii waldhaansa fayyaakee yeroo dhiyoo gadi fageenyaan nama afgaaffii si gaafatu waliin dubbachuuf fedhii qabdaa? Afgaaffii daqiiqaa 30 iccitiin isaa eegame yoo hirmaatte **kaardii kennaa elektiroonawaa $25** dabalataa sii kennina.

a) Eeyyee, fedhii qaba
b) Lakki, fedhii hin qabu

Afgaaffiikee dhufu karoorsuuf akka si gargaarutti:

Lakkoofsi bilbilaa mijataan ittiin si quunnamnu kami?

Teessoon iimelii mijataan ittiin si quunnamnu kami?

Torban lamaan dhufu keessa yeroo sii mijatutti qorataan tokko siif bilbila. Maaloo **sa'atii sagantaa keewajjin mijaa'uchuu hunda** filadhu. Sa'atiin akka Yeroo Durtii Pasifik (Yeroo Kaliforniyaa) tti kaa'ameera.

a) Guyyoota torbanii (Wiixataa hanga Jimaataa) 11AM-2PM
b) Guyyoota torbanii (Wiixataa hanga Jimaataa) 3PM-6PM
c) Sanbata 9AM-1PM
d) Kan biro

Yoo "kan biro" isa jedhu filatte, **guyyoota torbanii fi sa'atiiwwan kamtu** sagantaa kee kanaaf baayyee siif mijata?

Afgaaffii kana keessatti hirmaachuuf fedhii qabaachuu keef galatoomi. Afgaaffii kee saganteessuuf maaloo **kaleendera kana** keessaa guyyaa fi sa'atii filadhu. Afgaaffii kee dura ergaan mirkaneeffannaa siif ergama.

Xumura:

1) "Qorannoon kun sababa ati itti hirmaachuuf fedha hin qabneef xumurameera. Yoo ammayyuu itti hirmaachuu ni barbaadda tahe, maaloo irra deebiin qorannicha jalqabuuf browsericha (iyyaafata) haaromsi (refresh godhi). Linkiin gara waraqaa ideeffannoo qorannichaa geessu **as jira**."

2) "Guca qorannoo kana waan guutteef akkasumas afgaaffii kanatti hirmaachuuf fedhii waan qabduuf galatoomi. Deebiinkee dhukkubsatoota akka keef tajaajila fooyyessuuf gargaara. Guyyaanii fi sa'atiin afgaaffii kee inni murtaa'e guyyoota as deeman keessa si waliin mirkana'a. Linkiin (Korinyoo) gara waraqaa odeeffannoo qorannichaa geessu **asitti**dabalameera."

3) “Qorannoo kanatti waan hirmaatteef galatoomi. Deebiin kee dhukkubsataa akka keef tajaajila fooyyessuuf gargaara. Linkiin gara waraqaa odeeffannoo qorannichaa geessu **asitti**dabalameera.”

Pashto

د ناروغ نظرپوښتنه

تاسو په کور کې په کومه ژبه خبرې کوئ؟

عربي
ارمنيايي
برمايي
کمبوډيايي (مرکزي خمېر)
چينايي (کانتونيز)
چينايي (ماندارين)
درى
فارسي
هايتي کريول
هندي
هامونګ
جاپاني
کرين
کوريايي
ليوتين
اورومو
پښتو
پورتګالي
پنجابي
روسي
سومالي
هسپانوي
سواحلي
تګالوګ
تهايي
تيګرينيايي
يوکرايني
وېتنامي
بله ژبه

1. ستاسو د زېږېدو نېټه:

2. ستاسو د ترټولو وروستۍ کلينيکي ليدني نېټه:

3. ايا د روغتيايي خدماتو چمتو کوونکي ستاسو خبرې په غور سره واورېدلې؟
   a. هو، بالکل
   b. هو، يوه اندازه
   c. نه

4. ايا د روغتيايي خدماتو چمتو کوونکي هغه څه ته چې تاسو ويل په درناوي سره وکتل؟
   a. هو، بالکل
   b. هو، يوه اندازه
   c. نه

5. ایا د روغتیایي خدماتو چمتو کوونکي تاسو پوښتنو کولو ته وهڅولئ؟
   a. هو، بالکل
   b. هو، یوه اندازه
   c. نه

6. ایا د روغتیایي خدماتو چمتو کوونکي له تاسو سره کافي وخت خبرې وکړې؟
   a. هو، بالکل
   b. هو، یوه اندازه
   c. نه

7. ایا شفاهي ژباړونکي له تاسو سره د روغتیایي خدماتو چمتو کوونکي ته د هغه څه په تشریح کولو کې مرسته وکړه چې تاسو څه ډول احساس درلوده؟
   a. هو، بالکل
   b. هو، یوه اندازه
   c. نه

8. ایا شفاهي ژباړونکي له تاسو سره د روغتیایي خدماتو د چمتو کوونکي له لوري د لارښوونو په پوهېدو کې مرسته وکړه؟
   a. هو، بالکل
   b. هو، یوه اندازه
   c. نه

9. ایا شفاهي ژباړونکي له تاسو سره په درناوي او ادب سره چلند وکړ؟
   a. هو، بالکل
   b. هو، یوه اندازه
   c. نه

10. د 0 څخه تر 10 پورې د یوې شمېرې کارول، چېرته چې 0 د امکان تر حده تر ټولو ناوړه شفاهي ژباړونکی ؤ او 10 د امکان تر حده تر ټولو ښه شفاهي ژباړونکی ؤ، تاسو به دغې شفاهي ژباړونکي ته د نمرې ورکولو لپاره کومه شمېره کاروئ؟
   a. 0 د امکان تر حده تر ټولو ناوړه شفاهي ژباړن
   b. 1
   c. 2
   d. 3
   e. 4
   f. 5
   g. 6
   h. 7
   i. 8
   j. 9
   k. 10 د امکان تر حده تر ټولو ښه شفاهي ژباړن

11. ایا د روغتیایي خدماتو چمتو کوونکي تاسو ته وویل چې د یوې بلې لیدنې لپاره بیا راشئ؟
   a. هو
   b. نه

12. ایا ستاسو د بلې لیدنې لپاره درته یو شفاهي ژباړونکی حاضر شوی ؤ؟
   a. هو
   b. نه
   c. نه پوهېږم

13. ایا د روغتیایي خدماتو چمتو کوونکي تاسو ته وویل چې د درملو لپاره یوه نسخه ډکه کړئ؟

a. هو
b. نه

14. ايا شفاهي ژباړونکي له تاسو سره مرسته وکړه چې پوه شئ چې خپل درمل باید څه ډول وخوړئ؟
a. هو، بالکل
b. هو، یوه اندازه
c. نه

15. ايا تاسو پوهېږئ چې يو شفاهي ژباړونکی کولی شي د درکړل شويو درملو د خوړلو لپاره لارښوونې وژباړي؟
a. هو
b. نه

16. په تېرو 12 مياشتو کې، ايا تاسو پرته له يوه شفاهي ژباړونکي څخه کومه ليدنه درلودلې ده؟
a. هو
b. نه

17. کله چې تاسو يوه شفاهي ژباړونکی نه درلود نو چا درسره د ژباړې په برخه کې مرسته وکړه؟ ټول هغه په نښه کړئ چې ستاسو لپاره صدق کوي.
a. ملګری
b. د کورنۍ غړی
c. د کلينيک کارکوونکي

18. ستاسو وروستۍ ليدنه چې يوه طبي ژباړونکي پکې شتون درلود ستاسو د پخوانيو هغو سره چې ژباړونکي پکې شتون نه درلود څه ډول پرتله کوئ؟
a. ورڅخه غوره
b. تقريباً يو شان
c. تر هغې بده

19. تاسو به په کلينيک کې د خپلې تر ټولو وروستۍ تجربې د درجه بندي کولو لپاره کومه شمېره وکاروئ؟ له 0 څخه تر 10 پورې کومه یوه شمېره وکاروئ، چې 0 د امکان تر حده تر ټولو ناوړه کلينيکي تجربه ده او 10 د امکان تر حده تر ټولو غوره کلينيکي تجربه ده.
a. 0 د امکان تر حده تر ټولو ناوړه تجربه
b. 1
c. 2
d. 3
e. 4
f. 5
g. 6
h. 7
i. 8
j. 9
k. 10 د امکان تر حده تر ټولو غوره تجربه

20. ستاسو جنسيت کوم ډول دی؟
a. نارينه
b. ښځينه
c. بله ژبه

21. تاسو په انګليسي ژبه څومره ښې خبرې کولی شئ؟
a. ډېرې ښې
b. ښې
c. ښې نه

d. هيڅ نه

22. تاسو خپله عمومي روغتيا څه ډول ارزوئ؟
a. فوق العاده
b. ډېره ښه
c. ښه
d. مناسبه
e. بده

په دغه څېړنه کې د ګډون لپاره له وخت ورکولو لپاره له تاسو څخه مننه کوو. مونږ به په راتلونکو 48 ساعتو کې ستاسو بریښنالیک پتې ته یا د لنډ لیکلي پیغام له لارې ستاسو ګرځنده تلیفون ته د 10 امریکایي ډالرو په ارزښت یو بریښنایي ډالی کارت در ولېږو. مهرباني وکړئ په ګوته کړئ چې تاسو لپاره کومه طریقه غوره ده.
a) بریښنالیک
b) لنډ لیکلی پیغام

د 10 امریکایي ډالرو بریښنایي ډالی کارت د لیږلو لپاره ستاسو د بریښنالیک پته کومه ده؟

د 10 امریکایي ډالرو بریښنایي ډالی کارت د لیږلو لپاره ستاسو د ګرځنده تلیفون شمېره کومه ده؟

ایا تاسو علاقه لرئ چې له یوه مرکه کوونکي سره د خپلې روغتیایي پاملرنې د وروستي تجربې په اړه په ډېر تفصیل سره خبرې اترې وکړئ؟ مونږ به تاسو ته په یوه 30-دقیقې یي محرمه مرکه کې د ګډون لپاره د 25 امریکایي ډالرو په ارزښت بل بریښنایي ډالی کارت هم درکړو.
a) هو، زه يې علاقه لرم
b) نه، زه يې علاقه نه لرم

ستاسو د راتلونکې مرکې په پلان کولو کې د مرستې لپاره:

له تاسو سره اړیکې نیولو لپاره تر ټولو غوره تلیفون شمېره کومه ده؟

له تاسو سره د اړیکې نیولو لپاره تر ټولو غوره بریښنالیک پته کومه ده؟

یو څېړونکی به د راتلونکو دوو اوونیو په اوږدو کې تاسو ته په داسې وخت کې زنګ ووهي چې ستاسو لپاره مناسب وي. مهرباني وکړئ د وختونو هغه ټولې کړکۍ انتخاب کړئ چې ستاسو له مهالویش سره مناسبې دي. وختونه به د متحده ایالاتو د معیاري وخت (د کالیفورنیا د وخت) سره سم وي.
a) د اوونۍ کاري ورځې (له دوشنبې څخه تر جمعې پورې) د سهار 11 بجو څخه تر ماسپښین 2 بجو پورې
b) د اوونۍ کاري ورځې (له دوشنبې څخه تر جمعې پورې) د ماسپښین 3 بجو څخه تر ماښام 6 بجو پورې
c) شنبه د سهار 9 بجو څخه تر غرمې 1 بجې پورې
d) بله ژبه

که تاسو ''نور'' انتخاب کړئ، نو د اوونۍ کومې ورځې او کوم وختونه به ستاسو له مهالویش سره په ښه توګه سمون ولري؟

په یوه مرکه کې د ګډون لپاره ستاسو له علاقې لپاره له تاسو څخه مننه کوو. مهرباني وکړئ د خپلې مرکې د مهالویش کولو لپاره د دغې کلیزې (جنترۍ) څخه نېټه او وخت انتخاب کړئ. له خپلې مرکې څخه مخکې به تاسو ته د تایید یو لنډ لیکلی پیغام ولېږل شي.

پایلې:
1) "نظرپوښتنه پای ته ورسېده ځکه چې تاسو پکې د ګډون کولو رضایت و نه ښوده. که تاسو لا هم غواړئ چې پکې ګډون وکړئ، مهرباني وکړئ د نظرپوښتنې د بیا پیلولو لپاره خپل انټرنېټ لټونګر (براوزر) تازه کړئ. د څېړنې د معلوماتو د پاڼې یو لېنک يې دلته شتون لري. "

2) د نظرپوښتنې د بشپړولو او په یوه مرکه کې ګډون په اړه د علاقې ښودلو لپاره له تاسو څخه مننه کوو. ستاسو نظریې او وړاندیزونه به ستاسو په څېر ناروغانو لپاره د خدماتو په ښه والي کې مرسته وکړي. ستاسو د مرکې دقیقه نېټه او وخت به په راتلونکو ورځو کې ستاسو په مشوره وټاکل شي. د څېړنې د معلوماتو د پاڼې یو لېنک يې دلته شتون لري. "

3) "د نظرپوښتنې د بشپړولو لپاره تاسو مننه کوو. ستاسو ځوابونه به ستاسو په څېر ناروغانو لپاره د خدماتو په ښه والي کې مرسته وکړي. د څېړنې د معلوماتو د پاڼې یو لېنک یې دلته شتون لري. "

Portuguese

Pesquisa com pacientes

Qual **idioma** você fala em casa?

árabe
armênio
birmanês
cambojano (khmer central)
chinês (cantonês)
chinês (mandarim)
coreano
crioulo haitiano
dari
espanhol
hindi
hmong
japonês
karen
laosiano
oromo
panjábi
pashto
persa (farsi)
português
russo
somali
suaíli
tagalo
tailandês
tigrínia
ucraniano
vietnamita
Outro:

1. Sua data de nascimento:

2. Data da consulta mais recente na clínica:

3. O provedor de serviços **ouviu você atentamente**?
    a. Sim, definitivamente
    b. Sim, mais ou menos
    c. Não

4. O provedor de serviços **demonstrou respeito** pelo que tinha a dizer?
    a. Sim, definitivamente
    b. Sim, mais ou menos
    c. Não

5. O provedor de serviços **incentivou você a fazer perguntas**?
    a. Sim, definitivamente
    b. Sim, mais ou menos
    c. Não

6. O provedor de serviços **dedicou tempo suficiente** a você?
    a. Sim, definitivamente
    b. Sim, mais ou menos
    c. Não

7. O profissional de interpretação **ajudou você a explicar** para o provedor de serviços como você estava se sentindo?
    a. Sim, definitivamente
    b. Sim, mais ou menos
    c. Não

8. O profissional de interpretação **ajudou você a compreender as instruções** do provedor de serviços?
    a. Sim, definitivamente
    b. Sim, mais ou menos
    c. Não

9. O profissional de interpretação tratou você com **cortesia e respeito**?
    a. Sim, definitivamente
    b. Sim, mais ou menos
    c. Não

10. Usando um número de 0 a 10, sendo que 0 é a pior interpretação possível e 10 é a melhor interpretação possível, **qual nota você daria para o profissional de interpretação**?
    a. 0 pior interpretação possível
    b. 1
    c. 2
    d. 3
    e. 4
    f. 5
    g. 6
    h. 7
    i. 8
    j. 9
    k. 10 melhor interpretação possível
11. O provedor de serviços pediu para você voltar para uma consulta de acompanhamento?
    a. Sim
    b. Não

12. Agendaram o profissional de interpretação para a consulta de acompanhamento?
    a. Sim
    b. Não
    c. Não sei

13. O provedor de serviços disse que precisa obter medicamento com receita?
    a. Sim

    b. Não

14. O profissional de interpretação **ajudou você a compreender** como tomar o remédio?
    a. Sim, definitivamente
    b. Sim, mais ou menos
    c. Não

15. Você sabia que um profissional de interpretação pode traduzir as instruções sobre como tomar o remédio com receita?
    a. Sim
    b. Não

16. Durante os últimos 12 meses, você teve alguma consulta **sem** um profissional de interpretação?
    a. Sim
    b. Não

17. Quem ajudou a interpretar para você quando um profissional de interpretação não estava presente? Marque todas as opções válidas:
    a. Amigo
    b. Parente
    c. Equipe da clínica

18. Como a sua consulta mais recente se compara às consultas **passadas** sem a presença de um profissional de interpretação?
    a. Melhor
    b. Mais ou menos a mesma coisa
    c. Pior

19. Qual nota você daria para **a sua interação mais recente** na clínica? Use um número de 0 a 10, sendo que 0 seria a pior interação possível na clínica e 10 seria a melhor interação possível.
    a. 0 pior interação possível
    b. 1
    c. 2
    d. 3
    e. 4
    f. 5
    g. 6
    h. 7
    i. 8
    j. 9
    k. 10 melhor interação possível

20. Qual é o seu gênero?
    a. Masculino
    b. Feminino
    c. Outro

21. Você fala inglês bem?
    a. Muito bem
    b. Bem
    c. Não muito bem

    d. Nem um pouco

22. Como você classificaria a sua saúde em geral?
    a. Excelente
    b. Muito boa
    c. Bom
    d. Razoável
    e. Baixa

Agradecemos pelo tempo dedicado à participação neste estudo. Enviaremos um vale-presente eletrônico no valor de US$ 10 **para o seu e-mail ou por SMS** dentro das próximas 24 horas. Indique o método mais conveniente para você.

a) E-mail
b) SMS

Para qual endereço de e-mail devemos mandar o vale-presente eletrônico no valor de US$ 10?

Para qual número de telefone devemos mandar o vale-presente eletrônico no valor de US$ 10?

Você gostaria de conversar sobre a assistência médica recebida mais recentemente e entrar em detalhes com um entrevistador? Enviaremos outro **vale-presente eletrônico no valor de US$ 25** pela sua participação em uma entrevista de 30 minutos.

a) Sim, tenho interesse
b) Não, não tenho interesse

Para ajudar você a se planejar para a entrevista:

Qual é o melhor **número de telefone** para entrarmos em contato?

Qual é o melhor **endereço de e-mail** para entrarmos em contato?

Um pesquisador vai ligar para você nas próximas duas semanas durante um horário conveniente para você. Selecione **todos os horários convenientes** de acordo com a sua agenda. Os horários indicados são no fuso horário da Califórnia.

a) Dias de semana (segunda a sexta) das 11h às 14h
b) Dias de semana (segunda a sexta) das 15h às 18h
c) Sábados, das 9h às 13h
d) Outro

Se você escolher "outro", **quais dias da semana e horários** seriam mais convenientes de acordo com a sua agenda?

Agradecemos pelo seu interesse em participar da entrevista. Selecione uma data e um horário **neste calendário** para marcar a sua entrevista. Uma mensagem de confirmação será enviada para você antes da entrevista.

Encerramentos:
1) "A pesquisa foi encerrada porque você não concordou em participar. Se ainda quiser participar, atualize a página no seu navegador para recomeçar a pesquisa. Um link para a ficha de informação sobre a pesquisa está incluído **aqui**."

2) “Agradecemos por responder à pesquisa e pelo seu interesse em participar da entrevista. As suas opiniões serão usadas para ajudar a melhorar os serviços prestados a pacientes como você. A data e o horário da sua entrevista serão confirmados nos próximos dias. Um link para a ficha de informação sobre a pesquisa está incluído **aqui**.”

3) “Agradecemos por responder à pesquisa. As suas respostas serão usadas para ajudar a melhorar os serviços prestados a pacientes como você. Um link para a ficha de informação sobre a pesquisa está incluído **aqui**.”

Punjabi

ਮਰੀਜ਼ ਸਰਵੇਖਣ

ਤੁਸੀਂ ਘਰ ਵਿੱਚ ਕਿਹੜੀ **ਭਾਸ਼ਾ** ਬੋਲਦੇ ਹੋ?

ਅਰਬੀ
ਅਰਮੇਨਿਅਨ
ਬਰਮੀ
ਕੰਬੋਡੀਅਨ (ਕੇਂਦਰੀ ਖਮੇਰ)
ਚੀਨੀ (ਕੈਂਟੋਨੀਸ)
ਚੀਨੀ (ਮੈਂਡਾਰਿਨ)
ਡੈਰੀ
ਫਾਰਸੀ
ਹਾਈਤਿਨ ਕ੍ਰੇਓਲ
ਹਿੰਦੀ
ਹਮੌਂਗ
ਜਾਪਾਨੀ
ਕਰੇਨ
ਕੋਰਿਆਈ
ਲਾਓਤੀਅਨ
ਔਰੋਮੋ
ਪਾਸ਼ਤੋ
ਪੁਰਤਗਾਲੀ
ਪੰਜਾਬੀ
ਰੂਸੀ
ਸੋਮਾਲੀ
ਸਪੈਨਿਸ਼
ਸਵਾਹਿਲੀ
ਟੈਗਾਲੋਗ
ਥਾਈ
ਟਿਗਰੀਨੀਆ
ਯੁਕਰੇਨੀਅਨ
ਵਿਯਤਨਾਮੀ
ਕੋਈ ਹੋਰ

1. ਤੁਹਾਡੀ ਜਨਮ ਦੀ ਤਾਰੀਖ਼:

2. ਤੁਹਾਡੀ ਸਭ ਤੋਂ ਹਾਲ ਹੀ ਦੀ ਕਲੀਨਿਕ ਅਪੌਇੰਟਮੈਂਟ ਦੀ ਤਾਰੀਖ਼:

3. ਕੀ ਪ੍ਰਦਾਤੇ **ਨੇ ਧਿਆਨ ਨਾਲ ਤੁਹਾਡੀ ਗੱਲ ਸੁਣੀ?**

a. ਹਾਂ, ਨਿਸ਼ਚਤ ਤੌਰ ‘ਤੇ
b. ਹਾਂ, ਕੁਝ ਹੱਦ ਤੱਕ
c. ਨਹੀਂ

4. ਕੀ ਪ੍ਰਦਾਤੇ ਨੇ ਤੁਹਾਡੀਆਂ ਗੱਲਾਂ ਦੇ ਲਈ **ਆਦਰ ਦਿਖਾਇਆ**?
    a. ਹਾਂ, ਨਿਸ਼ਚਤ ਤੌਰ ‘ਤੇ
    b. ਹਾਂ, ਕੁਝ ਹੱਦ ਤੱਕ
    c. ਨਹੀਂ

5. ਕੀ ਪ੍ਰਦਾਤੇ ਨੇ **ਤੁਹਾਨੂੰ ਸਵਾਲ ਪੁੱਛਣ ਦੇ ਲਈ ਉਤਸ਼ਾਹਤ ਕੀਤਾ**?
    a. ਹਾਂ, ਨਿਸ਼ਚਤ ਤੌਰ ‘ਤੇ
    b. ਹਾਂ, ਕੁਝ ਹੱਦ ਤੱਕ
    c. ਨਹੀਂ

6. ਕੀ ਪ੍ਰਦਾਤੇ ਨੇ ਤੁਹਾਡੇ ਨਾਲ **ਕਾਫੀ ਸਮਾਂ ਬਿਤਾਇਆ**?
    a. ਹਾਂ, ਨਿਸ਼ਚਤ ਤੌਰ ‘ਤੇ
    b. ਹਾਂ, ਕੁਝ ਹੱਦ ਤੱਕ
    c. ਨਹੀਂ

7. ਕੀ ਦੁਭਾਸ਼ੀਏ ਨੇ ਤੁਹਾਡੀਆਂ ਭਾਵਨਾਵਾਂ ਨੂੰ ਪ੍ਰਦਾਤੇ ਨੂੰ **ਸਮਝਾਉਣ ਵਿੱਚ ਤੁਹਾਡੀ ਮਦਦ ਕੀਤੀ**?
    a. ਹਾਂ, ਨਿਸ਼ਚਤ ਤੌਰ ‘ਤੇ
    b. ਹਾਂ, ਕੁਝ ਹੱਦ ਤੱਕ
    c. ਨਹੀਂ

8. ਕੀ ਦੁਭਾਸ਼ੀਏ ਨੇ ਪ੍ਰਦਾਤੇ ਦੀਆਂ **ਹਿਦਾਇਤਾਂ ਨੂੰ ਸਮਝਣ ਵਿੱਚ ਤੁਹਾਡੀ ਮਦਦ** ਕੀਤੀ?
    a. ਹਾਂ, ਨਿਸ਼ਚਤ ਤੌਰ ‘ਤੇ
    b. ਹਾਂ, ਕੁਝ ਹੱਦ ਤੱਕ
    c. ਨਹੀਂ

9. ਕੀ ਦੁਭਾਸ਼ੀਏ ਨੇ ਤੁਹਾਡੇ ਨਾਲ **ਨਿਮਰਤਾ ਅਤੇ ਆਦਰ** ਨਾਲ ਵਿਹਾਰ ਕੀਤਾ?
    a. ਹਾਂ, ਨਿਸ਼ਚਤ ਤੌਰ ‘ਤੇ
    b. ਹਾਂ, ਕੁਝ ਹੱਦ ਤੱਕ
    c. ਨਹੀਂ

10. 0 ਤੋਂ ਲੈ ਕੇ 10 ਤੱਕ ਕਿਸੇ ਵੀ ਨੰਬਰ ਦੀ ਵਰਤੋਂ ਕਰਦੇ ਹੋਏ, ਜਿੱਥੇ 0 ਸੰਭਵ ਸਭ ਤੋਂ ਖਰਾਬ ਦੁਭਾਸ਼ੀਆ ਹੈ ਅਤੇ 10 ਸੰਭਵ ਸਭ ਤੋਂ ਵਧੀਆ ਦੁਭਾਸ਼ੀਆ ਹੈ, **ਤੁਸੀਂ ਇਸ ਦੁਭਾਸ਼ੀਏ ਨੂੰ ਰੇਟ ਕਰਨ ਦੇ ਲਈ ਕਿਹੜੇ ਨੰਬਰ ਦੀ ਵਰਤੋਂ ਕਰੋਗੇ**?
    a. 0 ਸੰਭਵ ਸਭ ਤੋਂ ਖਰਾਬ ਦੁਭਾਸ਼ੀਆ
    b. 1
    c. 2
    d. 3
    e. 4
    f. 5
    g. 6

h. 7
i. 8
j. 9
k. 10 ਸੰਭਵ ਵਧੀਆ ਦੁਭਾਸ਼ੀਆ

11. ਕੀ ਪ੍ਰਦਾਤੇ ਨੇ ਤੁਹਾਨੂੰ ਇੱਕ ਫਾਲੋ-ਅੱਪ ਅਪੌਇੰਟਮੈਂਟ ਦੇ ਲਈ ਵਾਪਸ ਆਉਣ ਦੇ ਲਈ ਕਿਹਾ?
    a. ਹਾਂ
    b. ਨਹੀਂ

12. ਕੀ ਤੁਹਾਡੀ ਫਾਲੋ-ਅੱਪ ਅਪੌਇੰਟਮੈਂਟ ਦੇ ਲਈ ਇੱਕ ਦੁਭਾਸ਼ੀਆ ਨਿਰਧਾਰਤ ਕੀਤਾ ਗਿਆ ਸੀ?
    a. ਹਾਂ
    b. ਨਹੀਂ
    c. ਮੈਨੂੰ ਨਹੀਂ ਪਤਾ

13. ਕੀ ਪ੍ਰਦਾਤੇ ਨੇ ਤੁਹਾਨੂੰ ਦਵਾਈ ਦੇ ਲਈ ਪ੍ਰੀਸਕ੍ਰਿਪਸ਼ਨ ਭਰਨ ਦੇ ਲਈ ਕਿਹਾ?
    a. ਹਾਂ
    b. ਨਹੀਂ

14. ਕੀ ਦੁਭਾਸ਼ੀਏ ਨੇ ਇਹ **ਸਮਝਣ ਵਿੱਚ ਤੁਹਾਡੀ ਮਦਦ ਕੀਤੀ** ਕਿ ਦਵਾਈ ਨੂੰ ਕਿਵੇਂ ਲੈਣਾ ਹੈ?
    a. ਹਾਂ, ਨਿਸ਼ਚਤ ਤੌਰ 'ਤੇ
    b. ਹਾਂ, ਕੁਝ ਹੱਦ ਤੱਕ
    c. ਨਹੀਂ

15. ਕੀ ਤੁਹਾਨੂੰ ਪਤਾ ਸੀ ਕਿ ਇੱਕ ਦੁਭਾਸ਼ੀਆ ਪ੍ਰਿਸਕ੍ਰਿਪਸ਼ਨ ਦਵਾਈ ਲੈਣ ਦੇ ਲਈ ਹਿਦਾਇਤਾਂ ਦਾ ਅਨੁਵਾਦ ਕਰ ਸਕਦਾ ਹੈ?
    a. ਹਾਂ
    b. ਨਹੀਂ

16. ਪਿਛਲੇ 12 ਮਹੀਨਿਆਂ ਦੇ ਦੌਰਾਨ, ਕੀ ਤੁਹਾਡੀ ਇੱਕ ਦੁਭਾਸ਼ੀਏ ਤੋਂ **ਬਿਨਾਂ** ਅਪੌਇੰਟਮੈਂਟ ਹੋਈ ਸੀ?
    a. ਹਾਂ
    b. ਨਹੀਂ

17. ਜਦੋਂ ਤੁਹਾਡੇ ਕੋਲ ਦੁਭਾਸ਼ੀਆ ਨਹੀ ਸੀ ਤਾਂ ਤੁਹਾਨੂੰ ਗੱਲਾਂ ਸਮਝਣ ਵਿੱਚ ਕਿਸ ਨੇ ਮਦਦ ਕੀਤੀ? ਲਾਗੂ ਹੋਣ ਵਾਲੇ ਸਾਰੇ ਵਿਕਲਪਾਂ ਨੂੰ ਚੁਣੋ।
    a. ਦੋਸਤ
    b. ਪਰਿਵਾਰਕ ਸਦੱਸ
    c. ਕਲੀਨਿਕ ਸਟਾਫ

18. ਦੁਭਾਸ਼ੀਏ ਦੇ ਨਾਲ ਤੁਹਾਡੀ ਹਾਲ ਹੀ ਦੀ ਅਪੌਇੰਟਮੈਂਟ ਦੁਭਾਸ਼ੀਏ ਤੋਂ ਬਿਨਾਂ ਦੇ ਤੁਹਾਡੇ ਅਤੀਤ ਦੇ **ਅਨੁਭਵਾਂ** ਨਾਲੋਂ ਕਿਵੇਂ ਵੱਖਰੀ ਸੀ?
    a. ਬਿਹਤਰ
    b. ਲਗਭਗ ਉਂਝ ਹੀ
    c. ਬਦਤਰ

19. ਤੁਸੀਂ ਕਲੀਨਿਕ ਵਿਖੇ **ਆਪਣੇ ਸਭ ਤੋਂ ਹਾਲ ਹੀ ਦੇ ਅਨੁਭਵ ਨੂੰ ਰੇਟ** ਕਰਨ ਦੇ ਲਈ ਕਿਹੜੇ ਨੰਬਰ ਦੀ ਵਰਤੋਂ ਕਰੋਗੇ? 0 ਤੋ ਲੈ ਕੇ 10 ਤੱਕ ਕਿਸੇ ਵੀ ਨੰਬਰ ਦੀ ਵਰਤੋਂ ਕਰੋ, ਜਿੱਥੇ 0 ਸੰਭਵ ਸਭ ਤੋ ਖਰਾਬ ਕਲੀਨਿਕ ਅਨੁਭਵ ਹੈ ਅਤੇ 10 ਸੰਭਵ ਸਭ ਤੋ ਵਧੀਆ ਕਲੀਨਿਕ ਅਨੁਭਵ ਹੈ।

a. 0 ਸੰਭਵ ਸਭ ਤੋਂ ਬਦਤਰ ਅਨੁਭਵ
b. 1
c. 2
d. 3
e. 4
f. 5
g. 6
h. 7
i. 8
j. 9
k. 10 ਸੰਭਵ ਸਭ ਤੋਂ ਵਧੀਆ ਅਨੁਭਵ

20. ਤੁਹਾਡਾ ਲਿੰਗ ਕੀ ਹੈ?
    a. ਆਦਮੀ
    b. ਔਰਤ
    c. ਕੋਈ ਹੋਰ

21. ਤੁਸੀਂ ਕਿੰਨੀ ਵਧੀਆ ਅੰਗ੍ਰੇਜ਼ੀ ਬੋਲਦੇ ਹੋ?
    a. ਬਹੁਤ ਵਧੀਆ
    b. ਵਧੀਆ
    c. ਵਧੀਆ ਨਹੀਂ
    d. ਬਿਲਕੁਲ ਵੀ ਨਹੀਂ

22. ਤੁਸੀਂ ਆਪਣੀ ਸਮੁੱਚੀ ਸਿਹਤ ਨੂੰ ਕਿਵੇਂ ਰੇਟ ਕਰੋਗੇ?
    a. ਸਰਬੋਤਮ
    b. ਬਹੁਤ ਵਧੀਆ
    c. ਵਧੀਆ
    d. ਠੀਕ-ਠਾਕ
    e. ਖਰਾਬ

ਇਸ ਅਧਿਐਨ ਵਿੱਚ ਭਾਗੀਦਾਰੀ ਕਰਨ ਦਾ ਸਮਾਂ ਕੱਢਣ ਦੇ ਲਈ ਤੁਹਾਡਾ ਧੰਨਵਾਦ। ਅਸੀ ਤੁਹਾਨੂੰ ਅਗਲੇ 48 ਘੰਟਿਆਂ ਦੇ ਅੰਦਰ **ਤੁਹਾਡੇ ਈਮੇਲ ਪਤੇ 'ਤੇ ਜਾਂ ਟੈਕਸਟ ਦੇ ਰਾਹੀਂ ਤੁਹਾਡੇ ਫੋਨ ਨੰਬਰ 'ਤੇ** ਇੱਕ $10 ਦਾ ਇਲੈੱਕਟਰ੍ਰੋਨਿਕ ਗਿਫਟ ਕਾਰਡ ਭੇਜਾਂਗੇ। ਕਿਰਪਾ ਕਰਕੇ ਦੱਸੋ ਕਿ ਤੁਹਾਡੇ ਲਈ ਸਭ ਤੋਂ ਢੁਕਵਾਂ ਤਰੀਕਾ ਕਿਹੜਾ ਹੈ?

a) ਈਮੇਲ
b) ਟੈਕਸਟ

$10 ਦਾ ਇਲੈੱਕਟ੍ਰੌਨਿਕ ਗਿਫਟ ਕਾਰਡ ਭੇਜਣ ਦੇ ਲਈ ਤੁਹਾਡਾ ਈਮੇਲ ਪਤਾ ਕੀ ਹੈ?

$10 ਦਾ ਇਲੈੱਕਟ੍ਰੌਨਿਕ ਗਿਫਟ ਕਾਰਡ ਭੇਜਣ ਦੇ ਲਈ ਤੁਹਾਡਾ ਫੋਨ ਨੰਬਰ ਕੀ ਹੈ?

ਕੀ ਤੁਸੀਂ ਇੱਕ ਇੰਟਰਵਿਊਰ ਦੇ ਨਾਲ ਆਪਣੇ ਹਾਲ ਹੀ ਦੇ ਸਿਹਤ ਦੇਖਭਾਲ ਅਨੁਭਵ ਬਾਰੇ ਵਿਸਤਾਰ ਵਿੱਚ ਚਰਚਾ ਕਰਨ ਲਈ ਦਿਲਚਸਪ ਹੋ? ਅਸੀਂ ਤੁਹਾਨੂੰ ਇੱਕ ਗੁਪਤ 30-ਮਿੰਟਾਂ ਦੇ ਇੰਟਰਵਿਊ ਵਿੱਚ ਤੁਹਾਡੀ ਭਾਗੀਦਾਰੀ ਦੇ ਲਈ ਇੱਕ ਵਾਧੂ **$25 ਦਾ ਇਲੈੱਕਟੌਨਿਕ ਗਿਫਟ ਕਾਰਡ** ਦਿਆਂਗੇ।

a) ਹਾਂ, ਮੈਂ ਦਿਲਚਸਪ ਹਾਂ
b) ਨਹੀਂ, ਮੈਂ ਦਿਲਚਸਪ ਨਹੀਂ ਹਾਂ

ਆਉਣ ਵਾਲੇ ਇੰਟਰਵਿਊ ਦੇ ਲਈ ਤਿਆਰੀ ਕਰਨ ਵਿੱਚ ਤੁਹਾਡੀ ਮਦਦ ਕਰਨ ਦੇ ਲਈ:

ਤੁਹਾਨੂੰ ਸੰਪਰਕ ਕਰਨ ਦੇ ਲਈ ਸਰਬੋਤਮ **ਫੋਨ ਨੰਬਰ** ਕਿਹੜਾ ਹੈ?

ਤੁਹਾਨੂੰ ਸੰਪਰਕ ਕਰਨ ਦੇ ਲਈ ਸਰਬੋਤਮ **ਈਮੇਲ ਪਤਾ** ਕਿਹੜਾ ਹੈ?

ਇੱਕ ਖੋਜਕਰਤਾ ਤੁਹਾਨੂੰ ਅਗਲੇ ਦੋ ਹਫਤਿਆਂ ਦੇ ਅੰਦਰ ਤੁਹਾਡੇ ਲਈ ਸਭ ਤੋ ਅਨੁਕੂਲ ਸਮੇਂ 'ਤੇ ਫੋਨ ਕਰੇਗਾ। ਕਿਰਪਾ ਕਰਕੇ ਆਪਣੀ ਸਮਾਂ-ਸਾਰਨੀ ਦੇ ਲਈ **ਢੁਕਵੇਂ ਸਾਰੇ ਸਮਿਆਂ** ਨੂੰ ਚੁਣੋ। ਸਮੇਂ ਪੈਸੀਫਿਕ ਸਟੈਂਡਰਡ ਟਾਈਮ (ਕੈਲੀਫੋਰਨੀਆ ਸਮੇਂ) ਵਿੱਚ ਦਿੱਤੇ ਹੋਏ ਹਨ।

a) ਹਫਤੇ ਦੇ ਦਿਨ (ਸੋਮਵਾਰ ਤੋਂ ਸ਼ੁੱਕਰਵਾਰ) 11AM-2PM
b) ਹਫਤੇ ਦੇ ਦਿਨ (ਸੋਮਵਾਰ ਤੋਂ ਸ਼ੁੱਕਰਵਾਰ) 3PM-6PM
c) ਸ਼ਨੀਵਾਰ 9AM-1PM
d) ਕੋਈ ਹੋਰ

ਜੇਕਰ ਤੁਸੀਂ "ਹੋਰ" ਨੂੰ ਚੁਣਦੇ ਹੋ, ਤਾਂ ਤੁਹਾਡੀ ਸਮਾਂ-ਸਾਰਣੀ ਦੇ ਲਈ **ਹਫਤੇ ਦੇ ਕਿਹੜੇ ਦਿਨ ਅਤੇ ਸਮੇਂ** ਤੁਹਾਡੇ ਲਈ ਸਰਬੋਤਮ ਕੰਮ ਕਰਨਗੇ?

ਇੱਕ ਇੰਟਰਵਿਊ ਵਿੱਚ ਭਾਗ ਲੈਣ ਵਿੱਚ ਤੁਹਾਡੀ ਦਿਲਚਸਪੀ ਲਈ ਤੁਹਾਡਾ ਧੰਨਵਾਦ। ਕਿਰਪਾ ਕਰਕੇ ਆਪਣੇ ਇੰਟਰਵਿਊ ਦੇ ਸਮੇਂ ਨੂੰ ਨਿਰਧਾਰਤ ਕਰਨ ਦੇ ਲਈ **ਇਸ ਕੈਲੰਡਰ** ਤੋ ਇੱਕ ਤਾਰੀਖ਼ ਅਤੇ ਸਮਾਂ ਚੁਣੋ। ਤੁਹਾਡੇ ਇੰਟਰਵਿਊ ਤੋ ਪਹਿਲਾਂ ਤੁਹਾਨੂੰ ਇੱਕ ਪੁਸ਼ਟੀਕਰਨ ਮੈਸੇਜ ਭੇਜਿਆ ਜਾਵੇਗਾ।

ਸਮਾਪਤੀ:

1) "ਇਹ ਸਰਵੇਖਣ ਸਮਾਪਤ ਹੋ ਗਿਆ ਹੈ ਕਿਉਂਕਿ ਤੁਸੀ ਭਾਗ ਲੈਣ ਦੇ ਲਈ ਸਹਿਮਤੀ ਨਹੀ ਦਿੱਤੀ। ਜੇਕਰ ਤੁਸੀ ਹਾਲੇ ਵੀ ਭਾਗੀਦਾਰੀ ਕਰਨਾ ਚਾਹੁੰਦੇ ਹੋ, ਤਾਂ ਕਿਰਪਾ ਕਰਕੇ ਸਰਵੇਖਣ ਨੂੰ ਦੁਬਾਰਾ ਸ਼ੁਰੂ ਕਰਨ ਦੇ ਲਈ ਆਪਣੇ ਬਰ੍ਾਊਜ਼ਰ ਨੂੰ ਰੀਫਰੈ੍ਸ਼ ਕਰੋ। ਅਧਿਐਨ ਜਾਣਕਾਰੀ ਸ਼ੀਟ ਦਾ ਲਿੰਕ **ਇੱਥੇ** ਸ਼ਾਮਲ ਹੈ।"

2) "ਇਸ ਸਰਵੇਖਣ ਨੂੰ ਪੂਰਾ ਕਰਨ ਦੇ ਲਈ ਤੁਹਾਡਾ ਧੰਨਵਾਦ ਅਤੇ ਇੱਕ ਇੰਟਰਵਿਊ ਵਿੱਚ ਭਾਗੀਦਾਰੀ ਕਰਨ ਵਿੱਚ ਤੁਹਾਡੀ ਦਿਲਚਸਪੀ ਦੇ ਲਈ ਤੁਹਾਡਾ ਧੰਨਵਾਦ। ਤੁਹਾਡੀ ਫੀਡਬੈਕ ਤੁਹਾਡੇ ਵਰਗੇ ਮਰੀਜ਼ਾਂ ਦੇ ਲਈ ਸੇਵਾਵਾਂ ਵਿੱਚ ਸੁਧਾਰ ਕਰਨ ਦੇ ਲਈ ਮਦਦ ਕਰੇਗੀ। ਤੁਹਾਡੇ ਇੰਟਰਵਿਊ ਦੀ ਤਾਰੀਖ਼ ਅਤੇ ਸਮੇਂ ਬਾਰੇ ਆਉਣ ਵਾਲੇ ਦਿਨਾਂ ਵਿੱਚ ਤੁਹਾਡੇ ਨਾਲ ਪੁਸ਼ਟੀ ਕੀਤੀ ਜਾਵੇਗੀ। ਅਧਿਐਨ ਜਾਣਕਾਰੀ ਸ਼ੀਟ ਦਾ ਲਿੰਕ **ਇੱਥੇ** ਸ਼ਾਮਲ ਹੈ।"

3) "ਇਸ ਸਰਵੇਖਣ ਨੂੰ ਪੂਰਾ ਕਰਨ ਦੇ ਲਈ ਧੰਨਵਾਦ। ਤੁਹਾਡੇ ਜਵਾਬ ਤੁਹਾਡੇ ਵਰਗੇ ਮਰੀਜ਼ਾਂ ਦੇ ਲਈ ਸੇਵਾਵਾਂ ਵਿੱਚ ਸੁਧਾਰ ਕਰਨ ਦੇ ਲਈ ਮਦਦ ਕਰਨਗੇ। ਅਧਿਐਨ ਜਾਣਕਾਰੀ ਸ਼ੀਟ ਦਾ ਲਿੰਕ **ਇੱਥੇ** ਸ਼ਾਮਲ ਹ

Russian

Анкета пациента

На каком **языке** Вы разговариваете дома?

Арабский
Армянский
Бирманский
Камбоджийский (центральный кхмерский)
Кантонский китайский
Мандаринский китайский
Дари
Фарси
Гаитянский креольский
Хинди
Хмонг
Японский
Каренский
Корейский
Лаосский
Оромо
Пашто
Португальский
Пенджаби
Русский
Сомалийский
Испанский
Суахили
Тагальский
Тайский
Тигринья
Украинский
Вьетнамский
Другое

1. Ваша дата рождения:

2. Дата Вашего самого последнего обращения в клинику:

3. Врач (или другой медик) **выслушал Вас внимательно**?
   a. Да, безусловно
   b. Да, в некотором роде
   c. Нет

4. Проявлял ли врач (или другой медик) **уважение** к тому, что Вы говорили?

   a. Да, безусловно
   b. Да, в некотором роде
   c. Нет

5. **Поощрял** ли врач (или другой медик) **Ваши вопросы**?
   a. Да, безусловно
   b. Да, в некотором роде
   c. Нет

6. **Достаточно ли времени провел** с Вам врач (или другой медик)?
   a. Да, безусловно
   b. Да, в некотором роде
   c. Нет

7. Устный переводчик **помогал Вам объяснять** врачу (или другому медперсоналу), что Вы чувствуете и на что жалуетесь?
   a. Да, безусловно
   b. Да, в некотором роде
   c. Нет

8. Устный переводчик **помогал Вам разобраться в назначениях** врача (или другого медика)?
   a. Да, безусловно
   b. Да, в некотором роде
   c. Нет

9. Переводчик был с Вами **вежлив и оказывал Вам уважение**?
   a. Да, безусловно
   b. Да, в некотором роде
   c. Нет

10. **Как бы Вы оценили этого переводчика**? Ответ дайте по шкале от 0 до 10, где 0 — «хуже некуда», а 10 — «лучше не бывает».
   a. 0 Хуже некуда
   b. 1
   c. 2
   d. 3
   e. 4
   f. 5
   g. 6
   h. 7
   i. 8
   j. 9
   k. 10 Лучше не бывает

11. Врач (или другой медик) попросил Вас прийти еще раз?

a. Да
b. Нет

12. Был ли на следующий прием вызван устный переводчик?
    a. Да
    b. Нет
    c. Не знаю

13. Врач (или другой медик) сказал, что Вам нужно получить лекарство по рецепту?
    a. Да
    b. Нет

14. Устный переводчик **объяснил**, как принимать это лекарство?
    a. Да, безусловно
    b. Да, в некотором роде
    c. Нет

15. Знаете ли Вы, что устный переводчик может перевести Вам инструкции по приему лекарства?
    a. Да
    b. Нет

16. За последние 12 месяцев Вам приходилось бывать на приеме у врача (или другого медика) **без** переводчика?
    a. Да
    b. Нет

17. Кто Вам переводил, когда переводчика не было? Отметьте все подходящие ответы.
    a. Друг
    b. Член семьи
    c. Сотрудник клиники

18. Сравните впечатления от своего последнего приема у врача (или медика), на котором был устный переводчик, с **прошлыми** приемами, на которых устного переводчика не было?
    a. Лучше
    b. Примерно так же
    c. Хуже

19. Как бы **Вы оценили свои впечатления от последнего приема** у врача (или медика) в своей клинике? Ответ дайте по шкале от 0 до 10, где 0 — «хуже некуда», а 10 — «лучше не бывает».
    a. 0 Хуже некуда
    b. 1

c. 2
d. 3
e. 4
f. 5
g. 6
h. 7
i. 8
j. 9
k. 10 Лучше не бывает

20. Ваш пол?
    a. Мужчина
    b. Женщина
    c. Другое

21. Насколько хорошо Вы говорите по-английски?
    a. Очень хорошо
    b. Хорошо
    c. Плохо
    d. Совсем не говорите

22. Как Вы оценили бы общее состояние своего здоровья?
    a. Отличное
    b. Очень хорошее
    c. Хорошее
    d. Неплохое
    e. Плохое

Спасибо за участие в этом исследовании. В течение 48 часов мы пришлем Вам электронную подарочную карту на $10 **на Ваш электронный адрес или мобильный телефон**. Укажите, что Вы предпочитаете.
a) Электронная почта
b) Текстовое сообщение

На какой электронный адрес Вам прислать электронную подарочную карту на $10?

На какой номер мобильного телефона Вам прислать электронную подарочную карту на $10?

Хотели бы Вы обсудить с нашим сотрудником свои недавние впечатления от полученной медицинской помощи? Это будет 30-минутное собеседование. За него Вы получите еще одну **электронную подарочную карту, на этот раз на $25**.
a) Да, хотелось бы
b) Нет, не хотелось бы

Для того чтобы назначить собеседование, укажите следующее:

По какому **телефону** с Вами связаться?

По какому **электронному адресу** с Вами связаться?

Исследователь позвонит Вам в течение следующих двух недель в удобное для Вас время. Укажите все **удобные для Вас дни и удобное для Вас** время. Время тихоокеанское (Pacific Standard Time, калифорнийское время).

a) Будние дни (с понедельника по пятницу включительно) с 11:00 до 14:00.
b) Будние дни (с понедельника по пятницу включительно) с 15:00 до 18:00.
c) Суббота с 9:00 до 13:00.
d) Другое

Если выбран ответ «Другое», **в какие дни недели и в какое время** Вам было бы удобнее всего пройти собеседование?

Спасибо за интерес к собеседованию. Выберите дату и время собеседования по **этому календарю**. Вам заранее пришлют сообщение о дате и времени собеседования.

Завершение:
1) «Анкетирование прекращено, поскольку Вы отказались отвечать на вопросы анкеты. Если Вы передумали, обновите в своем браузере эту страницу и начните анкетирование заново. Информационный листок об исследовании есть **здесь**».

2) «Спасибо за ответы на вопросы анкеты и за интерес к собеседованию. Ваши ответы помогут улучшить обслуживание пациентов. В ближайшие дни Вы получите сообщение о дате и времени собеседования. Информационный листок об исследовании есть **здесь**».

3) «Спасибо за ответы на вопросы анкеты. Они помогут улучшить обслуживание пациентов. Информационный листок об исследовании есть **здесь**».

Somali

Sahanka Bukaanka

Luuqadee ayaad guriga ugu hadashaa?

Carabi
Armenian
Burmese
Cambodian (Central Khmer)

Chinese (Cantonese)
Chinese (Mandarin)
Dari
Farsi
Haitian Creole
Hindi
Hmong
Japanese
Karen
Korean
Laotian
Oromo
Pashto
Portuguese
Punjabi
Russian
Somali
Spanish
Swahili
Tagalog
Thai
Tigrinya
Ukrainian
Vietnamese
Kuwo kale

1. Taariikhdaada dhalasho:

2. Taariikhda kugu dhoweyd ee aad balan la yeelato bukaan-eegtada:

3. Miyuu adeeg bixiyuhu **si aayar kuu dhegeystay**?
    a. Haa, aad iyo aad
    b. Haa, xoogaa
    c. Maya

4. Miyuu addeeg bixiyuhu **kuu muujiyey xushmo** wixii aad dhahday?
    a. Haa, aad iyo aad
    b. Haa, xoogaa
    c. Maya

5. Miyuu addeeg bixiyuhu **kugu dhiirogeliyey inaad weydiiso su'aalo?**
    a. Haa, aad iyo aad
    b. Haa, xoogaa
    c. Maya

6. Miyuu addeeg bixiyuhu **ku siiyey waqti kugu filan oo aad su'aalo ku weydiiso?**
    a. Haa, aad iyo aad
    b. Haa, xoogaa
    c. Maya

7. Miyuu turjumaanku **kaa caawiyey inuu** addeeg bixiyuhu **u sharaxo** sidaad dareemaysay
    a. Haa, aad iyo aad
    b. Haa, xoogaa
    c. Maya

8. Miyuu turjumaanku **kaa caawiyey inaad fahanto** tilmaamaha addeeg bixiyuhu ka imaanayey?
    a. Haa, aad iyo aad
    b. Haa, xoogaa
    c. Maya

9. Miyuu turjumaanka kuula dhaqmay si **ixtiraam iyo xushmo** leh?
    a. Haa, aad iyo aad
    b. Haa, xoogaa
    c. Maya

10. Adigoo isticmaalaya lambarada laga soo billaabo 0 ilaa 10, halka 0 ay tahay turjumaanka ugu xun ee ugu macquulsan iyo 10 oo ah turjumaanka ugu fiican ee ugu macquulsan, **lambarkee ayaad isticmaali laheyd si aad dhibco u siiso turjumaankan**?
    a. 0 Turjumaanka ugu xun sida macquulka ah
    b. 1
    c. 2
    d. 3
    e. 4
    f. 5
    g. 6
    h. 7
    i. 8
    j. 9
    k. 10 Turjumaanka ugu fiican sida macquulka ah

11. Miyuu addeeg bixiyuhu ku warsaday inaad dib ugu soo noqoto si balan raadraac loo sameeyo?
    a. Haa
    b. Maya

12. Miyuu turjumaanku balantaada raadraaca kuu dhigay?
    a. Haa

    b. Maya
    c. Ma garanayo

13. Miyuu addeeg bixiyuhu kuu sheegay inaad soo buuxiso warqada daawada laguu qoray?
    a. Haa
    b. Maya

14. Miyuu turjumaanku **kaa caawiyey inaad fahanto** sida daawada loo qaato?
    a. Haa, aad iyo aad
    b. Haa, xoogaa
    c. Maya

15. Ma ogtahay in turjumaanku kuu turjumi karo tilmaamaha qaadashada waraaqda daawada laguu qoray?
    a. Haa
    b. Maya

16. 12 biloodkii ugu dambeeyey, ma dhigatay balan oo **aan** turjumaan kula jirin
    a. Haa
    b. Maya

17. Yaa kaa caawiyey inuu kuu turjumo markaad turjumaan la'aan aheyd? Astee dhammaan waxa ku quseeeya.
    a. Saaxiib
    b. Xubin qoys
    c. Shaqaalaha xarunta

18. Sidee baad isugula barbardhigi kartaa balantii dhowaan turjumaan kula jirey iyo balantii **hore** aan turjumaan kula jirin?
    a. Ka fiican
    b. Isku mid bay ahaayeen
    c. Wau xumeyd

19. Lambarkee ayaad siin laheyd si aad dhibco **u siiso waayo aragnimadaada dhowaan** kala kulanta xarunta bukaan-eegtada? Isticmaal lambar kasta laga soo billaabo 0 ilaa 10, halka 0 ay tahay xarunta bukaan-eegtada ugu xun ee waayo aragnimadaada ugu macquulsan iyo 10 oo ah xarunta bukaan-eegtada ugu fiican ee waayo aragnimada ugu macquulsan.
    a. 0 Ugu xun waayo aragnimo macquulsan
    b. 1
    c. 2
    d. 3
    e. 4
    f. 5
    g. 6

    h. 7
    i. 8
    j. 9
    k. 10 Waayo aragnimada ugu macquulsan

20. Nooca jinsigaagu muxuu yahay?
    a. Lab
    b. Dheddig
    c. Kuwo kale

21. Sidee ugu fiican ayaad Ingiriiska ugu hadli kartaa?
    a. Si aad u fiican
    b. Si fiican
    c. Si aan fiicneen
    d. Haba yaraatee

22. Sidee baad dhibco ugu siin laheyd caafimaadka guud?
    a. Heer sare
    b. Si aad u fiican
    c. Fiican
    d. Dhexdhexaad
    e. Liita

Waad ku mahadsan tahay waqtigaad qaadatay ka qeybgalka daraasadan. Waxaanu kuu soo diri doonaa $10 oo kaar hadiyad elektoroonik ah cinwaanka emaylkaaga ama teleefankaaga qoraalaha muddo 48 saacadood gudaheeda. Fadlan tilmaan qaabkee ayaa kuu shaqeysay kuugu wanaagsaneyd.

a) Emayl
b) Qoraal

Waa maxay cinwaanka iimaylka aan kugu soo dirno $10 Doolar ee kaar hadiyad qaab elektoroonik ah?

Waa maxay teleefan lambarkaaga aan kugu soo dirno $10 Doolar ee kaar hadiyad elektoroonik ah?

Ma daneynaysaa inaad ka hadasho waayo aragnimadaada daryeelka caafimaad ee dhowaan heshay iyadoo faahfaahsan ula yeelato wareysi qaadaha? Waxaanu ku siin doonaa $25 kaar hadiyad elektoroonik ah oo loogu talogalay ka qeyb qaadashada wareysiga sirta ee 30 daqiiqo gaaraya.

a) Haa, waan daneynayaa
b) Maya, ma daneynayo

Si lagaaga caawiyo qorshaha wareysigaaga soo socda:

Waa maxay **lambar teleefanka** ugu wanaagsan ee laga soo wici karo?

Waa maxay **cinwaanka iimaylka** ugu wanaagsan ee laguu soo diri karo?

Cilmi-baare ayaa ku soo wici doona labada isbuuc ee xiga inta lagu jiro waqti adiga kuu munaasab ah. Fadlan dooro **dhammaan waqtiga daaqadaha munaasabka** u ah balantaada Waqtiyada waxaa lagu soo taxayaa Waqtiga Pacifiga ee Caadiga ah (California).

a) Maalmaha shaqada (Isniin ilaa Jimce) 11AM-2PM
b) Maalmaha shaqada (Isniin ilaa Jimce) 3PM-6PM
c) Sabtida 9AM-1PM
d) Kuwo kale

Haddaad doorato "wax kale", **maalmehee baa isbuuca iyo waqtiga** oo si wanaagsan kuu munaasab ah in balan laguu dhigo?

Waad ku mahadsan tahay daneyntaada ah inaad kasoo qeybgasho. Fadlan dooro taariikhda iyo waqtiga laga soo billaabo **kalendarkan** si balan laguugu qabto wareysigaaga. Dhambaal xaqiijin ah ayaa laguu soo dirayaa kahor wareysiga.

Dhammaadka:
1) "Wareysiga wuu dhammaaday waayo ma aadan bixin ruqsad inaad ka qeybgasho. Haddaad weli rabto inaad ka qeybqaadato, fadlan dib u cusbooneysii boggaaga daalacashada si aad u soo bilowdo sahanka. Isku xidhe ku geeye xaashida macluumaadka daraasada ayaa ku jirta **halkan**."

2) Waad ku mahadsan tahay soo buuxinta sahanka iyo daneyntaada ah inaad kasoo qeybgasho wareysiga. Falcelintaada waxay ka caawin doontaa in wax laga bedelo addeegyada bukaanada loo fidiyo sida adigoo kale. Taariikhdaa iyo waqtigaaga wareysiga saxda ah waxaa laguu soo xaqiijinayaa maalmaha soo socda. Isku xidhe ku geeye xaashida macluumaadka daraasada ayaa ku jirta **halkan**."

3) Waad ku mahadsan tahay soo buuxinta sahanka. Jawaab celintaada waxa ka caawin doontaa in wax laga bedelo addeegyada bukaanada loo fidiyo sida adigoo kale. Isku xidhe. “

Spanish

Encuesta al paciente

¿Qué **idioma** habla en su casa?

Árabe
Armenio
Birmano
Camboyano (jemer central)
Chino (cantonés)
Chino (mandarín)
Darí
Persa
Criollo haitiano
Hindi
Hmong
Japonés
Karen
Coreano
Laosiano
Oromo
Pastún
Portugués
Punyabí
Ruso
Somalí
Español
Swahili
Tagalo
Tailandés
Tigriña
Ucraniano
Vietnamita
Otro

1. Fecha de nacimiento:

2. Fecha de su consulta clínica más reciente:

3. ¿El proveedor **escuchó con atención** lo que usted dijo?
    a. Sí, definitivamente
    b. Sí, en cierto modo
    c. No

4. ¿El proveedor **mostró respeto** por lo que usted tenía que decir?
    a. Sí, definitivamente
    b. Sí, en cierto modo
    c. No

5. ¿El proveedor **lo animó a que hiciera preguntas**?
    a. Sí, definitivamente
    b. Sí, en cierto modo
    c. No

6. ¿El proveedor **pasó tiempo suficiente** con usted?
    a. Sí, definitivamente
    b. Sí, en cierto modo
    c. No

7. ¿El intérprete **lo ayudó a explicarle** al proveedor cómo se estaba sintiendo?
    a. Sí, definitivamente
    b. Sí, en cierto modo
    c. No

8. ¿El intérprete **lo ayudó a entender las instrucciones** del proveedor?
    a. Sí, definitivamente
    b. Sí, en cierto modo
    c. No

9. ¿El intérprete lo trato con **cortés y respetuoso**?
    a. Sí, definitivamente
    b. Sí, en cierto modo
    c. No

10. Usando una escala del 0 al 10, donde 0 es el peor intérprete posible y 10 es el mejor intérprete posible, **¿qué número usaría para calificar a este intérprete?**
    a. 0 El peor intérprete posible
    b. 1
    c. 2
    d. 3
    e. 4
    f. 5
    g. 6
    h. 7
    i. 8
    j. 9
    k. 10 El mejor intérprete posible

11. ¿El proveedor le pidió que regrese para una consulta de seguimiento?
    a. Sí
    b. No

12. ¿Se programó un intérprete para la consulta de seguimiento?
    a. Sí
    b. No
    c. No sé

13. ¿El proveedor le dijo que complete una receta médica?
    a. Sí
    b. No

14. ¿El intérprete **lo ayudó a entender** cómo se toman los medicamentos recetados?
    a. Sí, definitivamente
    b. Sí, en cierto modo
    c. No

15. ¿Sabía que un intérprete puede traducir las instrucciones sobre cómo tomar los medicamentos recetados?
    a. Sí
    b. No

16. Durante los últimos 12 meses, ¿tuvo una consulta **sin** un intérprete?
    a. Sí
    b. No

17. ¿Quién lo ayudó con la interpretación cuando no tuvo un intérprete? Marque todas las opciones que correspondan.
    a. Un amigo
    b. Un miembro de la familia
    c. El personal de la clínica

18. ¿Cómo compara su última consulta con la presencia del intérprete con respecto a las consultas **anteriores** sin un intérprete?
    a. Mejor
    b. Casi igual
    c. Peor

19. ¿Qué número usaría para **calificar su experiencia más reciente** en la clínica? Use un número del 0 al 10, donde 0 es la peor experiencia posible y 10 es la mejor experiencia posible.
    a. 0 La peor experiencia posible
    b. 1
    c. 2

d. 3
e. 4
f. 5
g. 6
h. 7
i. 8
j. 9
k. 10 La mejor experiencia posible

20. ¿Cuál es su género?
    a. Masculino
    b. Femenino
    c. Otro

21. ¿Cuál es su nivel de inglés?
    a. Muy bueno
    b. Bueno
    c. No muy bueno
    d. Malo

22. ¿Cómo calificaría su estado de salud general?
    a. Excelente
    b. Muy bueno
    c. Bueno
    d. Razonable
    e. Malo

Gracias por tomarse el tiempo para participar en este estudio. Le enviaremos una tarjeta de regalo electrónica de $10 a **su dirección de correo electrónico o a su teléfono móvil por mensaje de texto** dentro de las próximas 48 horas. Indique el método que prefiere.

a) Correo electrónico
b) Mensaje de texto

¿A qué dirección de correo electrónico podemos enviarle la tarjeta de regalo electrónica de $10?

¿A qué número de teléfono móvil podemos enviarle la tarjeta de regalo electrónica de $10?

¿Le interesa conversar en mayor detalle sobre su experiencia de salud más reciente con un entrevistador? Le daremos una **tarjeta de regalo electrónica de $25** adicional por su participación en una entrevista confidencial de 30 minutos.

a) Sí, me interesa.
b) No, no me interesa.

Para ayudar a planear su próxima entrevista:

¿A qué **número de teléfono** prefiere que nos comuniquemos?

¿A qué **dirección de correo electrónico** prefiere que nos comuniquemos?

Un investigador le llamará dentro de las próximas dos semanas en el horario que le resulte más conveniente. Elija **todos los horarios posibles que le convengan** según sus horarios. Los horarios corresponden a la hora estándar del Pacífico (hora de California).

a) Días dentro de la semana (de lunes a viernes) 11 a. m. a 2 p. m.
b) Días dentro de la semana (de lunes a viernes) 3 p. m. a 6 p. m.
c) Sábado 9 a. m. a 1 p. m.
d) Otro

Si elige «Otro», ¿**qué días de la semana y horarios** le resultan más convenientes según sus horarios?

Gracias por su interés en participar en una entrevista. Elija una fecha y hora de **este calendario** para programar su entrevista. Recibirá un mensaje de confirmación antes de su entrevista.

Finales:
1) «La encuesta finalizó porque no dio su consentimiento para participar. Si todavía desea hacerlo, recarga la página o el navegador de Internet para reiniciar la encuesta. Haga clic **aquí** para ir a la hoja de información del estudio».

2) «Gracias por completar la encuesta y por su interés en participar en una entrevista. Su opinión ayudará a mejorar los servicios para pacientes como usted. Le confirmarán la fecha y hora exactas en los próximos días. Haga clic **aquí** para ir a la hoja de información del estudio».

3) «Gracias por completar la encuesta. Sus respuestas ayudarán a mejorar los servicios para pacientes como usted. Haga clic **aquí** para ir a la hoja de información del estudio».

Swahili

Utafiti wa Mgonjwa

Huwa unazungumza **lugha** gani ukiwa nyumbani?

Kiarabu

Kiarmenia
Kiburma
Kikambodia (Kikhmeri cha Kati)
Kichina (Cantonese)
Kichina (Mandarin)
Kidari
Kifarsi
Krioli ya Haiti
Kihindi
Kihmong
Kijapani
Kikareni
Kikorea
Kilaoti
Kioromo
Kipashto
Kireno
Kipunjabi
Kirusi
Kisomali
Kihispania
Kiswahili
Kitagalogi
Kitai
Kitigrinya
Kiukrania
Kivietinamu
Nyingine

1. Tarehe yako ya kuzaliwa:

2. Tarehe ya miadi yako ya kliniki ya hivi karibuni zaidi:

3. Je, mtoaji **alikusikiliza kwa umakini**?
    a. Ndiyo, bila shaka
    b. Ndiyo, kiasi
    c. Hapana

4. Je, mtoaji **alionyesha heshima** kwa kile ulichosema?
    a. Ndiyo, bila shaka
    b. Ndiyo, kiasi
    c. Hapana

5. Je, mtoaji **alikuhimiza kuuliza maswali**?
    a. Ndiyo, bila shaka
    b. Ndiyo, kiasi
    c. Hapana

6. Je, mtoaji **alitumia muda wa kutosha** na wewe?
    a. Ndiyo, bila shaka
    b. Ndiyo, kiasi
    c. Hapana

7. Je, mkalimani **alikusaidia kumuelezea** mtoaji jinsi ambavyo ulikuwa unajisikia?
    a. Ndiyo, bila shaka
    b. Ndiyo, kiasi
    c. Hapana

8. Je, mkalimani **alikusaidia kuelewa maelekezo** kutoka kwa mtoaji?
    a. Ndiyo, bila shaka
    b. Ndiyo, kiasi
    c. Hapana

9. Je, mkalimani alikuhudumia kwa **heshima na adabu**?
    a. Ndiyo, bila shaka
    b. Ndiyo, kiasi
    c. Hapana

10. Kwa kutumia nambari yoyote kutoka 0 hadi 10, ambapo 0 ni mkalimani mbaya zaidi zaidi na 10 ni mkalimani bora zaidi, **utatumia nambari gani kukadiria mkalimani huyu**?
    a. 0 Mkalimani mbaya zaidi zaidi
    b. 1
    c. 2
    d. 3
    e. 4
    f. 5
    g. 6
    h. 7
    i. 8
    j. 9
    k. 10 Mkalimani bora zaidi

11. Je, mtoaji alikuuliza urudi kwenye miadi ya kufuatilia?
    a. Ndiyo
    b. Hapana

12. Je, mkalimani aliratibiwa kwa ajili ya miadi yako ya kufuatilia?
    a. Ndiyo
    b. Hapana
    c. Sijui

13. Je, mtoaji alikuambia ujaze pendekezo la dawa?
    a. Ndiyo
    b. Hapana

14. Je, mkalimani **alikusaidia kuelewa** jinsi ya kutumia dawa?
    a. Ndiyo, bila shaka
    b. Ndiyo, kiasi
    c. Hapana

15. Je, ulijua kuwa mkalimani anaweza kutafsiri maelekezo ya kutumia dawa iliyopendekezwa?
    a. Ndiyo
    b. Hapana

16. Katika miezi 12 iliyopita, ulikuwa na miadi **bila** mkalimani?
    a. Ndiyo
    b. Hapana

17. Ni nani alisaidia kukalimani wakati ambapo hukuwa na mkalimani? Weka alama kwa zote zinazotumika.
    a. Rafiki
    b. Mwanafamilia
    c. Mfanyakazi wa kliniki

18. Miadi yako ya hivi karibuni zaidi na mkalimani inalinganaje na miadi yako **iliyopita** bila mkalimani?
    a. Bora
    b. Inakaribia kuwa sawa
    c. Mbaya zaidi

19. Utatumia nambari gani **kukadiria uzoefu wako wa hivi karibuni zaidi** katika kliniki? Tumia nambari yoyote kutoka 0 hadi 10, ambapo 0 ni uzoefu mbaya zaidi wa kliniki na 10 ni uzoefu bora zaidi wa kliniki.
    a. 0 Uzoefu mbaya zaidi
    b. 1
    c. 2
    d. 3
    e. 4
    f. 5
    g. 6
    h. 7
    i. 8
    j. 9
    k. 10 Uzoefu bora zaidi

20. Jinsia yako ni gani?
    a. Kiume
    b. Kike
    c. Nyingine

21. Unazungumza vyema Kiingereza kwa kiasi gani?
    a. Vizuri sana
    b. Vizuri
    c. Si vizuri
    d. Sio hata kidogo

22. Utakadiriaje afya yako kwa ujumla?
    a. Vizuri zaidi
    b. Vizuri Sana
    c. Vizuri
    d. Wastani
    e. Mbaya

Asante kwa kuchukua muda kushiriki katika utafiti huu. Tutakutumia kadi ya zawadi ya kielektroniki ya $10 kwenye **anwani ya barua pepe yako au kwenye simu yako ya mkononi** ndani ya saa 48 zijazo. Tafadhali onyesha ni njia gani inakufanyia kazi kwa ubora zaidi.

a) Barua pepe
b) Maandishi

Anwani yako ya barua pepe ya kutuma kadi ya zawadi ya kielektroniki ya $10 ni gani?

Nambari yako ya simu ya mkononi ya kutuma kadi ya zawadi ya kielektroniki ya $10 ni gani?

Je, unapendelea kujadili uzoefu wako wa hivi karibuni zaidi wa huduma ya afya kwa maelezo zaidi na mhojaji? Tutatoa **kadi ya zawadi ya kielektroniki ya $25** zaidi kwa ushiriki wako katika mahojiano ya siri ya dakika 30.

a) Ndiyo, ninapendelea
b) Hapana, sipendelei

Ili kusaidia kupangia mahojiano yako yajayo:

Nambari ya simu bora ya kukufikia ni gani?

Anwani ya barua pepe bora ya kukufikia ni gani?

Mtafiti atakupigia simu ndani ya wiki mbili zijazo kwa wakati unaokufaa wewe. Tafadhali chagua **vidirisha vyote vya saa vinavyoendana** na ratiba yako. Saa zimeorodheshwa katika Saa Kamili ya Pasifiki (Saa ya California).

a) Siku za wiki (Jumatatu hadi Ijumaa) saa 5 asubuhi hadi saa 8 mchana
b) Siku za wiki (Jumatatu hadi Ijumaa) saa 9 alasiri hadi saa 12 jioni
c) Jumamosi saa 3 asubuhi hadi saa 7 mchana
d) Nyingine

Iwapo ulichagua “nyingine”, **ni siku gani za wiki na saa** zitakuwa bora zaidi kwa ratiba yako?

Asante kwa nia yako ya kushiriki katika mahojiano. Tafadhali chagua tarehe na saa kutoka kwa **kalenda hii** ili kuratibu mahojiano yako. Utatumiwa ujumbe wa uthibitisho kabla ya mahojiano yako.

Kuisha:
1) "Utafiti umeisha kwa sababu hukuridhia kushiriki. Ikiwa bado unataka kushiriki, tafadhali weka upya kivinjari chako ili kuanzisha tena utafiti. Kiungo cha karatasi ya taarifa ya utafiti kimejumuishwa **hapa**."

2) "Asante kwa kukamilisha utafiti na kwa nia yako ya kushiriki katika mahojiano. Maoni yako yatasaidia kuboresha huduma za wagonjwa kama wewe. Tarehe na saa yako kamili ya mahojiano itathibitishwa na wewe katika siku zijazo. Kiungo cha karatasi ya taarifa ya utafiti kimejumuishwa **hapa**."

3) "Asante kwa kukamilisha utafiti. Majibu yako yatasaidia kuboresha huduma za wagonjwa kama wewe. Kiungo cha karatasi ya taarifa ya utafiti kimejumuishwa **hapa**."

Tagalog

Survey sa Pasyente

Anong **wika** ang sinasalita mo sa bahay?

Arabic
Armenian
Burmese
Cambodian (Central Khmer)
Chinese (Cantonese)
Chinese (Mandarin)
Dari
Farsi
Haitian Creole
Hindi
Hmong
Japanese
Karen
Korean
Laotian
Oromo
Pashto
Portuguese
Punjabi
Russian
Somali
Spanish
Swahili
Tagalog
Thai
Tigrinya
Ukrainian
Vietnamese
Iba pa

1. Ang iyong petsa ng kapanganakan

2. Petsa ng pinakahuli mong pagbisita sa klinika:

3. **Pinakinggan ka ba nang mabuti** ng provider?
    a. Oo, tiyak ito
    b. Oo, medyo
    c. Hindi

4. **Nagpakita ba ng paggalang** ang provider sa kung ano ang kailangan mong sabihin?
    a. Oo, tiyak ito
    b. Oo, medyo
    c. Hindi

5. **Hinikayat ka bang magtanong** ng provider?
    a. Oo, tiyak ito
    b. Oo, medyo
    c. Hindi

6. **Naglaan ba ng sapat na oras** sa iyo ang provider?
    a. Oo, tiyak ito
    b. Oo, medyo
    c. Hindi

7. **Tinulungan ka ba ng interpreter na ipaliwanag** ang nararamdaman mo sa provider?
    a. Oo, tiyak ito
    b. Oo, medyo
    c. Hindi

8. **Tinulungan ka ba ng interpreter na maunawaan ang mga tagubilin** na mula sa provider?
    a. Oo, tiyak ito
    b. Oo, medyo
    c. Hindi

9. Tinrato ka ba ng interpreter nang may **paggalang at respeto**?
    a. Oo, tiyak ito
    b. Oo, medyo
    c. Hindi

10. Gamit ang anumang numero mula 0 hanggang 10, kung saan 0 ay ang pinakamasamang interpreter na posible at 10 bulang pinakamahusay na interpreter na posible, **anong numero ang gagamitin mo para markahan ang interpreter na ito?**
    a. 0 Pinakamasamang interpreter na posible
    b. 1
    c. 2
    d. 3
    e. 4
    f. 5
    g. 6
    h. 7
    i. 8
    j. 9
    k. 10 Pinakamahusay na interpreter na posible

11. Binilinan ka ba ng provider na bumalik para sa follow-up na appointment?
    a. Oo
    b. Hindi

12. Naka-iskedyul ba ang interpreter para sa follow-up na appointment mo?
    a. Oo
    b. Hindi
    c. Hindi ko alam

13. Binilinan ka ba ng provider na ipapunan ang reseta mo para sa gamot?
    a. Oo
    b. Hindi

14. **Tinulungan ka ba ng interpreter na maunawaan** kung paano iinumin ang gamot?
    a. Oo, tiyak ito
    b. Oo, medyo
    c. Hindi

15. Alam mo bang maisasalin ng interpreter ang mga tagubilin para sa pag-inom ng inireresetang gamot?
    a. Oo
    b. Hindi

16. Sa nakaraang 12 buwan, nagkaroon ka ba ng appointment na **walang** interpreter?
    a. Oo
    b. Hindi

17. Sino ang tumulong sa pag-interpret para sa iyo noong wala kang interpreter? Lagyan ng tsek ang lahat ng naaangkop.
    a. Kaibigan
    b. Kapamilya
    c. Tauhan sa klinika

18. Paano maikukumpara ang interpreter sa appointment mo kamakailan sa mga **nakaraan** mong appointment na walang interpreter?
    a. Mas mabuti
    b. Halos pareho
    c. Mas masama

19. Anong numero ang gagamitin mo para **markahan ang karanawan mo pinaka-kamakailan** sa klinika? Gamitin ang anumang numero mula 0 hanggang 10, kung saan 0 ay ang pinakamasamang karanasan sa klinika na posible at 10 bulang pinakamabuting karanasan sa klinika na posible, anong numero ang gagamitin mo para markahan ang interpreter na ito?
    a. 0 Pinakamasamang karanasan na posible
    b. 1
    c. 2
    d. 3
    e. 4
    f. 5
    g. 6
    h. 7
    i. 8

    j. 9
    k. 10 Pinakamabuting karanasan na posible

20. Ano ang kasarian mo?
    a. Lalaki
    b. Babae
    c. Iba pa

21. Gaano ka kahusay magsalita ng Ingles?
    a. Mahusay na mahusay
    b. Mahusay
    c. Hindi Mahusay
    d. Talagang hindi

22. Paano mo ire-rate ang kalusugan mo sa pangkalahatan?
    a. Napakahusay
    b. Masyadong mahusay
    c. Mahusay
    d. Katamtaman
    e. Hindi Mahusay

Salamat sa panahon mo sa pagsali sa pag-aaral na ito. Magpapadala kami sa iyo ng $10 na electronic gift card sa **email address mo o sa cell phone mo sa pamamagitan ng text** sa loob ng susunod na 48 oras. Pakilagay ang paraang gumagana para sa iyo.

a) Mag-email
b) Mag-text

Ano ang email address mo na padadalhan ng $10 na electronic gift card?

Ano ang numero ng cell phone mo na padadalhan ng $10 na electronic gift card?

Interesado ka bang pag-usapan ang karanasan mo sa pangangalagang pangkalusugan kamakailan nang mas detalyado sa isang panayam? Magbibigay kami ng dagdag na **$25 na electronic gift card** para sa pagsali mo sa isang kumpidensyal na 30-minutong panayam.

a) Oo, interesado ako
b) Hindi, hindi ako interesado

Para makatulong sa pagplano para sa paparating mong panayam:

Ano ang pinakamainam na **numero ng telepono** para makontak ka?

Ano ang pinakamainam na **email address** para makontak ka?

Tatawagan ka ng isang mananaliksik sa loob ng susunod na dalawang lunggo sa oras na matatawagan ka. Pakipiliin ang **lahat ng oras na pwede** sa iskedyul mo. Ang mga oras ay nakalista sa Pamantayang Oras sa Pasipiko (Oras sa California).

a) Mga araw mula Lunes hanggang Biyernes 11AM-2PM
b) Mga araw mula Lunes hanggang Biyernes 3PM-6PM
c) Sabado 9AM-1PM
d) Iba pa

Kung pinili mo ang "iba pa", **anong mga araw at oras sa linggo** ang pinakamabuti para sa iskedyul mo?

Salamat sa interes mo sa pagsali sa isang panayam. Pakipili ang petsa at oras mula sa **kalendaryong ito** para iiskedyul ang panayam mo. Magpapadala sa iyo ng nagkukumpirmang mensahe bago ang panayam mo.

Mga Katapusan:
1) "Natapos ang survey na ito dahil hindi ka pumayag na sumali. Kung gusto mo pa ring sumali, paki-refresh ang browser mo para umpisahan muli ang survey. Kasama **rito** ang link sa impormasyon tungkol sa pag-aaral."

2) Salamat sa pagkumpleto mo sa survey at sa interes mo sa pagsali sa isang panayam. Makakatulong ang opinion mo na mapabuti ang mga serbisyo para sa mga pasyenteng kagaya mo. Ang eksaktong petsa at oras ng panayam ay kukumpirmahin sa iyo sa mga darating na araw. Kasama **rito** ang link sa impormasyon tungkol sa pag-aaral."

3) "Salamat sa pagkumpleto ng survey. Makakatulong ang mga sagot mo na mapabuti ang mga serbisyo para sa mga pasyenteng kagaya mo. Kasama **rito** ang link sa impormasyon tungkol sa pag-aaral."

Thai

แบบสอบถามผู้ป่วย

คุณพูด**ภาษา**อะไรเมื่ออยู่ที่บ้าน

อาหรับ
อาร์เมเนีย
พม่า
กัมพูชา (เขมรภาคกลาง)
จีน (กวางตุ้ง)
จีน (กลาง)
ดารี
ฟาร์ซี
ครีโอลเฮติ
ฮินดู
ม้ง
ญี่ปุ่น
กะเหรี่ยง
เกาหลี
ลาว
โอโรโม
ปัชโต
โปรตุเกส
ปัญจาบ
รัสเซีย
โซมาลี
สเปน
สวาฮีลี
ตากาล็อก
ไทย
ทิกรินยา
ยูเครน
เวียดนาม
ภาษาอื่น

1. วันเดือนปีเกิดของคุณ:

2. วันที่คุณไปคลินิกตามนัดครั้งล่าสุด:

3. แพทย์หรือผู้ให้บริการ**ตั้งใจฟัง**คุณใช่หรือไม่
    a. ใช่แน่ ๆ
    b. ใช่บางส่วน
    c. ไม่ใช่

4. แพทย์หรือผู้ให้บริการ**แสดงความใส่ใจ**ในสิ่งที่คุณพูดใช่หรือไม่
    a. ใช่แน่ ๆ
    b. ใช่บ้าง
    c. ไม่ใช่

5. แพทย์หรือผู้ให้บริการ**สนับสนุนให้คุณถามคำถาม**ใช่หรือไม่
    a. ใช่แน่ ๆ
    b. ใช่บางส่วน
    c. ไม่ใช่

6. แพทย์หรือผู้ให้บริการ**ใช้เวลากับคุณมากพอ**ใช่หรือไม่
    a. ใช่แน่ ๆ
    b. ใช่บ้าง
    c. ไม่ใช่

7. ล่าม**ช่วยคุณอธิบาย**ความรู้สึกของคุณให้แพทย์หรือผู้บริการฟังใช่หรือไม่
    a. ใช่แน่ ๆ
    b. ใช่บางส่วน
    c. ไม่ใช่

8. ล่าม**ช่วยคุณให้เข้าใจคำแนะนำ**ของแพทย์หรือผู้ให้บริการใช่หรือไม่
    a. ใช่แน่ ๆ
    b. ใช่บ้าง
    c. ไม่ใช่

9. ล่ามปฏิบัติต่อคุณอย่าง**สุภาพและให้เกียรติ**คุณใช่หรือไม่
    a. ใช่แน่ ๆ
    b. ใช่บางส่วน
    c. ไม่ใช่

10. ขอให้ใช้ตัวเลขใดก็ได้ตั้งแต่ 0 ถึง 10 เมื่อ 0 หมายถึง ล่ามที่แย่ที่สุด และ 10 หมายถึง ล่ามที่ดีที่สุด **คุณจะใช้ตัวเลขใดในการให้คะแนนล่ามคนนี้**
    a. 0 ล่ามที่แย่ที่สุด
    b. 1
    c. 2
    d. 3
    e. 4
    f. 5
    g. 6
    h. 7
    i. 8
    j. 9
    k. 10 ล่ามที่ดีที่สุด

11. แพทย์หรือผู้ให้บริการขอให้คุณกลับมาพบอีกครั้งเพื่อติดตามผลใช่หรือไม่
    a. ใช่
    b. ไม่ใช่

12. ได้มีการจัดล่ามให้สำหรับการพบเพื่อติดตามผลใช่หรือไม่
    a. ใช่
    b. ไม่ใช่
    c. ฉันไม่ทราบ

13. แพทย์หรือผู้ให้บริการบอกให้คุณซื้อยาตามใบสั่งแพทย์ใช่หรือไม่
    a. ใช่
    b. ไม่ใช่

14. ล่าม**ช่วยคุณให้เข้าใจ**วิธีใช้ยาใช่หรือไม่
    a. ใช่แน่ ๆ
    b. ใช่บ้าง
    c. ไม่ใช่

15. คุณทราบหรือไม่ว่า ล่ามสามารถแปลคำแนะนำในการใช้ยาที่ซื้อตามใบสั่งแพทย์ให้แก่คุณได้
    a. ใช่
    b. ไม่ใช่

16. ในช่วง 12 เดือนที่ผ่านมา คุณพบแพทย์หรือผู้ให้บริการ**โดยไม่มี**ล่ามใช่หรือไม่
    a. ใช่
    b. ไม่ใช่

17. เมื่อไม่มีล่าม ใครเป็นคนช่วยแปลให้คุณ กาทุกข้อที่ใช่
    a. เพื่อน
    b. คนในครอบครัว
    c. เจ้าหน้าที่ของคลินิก

18. การพบแพทย์หรือผู้ให้บริการโดยมีล่ามเป็นอย่างไร เมื่อเทียบกับการพบ**ครั้งก่อน ๆ** ของคุณที่ไม่มีล่าม
    a. ดีกว่า
    b. พอ ๆ กัน
    c. แย่กว่า

19. คุณจะใช้ตัวเลขอะไรในการ**ให้คะแนนการพบแพทย์หรือผู้ให้บริการครั้งล่าสุด**ที่คลินิกนั้น
    ขอให้ใช้ตัวเลขใดก็ได้ตั้งแต่ 0 ถึง 10 เมื่อ 0 หมายถึง ประสบการณ์ที่แย่ที่สุด และ 10 ประสบการณ์ที่ดีที่สุด
    a. 0 ประสบการณ์ที่แย่ที่สุด
    b. 1
    c. 2
    d. 3
    e. 4
    f. 5

g. 6
h. 7
i. 8
j. 9
k. 10 ประสบการณ์ที่ดีที่สุด

20. เพศของคุณคือ
    a. ชาย
    b. หญิง
    c. อย่างอื่น

21. คุณพูดภาษาอังกฤษได้ดีแค่ไหน
    a. ดีมาก
    b. ดี
    c. ไม่ดี
    d. ไม่ได้เลย

22. สุขภาพโดยรวมของคุณเป็นอย่างไร
    a. ดีเยี่ยม
    b. ดีมาก
    c. ดี
    d. ปานกลาง
    e. แย่

ขอบคุณที่สละเวลาเข้าร่วมในการสำรวจนี้ เราจะส่งบัตรกำนัลอิเล็กทรอนิกส์มูลค่า $10 ถึง**ที่อยู่อีเมลของคุณหรือเบอร์โทรศัพท์มือถือของคุณเป็นข้อความตัวหนังสือ** ภายใน 48 ชั่วโมง โปรดบอกว่าวิธีใดสะดวกที่สุดสำหรับคุณ

a) อีเมล
b) ข้อความตัวหนังสือทางโทรศัพท์

ที่อยู่อีเมลของคุณที่จะให้เราส่งบัตรกำนัลอิเล็กทรอนิกส์มูลค่า $10 ให้คืออะไร

เบอร์โทรศัพท์มือถือของคุณที่จะให้เราส่งบัตรกำนัลอิเล็กทรอนิกส์มูลค่า $10 ให้คืออะไร

คุณสนใจที่จะพูดคุยอย่างละเอียดมากขึ้นเกี่ยวกับการพบแพทย์หรือผู้ให้บริการด้านการรักษาพยาบาลเมื่อเร็ว ๆ นี้กับผู้สัมภาษณ์หรือไม่ เราจะมอบ**บัตรกำนัลอิเล็กทรอนิกส์มูลค่า $25** ให้อีกใบ ถ้าคุณเข้าร่วมการสัมภาษณ์ที่เป็นความลับนี้เป็นเวลา 30 นาที

a) ฉันสนใจ
b) ฉันไม่สนใจ

เพื่อช่วยวางแผนสำหรับการสัมภาษณ์ที่จะมีขึ้นของคุณ:

เบอร์โทรศัพท์ที่สะดวกที่สุดในการติดต่อคุณคืออะไร

ที่อยู่อีเมลดีที่สะดวกที่สุดในการติดต่อคุณคืออะไร

นักวิจัยจะโทรศัพท์ถึงคุณภายในสองสัปดาห์ข้างหน้าตามเวลาที่สะดวกที่สุดสำหรับคุณ
โปรดเลือก**ทุกช่วงเวลาที่สะดวก**สำหรับคุณ เวลาที่แสดงเป็นเวลามาตรฐานแปซิฟิก (เวลาในรัฐแคลิฟอร์เนีย)

a) วันธรรมดา (จันทร์ถึงศุกร์) เวลา 11.00-14.00 น.
b) วันธรรมดา (จันทร์ถึงศุกร์) เวลา 15.00-18.00 น.
c) วันเสาร์ เวลา 9.00-13.00 น.
d) อย่างอื่น

ถ้าคุณเลือก “อย่างอื่น” **วันและเวลาใด**ที่สะดวกที่สุดสำหรับคุณ

ขอบคุณที่สนใจเข้าร่วมการสัมภาษณ์ โปรดเลือกวันที่และเวลาจาก**ปฏิทินนี้** เพื่อทำนัดสัมภาษณ์คุณ
เราจะส่งข้อความยืนยันถึงคุณก่อนการสัมภาษณ์ของคุณ

ตอนจบ:
1) "การสำรวจสิ้นสุดลงแล้ว เพราะคุณไม่ยินยอมเข้าร่วม ถ้าคุณยังต้องการเข้าร่วม
โปรดรีเฟรชเบราว์เซอร์ของคุณเพื่อเริ่มแบบสอบถามอีกครั้ง ลิงก์ถึงเอกสารข้อมูลการสำรวจอยู่**ที่นี่**"

2) "ขอบคุณที่ตอบแบบสอบถามและที่สนใจเข้าร่วมการสัมภาษณ์ คำตอบของคุณจะช่วยปรับปรุงบริการต่าง ๆ
สำหรับผู้ป่วยเช่นคุณ จะมีการยืนยันวันและเวลาสัมภาษณ์ที่แน่นอนของคุณกับคุณในอีกไม่กี่วันข้างหน้า
ลิงก์ถึงเอกสารข้อมูลการสำรวจอยู่**ที่นี่**"

3) "ขอบคุณที่ตอบแบบสอบถามนี้ คำตอบของคุณจะช่วยปรับปรุงบริการต่าง ๆ สำหรับผู้ป่วยเช่นคุณ
ลิงก์ถึงเอกสารข้อมูลการสำรวจอยู่**ที่นี**

Tigrinya

ናይ ተሓካሚ መጽናዕቲ

ኣብ ገዛኹም እንታይ **ቋንቋ** ትዛረቡ፧

ዓረብኛ ኣርመንኛ
በርመዝ
ካምቦዲያ (ማእከላይ ክህመር) ቻይና
(ካንቶናኛ)
ቻይና (ማንዳሪን) ዳሪ
ፋርሲ
ሀይቲ ክሪኦል ህንዲ
ህሞንግ ጃፓን ካረን
ኮሪያ ላኦሽያኛ
ኦሮሞኛ ፓሽቶኛ
ፖርቱጋልኛ ፑንጃቢ ራሻ
ሶማልኛ ስፓንሽ
ስዋሂሊ ታጋሎግ ታይ
ትግርኛ
ዩክረይን ቬትናሚዝ
ካልእ

1. ዕለተ ልደትኩም፡

2. ናትኩም ናይ ቀረባ ግዜ ናይ ክሊኒክ ቆጸራ ዝነበረ ዕለት፡

3. እቲ ወሃቢ ኣገልግሎት **ብጥንቃቐ ሰሚዑኩም** ድዩ፧
    a. እወ፡ ብርግጽ
    b. እወ፡ ብመጠኑ
    c. ኣይፋል

4. እቲ ወሃቢ ኣገልግሎት ነቲ ክትብልዎ ዝነበረኩም **ኣኽብሮት ኣርእዩ** ድዩ፧
    a. እወ፡ ብርግጽ
    b. እወ፡ ብመጠኑ
    c. ኣይፋል

5. እቲ ወሃቢ ኣገልግሎት **ሕቶታት ክትሓቱ ኣተባቢዕኩም’ዶ**፧
    a. እወ፡ ብርግጽ
    b. እወ፡ ብመጠኑ
    c. ኣይፋል

6. ወሃቢ ኣገልግሎት **ምሳኹም እኹል ግዜ ኣሕሊፉ'ዶ፧**
    a. እወ፡ ብርግጽ
    b. እወ፡ ብመጠኑ
    c. ኣይፋል

7. እቲ ተርጓማይ ነቲ ወሃቢ ኣገልግሎት **ከመይ ይስመዓኩም ከም ዝነበረ ንኽትገልጹ** ሓጊዝኩም'ዶ፧
    a. እወ፡ ብርግጽ
    b. እወ፡ ብመጠኑ
    c. ኣይፋል

8. እቲ ተርጓማይ **ካብቲ ወሃቢ ኣገልግሎት ዝወሃብ መምርሒታት ንምርዳእ** ሓጊዝኩም'ዶ፧
    a. እወ፡ ብርግጽ
    b. እወ፡ ብመጠኑ
    c. ኣይፋል

9. እቲ ተርጓማይ **ብትሕትናን ኣኽብሮትን** ድዩ ዝሕግዘኩም ነይሩ፧
    a. እወ፡ ብርግጽ
    b. እወ፡ ብመጠኑ
    c. ኣይፋል

10. ካብ 0 ክሳብ 10 ዘሎ ዝኾነ ቁጽሪ ብምጥቃም፡ ማለት 0 ዝኸፍአ ኣስተርጓሚ ክኸውን ከሎ፡ 10 ድማ ዝበለጸ ተርጓማይ ይኸውን፡ **ነዚ ተርጓማይ ንምግምጋም እንታይ ቁጽሪ ምተጠቐምኩም፧**
    a. 0 ዝኸፍአ ኣስተርጓሚ ክኸውን ከሎ
    b. 1
    c. 2
    d. 3
    e. 4
    f. 5
    g. 6
    h. 7
    i. 8
    j. 9
    k. 10 ዝበለጸ ተርጓማይ ይኸውን

11. እቲ ወሃቢ ኣገልግሎት፡ ንዝቐጽል ናይ ምክትታል ቆጸራ ንኽትምለሱ ሓቲቱኩም'ዶ፧
    a. እወ
    b. ኣይፋል

12. ኣብቲ ዝቕጽል ናይ ምክትታል ቆጸራ ተርጓማይ ተመዲብልኩም ድዩ፧
    a. እወ
    b. ኣይፋል
    c. ኣይፈለጥኩን

13. እቲ ወሃቢ ኣገልግሎት፡ ናይ ሓኪም ትእዛዝ መድሃኒት ክትወስ ነጊሩኩም'ዶ፧
    a. እወ
    b. ኣይፋል

14. እቲ ተርጓማይ ብኸመይ መድሃኒት ክትወስዱ ከምዘለኩም **ንምርዳእ ሓጊዝኩም'ዶ፧**
    a. እወ፡ ብርግጽ
    b. እወ፡ ብመጠኑ
    c. ኣይፋል

15. ተርጓማይ ነቲ ብትእዛዝ ናይ ሓኪም ንኽትወስድዎ ዝወሃብ መድሃኒት፡ ነቲ መምርሒታት ክትርጉም ከም ዝኽእል ትፈልጡ'ዶ፧
    a. እወ
    b. ኣይፋል

16. ኣብ ዝሓለፉ 12 ኣዋርሕ፡ **ብዘይ ተርጓማይ** ቆጸራ ኔርኩም ድዩ፧
    a. እወ
    b. ኣይፋል

17. ተርጓማይ ኣብ ዘይነበረኩም እዋን፡ መን እዩ ኣብ ምትርጓም ሓጊዝኩም፧ ንኹሉ እቲ ዝምልከት ምረጹ
    a. መሓዛ
    b. ኣባል ስድራ
    c. ሰራሕተኛታት ክሊኒክ

18. እዚ ናይ ቀረባ ግዜ ብ ተርጓማይ ዝነበረ ቆጸራኹም ምስቲ ናይ **ዝሓለፈ** ብዘይ ተርጓማይ ዝነበረ ቆጸ ኹም ብምንጽጻር፡ ከመይ ነይሩ፧
    a. ዝሓሸ
    b. ዳርጋ ተመሳሳሊ
    c. ዝኸፍአ

19. ኣብ'ዚ ክሊኒክ ኣብ ናይ ቀረባ ግዜ ዝነበረ ተመክሮኹም **ንምግምጋም እንታይ ቁጽሪ ምተጠቐምኩም፧** ካብ 0 ክሳብ 10 ዘሎ ዝኾነ ቁጽሪ ተጠቐሙ፡ እዚ ማለት 0 ዝኸፍአ ናይ ክሊኒክ ተሞክሮ ክኸውን ከሎ፡ 10 ድማ ዝበለጸ ናይ ክሊኒክ ተሞክሮ ይኸውን።

a. 0 ዝኸፍአ ተመክሮ ክኸውን ከሎ
b. 1
c. 2
d. 3
e. 4
f. 5
g. 6
h. 7
i. 8
j. 9
k. 10 ዝበለጸ ተመክሮ ይኸውን

20. ጾታኹም እንታይ እዩ፧
   a. ተባዕታይ
   b. ኣንስተይቲ
   c. ካልእ

21. እንግሊዝኛ ክሳብ ክንደይ ኢኹም ግርም ጌርኩም ትዛረቡ፧
   a. ኣዝዩ ጽቡቕ
   b. ጽቡቕ
   c. ጽቡቕ ኣይኮነን
   d. ብፍጹም ኣይዛረብን

22. ሓፈሻዊ ጥዕናኹም ብኸመይ ትግምግምዎ፧
   a. ብሉጽ
   b. ብጣዕሚ ጽቡቕ
   c. ጽቡቕ
   d. ደሓን
   e. ሕማቕ

ኣብዚ መ ናዕ ንምስታፍ ግዜ ስለ ዝወሰድኩም ነመስግነኩም። ኣብ ዝመጽእ 48 ሰዓታትውሽጢ ንሕና ናይ $10 ኤሌክትሮኒካዊ ናይ ህያብ ካርድ ናብ **ናይ ኢመይል ኣድራሻኹም ወይ ናብ ሞባይል ተሌፎንኩም ብጽሑፍ**ክንልእከልኩም ኢና። ብኽብረትኩም ኣየናይ ኣገባብ ብዝበለጸ ከምዝሰርሓልኩም ኣነጽሩ።
a) ኢመይል
b) ጽሑፍ ናይ ኢድ ስልኪ

ነቲ $10 ኤለክትሮኒካዊ ናይ ህያብ ካርድ ንምልኣኽ እንታይ እዩ ናይ ኢመይል ኣድራሻኹም፧

ነቲ $10 ኤለክትሮኒካዊ ህያብ ካርድ ንምልኣኽ ክንደይ እዩ ቁጽሪ ሞባይልኩም፧

ብዛዕባ እቲ ኣብ ቀረባ እዋን ዘጋጠመኩም ናይ ክንክን ጥዕና ተመክሮ ምስ ሓደ ቃለ-መሕትት ዝገበር ሰብ ብዝርዝር ክትመያየጡ ትደልዩ'ዶ፧ ኣብ ናይ 30 ደቓይቕ ምስጢራዊ ዝኾነ ቃለ መሕትት ንኽትሳተፉ ተወሳኺ **ናይ $25 ኤሌክትሮኒካዊ ናይ ህያብ ካርድ** ክንህብ ኢና።

a) እወ፡ ተገዳስነት ኣሎኒ
b) ኣይፋል፡ ኣይግደስን እየ

ኣብ ዝመጽእ ናይ ቃለ-መሕትት መደብ ንምግባር ንምሕጋዝ፡

እቲ ዝበለጸ ክንረኽበኩም እንኽእል **ቁጽሪ ተሌፎን** ክንደይ እዩ፧

እቲ ዝበለጸ ክንረኽበኩም እንኽእል **ናይ ኢመይል ኣድራሻ** እንታይ እዩ፧

ሓደ ተመራማሪ ከኣ ኣብቲ ንዓኹም ዝጥዕመኩም ግዜ፡ ኣብ ውሽጢ'ዘን ዝቕጽላ ክልተ ሰሙን ክድውለልኩም እዩ። ብኽብረትኩም **ምስ ኩሉ ዘለኩም መደባት** ዝጥዕም ግዜ ምረጹ። ሰዓታት ድማ ብናይ ፓሲፊክ መዐቀኒ ግዜ (ካሊፎርንያ ግዜ) ተዘርዚሮም ኣለዉ።

a) መዓልታት ናይ ሰሙን (ካብ ሰኑይ ክሳዕ ዓርቢ) 11 ቅ.ቀ -2 ድ.ቀ
b) መዓልታት ናይ ሰሙን (ካብ ሰኑይ ክሳብ ዓርቢ) 3 ድ.ቀ - 6 ድ.ቀ
c) ቀዳም 9 ቅ.ቀ - 1 ድ.ቀ
d) ካልእ

"ካልእ" እንተ መሪጽኹም፡ **ኣብ ኣየኖት መዓልትታት ናይ ሰሙንን ሰዓታትን** እዩ ዝጥዕመኩም፧

ኣብ ቃለ-መሕትት ክትሳተፉ ስለ ዝደለኹም ነመስግነኩም። ብኽብረትኩም ቃለ-መሕትትኩም ንምምዳብ ካብዚ **<u>ዓውደ ኣዋርሕ</u>** እዚ ዕለትን ሰዓትን ምረጹ። ቅድሚ ቃለ-መሕተትኩም ድማ ናይ መረጋገጺ መልእኽቲ ክስደደልኩም እዩ።

መዘዘሚ፡
1) "እቲ መጽናዕቲ ኣብቂዑ እዩ፡ ምኽንያቱ ኽትሳተፉ ፍቓደኛታት ኣይኮንኩምን። ሕጂ ውን ኣብዚ ኽትሳተፍ እንተ ደሊኹም፡ ብኽብረትኩም ነቲ መጽናዕቲ ከም ብሓድሽ ንምጅማር ነቲ ናይ ኢንተርነት መኽፈቲ እንደገና ኣጀምርዎ። መስመር ርክብ ናይቲ ናይ ሓበሬታ ወረቐት መጽናዕቲ **<u>ኣብዚ</u>** ተጠቓሊሉ ኣሎ።"

2) "ነቲ መጽናዕቲ ስለ ዝወዳእክምዎን ኣብቲ ቃለ-መሕትት ኽትሳተፉ ስለ ዝደለኹምን ነመስግነኩም። ግብረ መልሲኹም ድማ ንከማኹም ንዝኣመሰሉ ተሓከምቲ፡ ኣገልግሎት ንምምሕያሽ ክሕግዝ እዩ። እቲ ልክዕ ናይ ቃለ- መሕትት ዕለትን ሰዓትን ከኣ ኣብ ዝመጽእ መዓልታት ምሳኹም ብምዃን ከም ዝረጋገጽ ክኸውን እዩ። መስመር ርክብ ናይቲ ናይ ሓበሬታ ወረቐት መጽናዕቲ **<u>ኣብዚ</u>** ተጠቓሊሉ ኣሎ።"

3) "ነቲ መጽናዕቲ ስለ ዝወዳእኩምዎ ነመስግነኩም። መልስታትኩም ድማ ከማኹም ንዝኣመሰሉ ተሓከምቲ፣ ኣገልግሎት ንምምሕያሽ ክሕግዝ እዩ። መስመር ርክብ ናይቲ ናይ ሓበሬታ ወረቐት መጽናዕቲ **ኣብዚ** ተጠቓሊሉ ኣሎ።"

Chinese (Traditional)

病患調查

您在家裡講什麼**語言**？

阿拉伯語

亞美尼亞語

緬甸語

柬埔寨語（中高棉）

中文（粵語）

中文（國語）

達里語

波斯語

海地克裡奧爾語

印地語

苗語

日語

凱倫語

韓語

老撾語

奧羅莫語

普什圖語

葡萄牙語

旁遮普語

俄語

索馬里語

西班牙語

斯瓦希里語

他加祿語

泰語

提格利尼亞語

烏克蘭語

越南語

其他

1. 您的出生日期：

2. 您最近一次門診看診日期：

3. 醫事人員**是否仔細**傾聽了您的意見？
    a. 是，當然
    b. 是，有點
    c. 否

4. 醫事人員是否**尊重**您說的話？
    a. 是，當然
    b. 是，有點
    c. 否

5. 醫事人員是否**鼓勵您提問**？
    a. 是，當然
    b. 是，有點
    c. 否

6. 醫事人員是否為您**花了足夠時間**？
    a. 是，當然
    b. 是，有點
    c. 否

7. 口譯員是否**幫您解釋了**您的感受給醫事人員聽？
    a. 是，當然
    b. 是，有點
    c. 否

8. 口譯員是否**幫您理解了**醫事人員的說明？
    a. 是，當然
    b. 是，有點
    c. 否

9. 口譯員對待您是否有**禮貌和尊重**？
    a. 是，當然

b. 是，有點

c. 否

10. 用 0 到 10 之間的任何數字，0 表示最差，10 表示最佳，**您會用什麼數字來評價這個口譯員**？

a. 0 表示最差口譯員
b. 1
c. 2
d. 3
e. 4
f. 5
g. 6
h. 7
i. 8
j. 9
k. 10 表示最佳口譯員

11. 醫事人員是否要求您返回複診？

a. 是

b. 否

12. 是否為您的復診安排了口譯員？

a. 是

b. 否

c. 不知道

13. 醫事人員是否告訴您去拿處方藥？

a. 是

b. 否

14. 口譯員是否**幫您明白了**如何服藥？

a. 是，當然

b. 是，有點

c. 否

15. 您是否知道口譯員可以翻譯服用處方藥說明？

a. 是

b. 否

16. 在過去的 12 個月中，您是否有過**沒有**口譯員的看診？

a. 是
b. 否

17. 沒有口譯員時，誰幫您翻譯？勾選所有適用項。
    a. 朋友
    b. 家人
    c. 診所工作人員

18. 您最近有口譯員的看診與**過去**沒有口譯員的看診相比如何？
    a. 更好
    b. 差不多
    c. 更差

19. 您會用什麼數字來**評價您最近的診所體驗**？用 0 到 10 之間的任何數字，0 表示最差，10 表示最佳。
    a. 0 表示最差體驗
    b. 1
    c. 2
    d. 3
    e. 4
    f. 5
    g. 6
    h. 7
    i. 8
    j. 9
    k. 10 表示最佳體驗

20. 您的性別？
    a. 男
    b. 女
    c. 其他

21. 您的英語說得怎麼樣？
    a. 很好
    b. 好
    c. 不好
    d. 一點也不會

22. 您如何評價您的整體健康狀況？
    a. 極佳
    b. 很好
    c. 好
    d. 不錯
    e. 很差

感謝您抽出寶貴時間參與這項研究。在接下來的 48 小時內，會將一張 10 美元電子禮品卡發送到**您的電子郵件地址或通過簡訊發送到您的手機**。請說明您喜歡使用哪種方法。

a) 電子郵件
b) 簡訊

您用於接收 10 美元電子禮品卡的電子郵件地址？

您用於接收 10 美元電子禮品卡的手機號碼？

您是否有興趣與訪談員詳談您最近的醫護服務體驗？會另外給一張 **25 美元的電子禮品卡**，感謝您花 30 分鐘參加保密訪談。

a) 是，我有興趣
b) 否，我沒有興趣

為了幫您計劃即將到來的訪談：

哪個**電話號碼**最容易聯絡上您？

哪個**電子郵件地址**最容易聯絡上您？

在接下來的兩週內，研究人員會在您方便時給您打電話。請選擇**適合您日程安排**的所有時間段。所列時間使用太平洋標準時間（加州時間）。

a) 平日（週一至週五）上午 11 點至下午 2 點
b) 平日（週一至週五）下午 3 點至下午 6 點
c) 週六上午 9 點至下午 1 點
d) 其他

如果選擇了「其他」，**一週中哪些日子和時間**對您最方便？

感謝您有興趣參加訪談。請從**此日曆**中選擇一個日期和時間安排您的訪談。訪談前會給您發送確認資訊。

結束語：

1）「由於您不同意參與，調查已結束。如果您仍想參與，請刷新瀏覽器重新開始調查。**此處**包含研究資訊表的連結。」

2) 「感謝您完成調查並有興趣參加訪談。您的反饋將有助於為像您這樣的病患改善服務。在未來幾天內會與您確認您的確切訪談日期和時間。**此處**包含研究資訊表的連結。」

3) 「感謝您完成調查。您的回覆將有助於改善為像您這樣的病患提供的服務。**此處**包含研究資訊表的連結。」

Ukrainian

Опитування пацієнта

Якою **мовою** Ви розмовляєте вдома?

Арабською
Вірменською
Бірманською
Камбоджійською (Центральний Хмер)
Китайською (Кантонська)
Китайською (мандарин)
Дарі
Фарсі
Гаїтянською креольською
Хінді
Хмонг
Японською
Карен
Корейською
Лаоською
Оромо
Пушту
Португальською
Пенджабі
Російською
Сомалійською
Іспанською
Суахілі
Тагалог
Тайською
Тигриня
Українською
В’єтнамською
Іншою

1. Ваша дата народження:

2. Дата Вашого останнього візиту до клініки:

3. Чи постачальник медичних послуг **слухав** Вас **уважно**?
   a. Так, безумовно
   b. Так, певною мірою
   c. Ні

4. Чи постачальник медичних послуг **виявив повагу** до того, що Ви сказали?
   a. Так, безумовно
   b. Так, певною мірою
   c. Ні

5. Чи постачальник медичних послуг **заохочував Вас ставити питання**?
   a. Так, безумовно

    b. Так, певною мірою
    c. Ні

6. Чи постачальник медичних послуг **приділив** Вам **досить часу**?
    a. Так, безумовно
    b. Так, певною мірою
    c. Ні

7. Чи перекладач **допоміг Вам пояснити** постачальникові послуг, як Ви почуваєтесь?
    a. Так, безумовно
    b. Так, певною мірою
    c. Ні

8. Чи перекладач **допоміг Вам зрозуміти інструкції** від постачальника послуг?
    a. Так, безумовно
    b. Так, певною мірою
    c. Ні

9. Чи перекладач ставився до Вас **ввічливо та з повагою**?
    a. Так, безумовно
    b. Так, певною мірою
    c. Ні

10. Користуючись цифрами від 0 до 10, де 0 означає найгіршого перекладача, якого можна уявити, та 10 означає найкращого перекладача, якого можна уявити, **яку цифру Ви б використали, щоб оцінити цього перекладача**?
    a. 0 Найгірший перекладач, якого можна уявити
    b. 1
    c. 2
    d. 3
    e. 4
    f. 5
    g. 6
    h. 7
    i. 8
    j. 9
    k. 10 Найкращий перекладач, якого можна уявити

11. Чи попросив Вас постачальник послуг прийти на повторний прийом?
    a. Так
    b. Ні

12. Чи було призначено перекладача для Вашого повторного прийому?
    a. Так
    b. Ні
    c. Я не знаю

13. Чи попросив Вас постачальник послуг отримати ліки за рецептом?
    a. Так
    b. Ні

14. Чи перекладач **допоміг Вам зрозуміти**, як приймати ліки?
    a. Так, безумовно
    b. Так, певною мірою
    c. Ні

15. Чи знали Ви, що перекладач може перекладати інструкції для прийняття ліків, отримацих за рецептом?
    a. Так
    b. Ні

16. Протягом останніх 12 місяців чи були Ви на прийомі **без** перекладача?
    a. Так
    b. Ні

17. Хто допоміг перекладати для Вас, коли Ви не мали перекладача? Позначте все, що підходить.
    a. Друг
    b. Член родини
    c. Персонал клініки

18. Як пройшов Ваш недавній прийом у лікаря з перекладачем порівняно до Ваших **попередніх** прийомів без перекладача?
    a. Краще
    b. Приблизно однаково
    c. Гірше

19. Якою цифрою Ви б скористалися, щоб **оцінити Ваше останнє перебування** у клініці? Використайте будь-яку число від 0 до 10, де 0 означає найгірший досвід перебування, який можна уявити, та 10 означає найкращий досвід перебування, який можна уявити.
    a. 0 Найгірший досвід перебування, який можна уявити
    b. 1
    c. 2
    d. 3
    e. 4
    f. 5
    g. 6
    h. 7
    i. 8
    j. 9
    k. 10 Найкращий досвід перебування, який можна уявити

20. Ваша стать?
    a. Чоловік
    b. Жінка
    c. Інше

21. Як добре Ви володієте англійською?
    a. Дуже добре
    b. Добре
    c. Не дуже добре
    d. Зовсім не володію

22. Як би Ви оцінили загальний стан свого здоров'я?
    a. Прекрасний
    b. Дуже добрий
    c. Добрий
    d. Прийнятний
    e. Поганий

Дякуємо за Ваш час, виділений для участі у цьому дослідженні! Ми надішлемо Вам електронну подарункову картку на $10 **на Вашу електронну адресу або на Ваш мобільний телефон шляхом короткого текстового повідомлення (SMS)** протягом наступних 48 годин. Будь ласка, вкажіть, якому методу Ви віддаєте перевагу.

a) Email
b) Текстове повідомлення

Вкажіть, на яку електронну адресу надіслати Вашу електронну подарункову картку на $10.

Вкажіть, на який номер телефону надіслати Вашу електронну подарункову картку на $10.

Чи зацікавить Вас можливість більш детального обговорення свого недавнього досвіду в медичному закладі з людиною, яка проводить опитування? Ми надамо додаткову **електронну подарункову картку на $25** за Вашу участь у конфіденційній 30-ти хвилинній співбесіді.

a) Так, мене цікавить
b) Ні, мене не цікавить

Щоб допомогти запланувати Вашу наступну співбесіду:

На який **номер телефону** потрібно телефонувати для контакту з Вами?

На яку **адресу електронної пошти** написати контакту з Вами?

Дослідник зателефонує Вам протягом двох тижнів в той час, коли Вам зручно. Будь ласка, оберіть **всі проміжки часу, які Вам підходять** з огляду на Ваш розклад. Вказаний час є Тихоокеанським Стандартним Часом (Pacific Standard Time) (час Каліфорнії).

a) Робочі дні (з понеділка по п'ятницю) 11:00-14:00
b) Робочі дні (з понеділка по п'ятницю) 15:00-18:00
c) Субота 09:00-13:00
d) Інший

Якщо Ви обрали «Інший», **які дні тижня та проміжки часу** найкраще підходять з огляду на Ваш розклад?

Дякуємо за Вашу зацікавленість щодо участі у співбесіді. Будь ласка, оберіть дату та час у **цьому календарі**, щоб призначити свою співбесіду. Повідомлення з підтвердженням буде надіслано Вам до співбесіди.

Закінчення:
1) «Опитування припинилося, бо Ви не дали своєї згоди на участь. Якщо Ви однак бажаєте взяти участь, будь ласка, перевантажте свій браузер, щоб почати опитування наново. Посилання на інформаційну сторінку опитування надано **тут**.»

2) «Дякуємо за те, що Ви пройшли опитування та за Вашу зацікавленість щодо участі у співбесіді. Ваш відгук допоможе покращити послуги для пацієнтів, таких, як Ви. Дату та час Вашої співбесіди буде остаточно узгоджено з Вами протягом наступних днів. Посилання на інформаційну сторінку опитування надано **тут**.»

3) «Дякуємо за те, що Ви пройшли опитування. Ваш відгук допоможе покращити послуги для пацієнтів, таких, як Ви. Посилання на інформаційну сторінку опитування надано **тут**.»

Vietnamese

Khảo Sát Bệnh Nhân

Quý vị sử dụng **ngôn ngữ** nào ở nhà?

Tiếng Ả Rập
Tiếng Armenia
Tiếng Miến Điện
Tiếng Campuchia (Khmer)
Tiếng Trung (tiếng Quảng Đông)
Tiếng Trung (Quan Thoại)
Tiếng Dari
Tiếng Farsi
Tiếng Creole của Haiti
Tiếng Hindi
Tiếng Hmong
Tiếng Nhật
Tiếng Karen
Tiếng Đại Hàn
Tiếng Lào
Tiếng Oromo
Tiếng Pashto
Tiếng Bồ Đào Nha
Tiếng Punjabi
Tiếng Nga
Tiếng Somali
Tiếng Tây Ban Nha
Tiếng Swahili
Tiếng Tagalog
Tiếng Thái
Tiếng Tigrinya
Tiếng Ukraine
Tiếng Việt
Khác:

1. Ngày sinh của quý vị:

2. Ngày hẹn khám gần đây nhất của quý vị:

3. Nhà cung cấp có **cẩn thận lắng nghe** quý vị không?
   a. Vâng, có.
   b. Vâng, một phần nào
   c. Không

4. Nhà cung cấp có **thể hiện sự tôn trọng** đối với những gì quý vị phải nói không?
   a. Vâng, có.
   b. Vâng, một phần nào
   c. Không

5. Nhà cung cấp có **khuyến khích quý vị đặt câu hỏi** không?
   a. Vâng, có.

    b. Vâng, một phần nào
    c. Không

6. Nhà cung cấp có **dành đủ thời gian** với quý vị không?
    a. Vâng, có.
    b. Vâng, một phần nào
    c. Không

7. Phiên dịch có **giúp quý vị giải thích** quý vị cảm thấy như thế nào với nhà cung cấp không?
    a. Vâng, có.
    b. Vâng, một phần nào
    c. Không

8. Phiên dịch có **giúp quý vị hiểu các hướng dẫn** của nhà cung cấp không?
    a. Vâng, có.
    b. Vâng, một phần nào
    c. Không

9. Phiên dịch có đối xử quý vị với sự **lịch sự và tôn trọng** không?
    a. Vâng, có.
    b. Vâng, một phần nào
    c. Không

10. Sử dụng một số bất kỳ từ 0 đến 10, trong đó 0 là phiên dịch kém nhất và 10 là phiên dịch tốt nhất, **quý vị sẽ sử dụng số nào để đánh giá phiên dịch**?
    a. 0 Phiên dịch kém nhất
    b. 1
    c. 2
    d. 3
    e. 4
    f. 5
    g. 6
    h. 7
    i. 8
    j. 9
    k. 10 Phiên dịch tốt nhất

11. Nhà cung cấp có yêu cầu quý vị quay lại để tái khám không?
    a. Có
    b. Không

12. Phiên dịch có được lên lịch vào cuộc hẹn tái khám của quý vị không?
    a. Có
    b. Không
    c. Tôi không biết

13. Nhà cung cấp có yêu cầu quý vị điền đơn thuốc không?
    a. Có
    b. Không

14. Phiên dịch có **giúp quý vị hiểu** cách dùng thuốc không?

  a. Vâng, có.
  b. Vâng, một phần nào
  c. Không

15. Quý vị có biết là phiên dịch có thể dịch hướng dẫn dùng thuốc theo toa không?
  a. Có
  b. Không

16. Trong 12 tháng qua, quý vị có cuộc hẹn khám nào mà **không có** phiên dịch không?
  a. Có
  b. Không

17. Ai đã giúp dịch cho quý vị khi quý vị không có phiên dịch? Đánh dấu tất cả những câu trả lời phù hợp.
  a. Một người bạn
  b. Thành viên trong gia đình
  c. Nhân viên y tế

18. Cuộc hẹn gần đây có phiên dịch so với những cuộc hẹn **trước đây** không có phiên dịch của quý vị như thế nào?
  a. Tốt Hơn
  b. Không khác
  c. Tệ Hơn

19. Quý vị sẽ sử dụng số nào để **đánh giá trải nghiệm gần nhất của quý vị** tại phòng khám? Sử dụng một số bất kỳ từ 0 đến 10, trong đó 0 là trải nghiệm phòng khám tệ nhất và 10 là trải nghiệm phòng khám tốt nhất.
  a. 0 trải nghiệm phòng khám tệ nhất
  b. 1
  c. 2
  d. 3
  e. 4
  f. 5
  g. 6
  h. 7
  i. 8
  j. 9
  k. 10 trải nghiệm phòng khám tốt nhất

20. Giới tính của quý vị là gì?
  a. Nam
  b. Nữ
  c. Khác:

21. Quý vị nói tiếng Anh thành thạo ở mức nào?
  a. Rất thành thạo
  b. Thành thạo
  c. Không thành thạo
  d. Không chút nào

22. Quý vị sẽ đánh giá sức khỏe tổng thể của mình như thế nào?

a. Tuyệt vời
b. Rất Tốt
c. Tốt
d. Trung Bình
e. Kém

Cảm ơn quý vị đã dành thời gian tham gia khảo sát. Chúng tôi sẽ gửi quý vị một thẻ quà tặng điện tử trị giá $10 đến **địa chỉ email của quý vị hoặc đến điện thoại di động của quý vị qua tin nhắn** trong vòng 48 giờ tới. Vui lòng nêu rõ phương thức nào phù hợp nhất với quý vị.

a) Email
b) Tin Nhắn

Địa chỉ email của quý vị được dùng để gửi thẻ quà tặng điện tử $10 là gì?

Số điện thoại di động của quý vị được dùng để gửi thẻ quà tặng điện tử $10 là gì?

Quý vị có muốn thảo luận chi tiết hơn về trải nghiệm dịch vụ y tế gần đây của quý vị với một người phỏng vấn không? Chúng tôi sẽ cung cấp thêm một **thẻ quà tặng điện tử trị giá $25** đối với việc tham gia vào một cuộc phỏng vấn được bảo mật 30 phút của quý vị.

a) Có, tôi muốn.
b) Không, tôi không muốn.

Để giúp lập chương trình phỏng vấn sắp tới của quý vị:

Số điện thoại dễ liên lạc nhất với quý vị là gì?

Địa chỉ email dễ liên lạc nhất với quý vị là gì?

Một nhà nghiên cứu sẽ gọi cho quý vị trong vòng hai tuần tới vào thời điểm phù hợp với quý vị. Vui lòng chọn **tất cả những khung thời gian phù hợp** với lịch của quý vị. Thời gian được nêu theo Múi Giờ Thái Bình Dương (giờ California).

a) Các ngày trong tuần (Thứ Hai - Thứ Sáu) 11:00 sáng - 2:00 chiều
b) Các ngày trong tuần (Thứ Hai - Thứ Sáu) 3:00 chiều - 6:00 chiều
c) Thứ Bảy 9:00 sáng - 1:00 chiều
d) Khác:

Nếu quý vị chọn “khác”, **những thời gian nào và ngày nào trong tuần** phù hợp nhất với lịch của quý vị?

Cảm ơn quý vị đã quan tâm tham gia phỏng vấn. Vui lòng chọn ngày và thời gian **trên lịch này** để lên lịch phỏng vấn. Một thông báo xác nhận sẽ được gửi đến quý vị trước cuộc phỏng vấn.

Kết thúc:
1) “Cuộc khảo sát đã kết thúc vì quý vị không đồng ý tham gia. Nếu quý vị vẫn muốn tham gia, vui lòng refresh (làm mới) trang để khởi động lại cuộc khảo sát. Một đường dẫn tới bảng thông tin nghiên cứu có **ở đây**.”

2) “Cảm ơn quý vị đã hoàn thành khảo sát và đã quan tâm tham gia phỏng vấn. Sự phản hồi của quý vị sẽ giúp cải thiện dịch vụ cho những bệnh nhân như quý vị. Ngày và thời gian phỏng vấn chính xác

của quý vị sẽ được xác nhận với quý vị trong vài ngày tới. Một đường dẫn tới bảng thông tin nghiên cứu có **ở đây**.”

3) “Cảm ơn quý vị đã hoàn thành khảo sát. Những câu trả lời của quý vị sẽ giúp cải thiện dịch vụ cho những bệnh nhân như quý vị. Một đường dẫn tới bảng thông tin nghiên cứu có **ở đây**.”
